# Generalised quantum computational spectroscopy on a quantum chip

**DOI:** 10.1038/s41467-026-74936-7

**Published:** 2026-07-11

**Authors:** Chonghao Zhai, Jinzhao Sun, Jieshan Huang, Jun Mao, Hongchang Bao, Siyuan Zhang, Vlatko Vedral, Xiao Yuan, Jianwei Wang

**Affiliations:** 1 State Key Laboratory for Mesoscopic Physics, School of Physics, Peking University, Beijing, China; 2 School of Physical and Chemical Sciences, Queen Mary University of London, London, UK; 3 Clarendon Laboratory, University of Oxford, Oxford, UK; 4 Center on Frontiers of Computing Studies, School of Computer Science, Peking University, Beijing, China; 5 Frontiers Science Center for Nano-optoelectronics & Collaborative Innovation Center of Quantum Matter, Peking University, Beijing, China; 6 Collaborative Innovation Center of Extreme Optics, Shanxi University, Taiyuan, China; 7 Hefei National Laboratory, Hefei, China

**Keywords:** Quantum simulation, Quantum information, Quantum optics

## Abstract

Spectroscopy underpins modern scientific discovery across diverse disciplines. While experimental spectroscopy probes material properties through scattering or radiation measurements, computational spectroscopy combines theoretical models with experimental data to predict spectral properties, essential for advancements in physics, chemistry, and materials science. However, quantum systems present unique challenges for computational spectroscopy due to their inherent complexity, and current quantum algorithms remain largely limited to static and closed quantum systems. Here, we present and demonstrate a generalised quantum computational spectroscopy that lifts these limitations by reconstructing the quantum autocorrelation function via an ancilla-assisted Hadamard test quantum circuit. Our method is applicable to a broad range of quantum systems, including closed, open, and time-dependent driven quantum systems. We experimentally validate this approach, which leverages arbitrary controlled quantum dynamics and efficient classical noise-mitigation strategy, on a programmable silicon-photonic quantum processing chip, capable of high-fidelity time-evolution simulations. The versatility of our method is demonstrated through spectroscopic computations for diverse quantum systems, revealing novel phenomena such as parity-time symmetry breaking and topological holonomy that are inaccessible to conventional spectroscopy or quantum eigenstate algorithms. This work establishes a noise-robust methodology for quantum spectral analysis.

## Abstract (web summary)

A generalised quantum computational spectroscopy method is proposed for closed, open, and driven quantum systems, and experimentally validated on a programmable silicon-photonic chip, resolving PT-symmetry breaking and topological holonomy.

## Introduction

Probing spectral characteristics of physical systems serves as a cornerstone in the fields of condensed matter physics, materials science, and biochemistry, underpinning diverse applications across modern science and technology. Experimental spectroscopy^1–3^ provides a standard technique for probing spectral information through external perturbations and the spectroscopic response to those excitations is analysed via spectrometers. For example, vibrational spectroscopy resolves molecular structures via frequency-dependent absorption of light^1^, and electron spectroscopy determines material electronic structures through energy-resolved measurements^2,3^. Complementarily, computational spectroscopy provides atomic-level insights into real and hypothetical materials with precise control and inherent coherence. It hence enables the prediction and analysis of spectroscopic properties under experimentally challenging conditions and accelerates material discovery by pre-synthesis simulation. This capability holds significant importance for applications in molecular engineering^4,5^, drug design^6^, advanced material synthesis^7^, and investigation of many-body physics^8,9^. Nevertheless, calculating spectroscopy of quantum systems by classical means poses significant challenges-exponentially scaling computational resources are required for modelling quantum dynamics and in particular solving eigenstate problems.

Quantum computers^10^ have the potential to efficiently analyze the spectroscopic characteristics of quantum systems^5,6,9^. Quantum algorithms, including quantum phase estimations^11–14^(QPE), variational quantum eigensolvers^15–17^(VQE), and their combinations^18^ have been proposed to prepare eigenstates on quantum computers. Phase estimations and its generalisation in the framework of quantum signal processing^19,20^ require deep quantum circuits for enough precision to estimate eigenenergies, while variational methods suffer from prolonged and often inefficient optimizations^21^. Recent advances in linear response theory^22^, spectral filtering^23^, and algorithmic shadow spectroscopy^24^ also offer alternative pathways, only estimating the excitation spectrum as classical methods in both experimental and computational spectroscopy. More critically, these methods^22–24^ encounter a fundamental limitation when applied to more general quantum systems governed by non-Hermitian or time-dependent Hamiltonians. In these systems, eigenstates may lose orthogonality, and spectral properties deviate from the standard paradigm. For non-Hermitian Hamiltonians, damping and amplifying effects can broaden spectral lines and dramatically alter spectral intensities^25^. For time-dependent Hamiltonians, the spectrum can acquire complex band structures with dense, even temporally evolving features^26^. This makes eigenenergy identification and property estimation intrinsically difficult. While other methods for eigenstate characterization exist^27–31^, they fall outside the scope of this general spectroscopic context. This gap underscores the critical need for a spectral estimator capable of addressing general quantum dynamics – an advancement that would open new frontiers in the study of open quantum systems, non-equilibrium dynamics, and topological phenomena through quantum spectral estimation.

In this work, we experimentally demonstrate a generalised quantum computational spectroscopy (QCS). This approach leverages a core algorithmic strategy-reconstructing time correlation functions via an ancilla-assisted Hadamard test^32^-to evaluate a defined quantum autocorrelation function (QACF), thereby enabling the estimation of eigenstate-resolved spectral properties for general quantum systems. The QACF eliminates interference between distinct frequency components arising from different dynamical phases during time evolution, effectively suppressing spurious spectral cross-terms caused by excitations and restoring eigenstate orthogonality information. When combined with classical techniques (e.g., Gaussian windowing and singular spectrum renormalization), the QACF suppresses experimental random noise-which predominantly resides in low-order temporal correlations-yielding robust and high-resolution spectral estimation. We report a proof-of-principle experimental realisation of QCS on a programmable silicon-photonic quantum chip, which can implement arbitrary controlled-unitaries with high fidelity and exceptional programmability. This device unlocks direct spectral characterisation of diverse quantum systems – including Heisenberg spin model, non-Hermitian Hamiltonian, and periodically driven system. We experimentally observe intriguing properties, including gapped eigenstates reconstruction (resolving non-orthogonal eigenstates), non-Hermitian parity-time (PT) symmetry breaking (detected through spectral transitions), and topological holonomy in periodically driven systems (inferred from their spectral structures). Notably, QCS achieves what was previously inaccessible: it estimates arbitrary observable expectation values under gapped eigenstates in diverse quantum dynamics and deciphers intricate eigen-properties from a spectroscopic approach.

## Results

### The generalised QCS

**Fig. 1:**
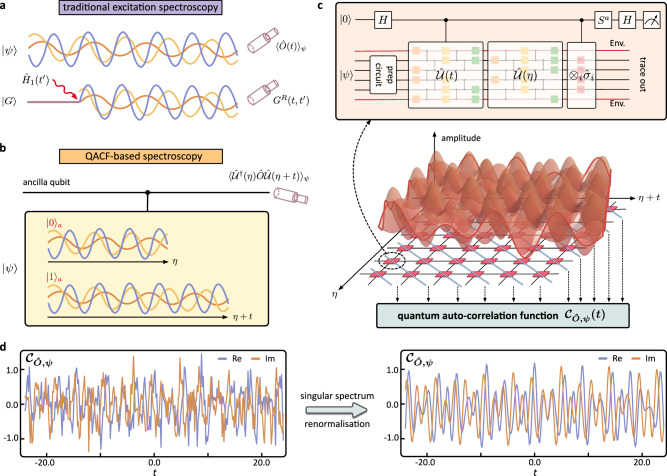
The generalised quantum computational spectroscopy. Comparison of **a** traditional excitation spectroscopy, and **b** QACF-based spectroscopy: In (a), traditional excitation spectroscopy probes only the oscillation frequencies corresponding to eigenenergy differences. It relies on Fourier transforms of either probe-observable’s dynamics ${\langle \hat{O}(t)\rangle }_{\psi }=\langle \psi | {\hat{U}}^{{{\dagger}} }(t)\hat{O}\hat{U}(t)| \psi \rangle$, or, retarded Green’s function ${\hat{H}}_{1}(t{\prime} )$, ${G}^{R}(t,t^{\prime} )=-i\theta (t-t^{\prime} )\langle [\hat{O}(t),{\hat{H}}_{1}(t^{\prime} )]\rangle$ that encode linear response to perturbation ${\hat{H}}_{1}(t^{\prime} )$, where *θ*(*t*) is the Heaviside function and [ ⋅ , ⋅ ] denotes the operator commutator. The averages in retarded Green’s function are evaluated in the ground state $\left\vert G\right\rangle$. In (**b**), QACF-based spectroscopy employs quantum control circuits to simultaneously evolve target dynamics through time interval *η* and *η* + *t*. Unlike the traditional spectroscopy, QACF-based spectroscopy maintains entire quantum coherence without external excitation. Measurement on the ancilla qubits allows efficient estimation of QACF estimator as defined in Eq.(1). **c** Quantum circuit of generalised Hadamard test for implementing (**b**). It consists of preparation circuits, a controlled time evolution $\hat{U}(t)$, a normal time evolution $\hat{U}(\eta )$ and a controlled Pauli-string observable ${\otimes }_{i}{\hat{\sigma }}_{i}$, ${\hat{\sigma }}_{i}\in \{\hat{I},{\hat{\sigma }}^{x},{\hat{\sigma }}^{y},{\hat{\sigma }}^{z}\}$. The time series are sampled at discretised time stamps in the two-dimensional space of *η* and *η* + *t*, with the sum of terms along different diagonals (white lines) yielding the QACF. The Hadamard gate is denoted as *H* and a phase-gate *S* = *diag*{1, − *i*} is applied to estimate the real (*a* = 0) and imaginary (*a* = 1) part of the non-Hermitian observable. The environment qubits (represented by red lines in circuits) are used for open quantum system simulation. **d** Simulated process of the noise-mitigation method, singular spectrum renormalisation, which can faithfully recover the oscillating signals of QACF ${{{{\mathcal{C}}}}}_{\hat{O},\psi }(t)$ from the considerable random noise background, greatly enhancing the noise robustness of QCS. Re (Im) in labels denotes the real (imaginary) part of the QACF.

Traditional spectroscopy without quantum controlled operations mainly measures excitations by probing the response dynamics to a perturbation ${\hat{H}}_{1}(t^{\prime} )$, characterised by either the time-varying observable ${\langle \hat{O}(t)\rangle }_{\psi }$ or the retarded Green’s function ${G}^{R}(t,t^{\prime} )$ (Fig. 1a). However, this approach struggles to resolve fine eigenstate properties in generic quantum systems. To overcome these limitations, we develop a methodology based on a prior time-correlation proposal^32^ that reconstructs time correlation functions via an ancilla-assisted Hadamard test to estimate a quantum autocorrelation function:

1 $${{{{\mathcal{C}}}}}_{\hat{O},\psi }(t)=\int{{{\rm{d}}}}\eta {{{\mathcal{G}}}}(\eta,\tau )\left\langle \psi \right\vert {\hat{U}}^{{{\dagger}} }(\eta )\hat{O}\hat{U}(\eta+t)\left\vert \psi \right\rangle,$$

where ${{{\mathcal{G}}}}(\eta,\tau )=\exp [-{\eta }^{2}/(2{\tau }^{2})]/\sqrt{2\pi }\tau,\tau > 0$ is the Gaussian window to avoid infinite integration, $\hat{U}(t)={{{\mathcal{T}}}}{e}^{-i\int_{0}^{t}\hat{H}(t^{\prime} ){{{\rm{d}}}}t^{\prime} }$ is the time-evolution operator for a general Hamiltonian $\hat{H}(t)$ (${{{\mathcal{T}}}}$ is the time-ordering operator), $\hat{O}$ represents a probe operator, and $\left\vert \psi \right\rangle$ is the initial state (*ℏ* = 1). The integrand in Eq. (1) can be efficiently estimated using controlled time-evolution circuits enhanced by coherent quantum controls (Fig. 1b). Quantum processor implementing the generalised Hadamard test (Fig. 1c) enables efficient computation of the QACF. The quantum circuit consists of controlled time evolution $\hat{U}(t)$, time evolution $\hat{U}(\eta )$, and controlled Pauli-string operators that contribute to the calculation of arbitrary observables $\hat{O}$ via Pauli decomposition. Despite measurement noise, the clear oscillatory signal can be effectively recovered through singular spectrum renormalisation of time-series data (Fig. 1d, see Supplementary Note 2). Finally, by applying the Fourier transform to ${{{{\mathcal{C}}}}}_{\hat{O},\psi }(t)$, we can resolve the frequency (*ω*)-domain distribution of eigen-spectrum ${\widetilde{{{{\mathcal{C}}}}}}_{\hat{O},\psi }(\omega )$. Additionally, time-frequency methods like the short-time Fourier transform can also be employed to resolve time-varying spectral features.

Generally, the time evolution of any quantum state can be described as a superposition of its Fourier components: $\hat{U}(t)\left\vert \psi \right\rangle={\sum }_{n}{c}_{n}{e}^{-i{E}_{n}t}\left\vert {u}_{n}\right\rangle$, where *E*_*n*_ and $\left\vert {u}_{n}\right\rangle$ respectively denote the quasi-eigenenergy and quasi-eigenstate obtained by Fourier transform, with *c*_*n*_ as normalisation coefficients. The physical interpretation of quasi-eigenenergies and quasi-eigenstates is detailed in Methods. Crucially, for systems described by non-Hermitian or time-dependent Hamiltonians, these quasi-eigenstates generally form one non-orthogonal set. For clarity, we here focus on systems with gapped and real quasi-eigenenergies, while a detailed discussion of eigenenergies with imaginary parts is provided in Supplementary Note 3.4. The QACF can be simplified significantly, as cross-terms between different Fourier components vanish due to the property of auto-correlation function. This results in only oscillations at the quasi-eigenenergies:

2 $${{{{\mathcal{C}}}}}_{\hat{O},\psi }(t)={\sum}_{n,m}{e}^{-\frac{1}{2}{({E}_{n}-{E}_{m})}^{2}{\tau }^{2}}{c}_{n}^{*}{c}_{m}{e}^{-i{E}_{n}t}\left\langle {u}_{n}\right\vert \hat{O}\left\vert {u}_{m}\right\rangle,$$

where the QACF completely eliminates the oscillating signal at excitation frequency of (*E*_*n*_ − *E*_*m*_) and the approximate orthogonality is formed by an exponential factor ${e}^{-\frac{1}{2}{({E}_{n}-{E}_{m})}^{2}{\tau }^{2}}$. Hence, the quasi-eigenenergies appear as distinct peaks (diagonal terms with amplitude $| {c}_{n}{| }^{2}\langle {u}_{n}| \hat{I}| {u}_{n}\rangle$) in the Fourier transform of the identity-operator QACF, ${\widetilde{{{{\mathcal{C}}}}}}_{\hat{I},\psi }(\omega )$, allowing direct extraction of any observable’s expectation value via ${\langle \hat{O}\rangle }_{{u}_{n}}=\langle {u}_{n}| \hat{O}| {u}_{n}\rangle /\langle {u}_{n}| {u}_{n}\rangle={\widetilde{{{{\mathcal{C}}}}}}_{\hat{O},\psi }({E}_{n})/{\widetilde{{{{\mathcal{C}}}}}}_{\hat{I},\psi }({E}_{n})$, where $\hat{I}$ denotes the identity operator.

A Gaussian window function with unit norm in ***L***^2^-space is selected for the QACF and the Fourier transform of the QCS, ensuring faithful spectral extraction and high energy resolution for time evolution. For reliable spectral computation, the window width *τ* must satisfy $\tau \Delta {E}_{\min }\gg 1$, where $\Delta {E}_{\min }$ is the smallest energy gap of interest. By truncating the time integral to the interval [ − 4*τ*, 4*τ*], we achieve a truncation error below 0.01%. Moreover, we discretise the time integral in accordance with the Nyquist-Shannon sampling theorem^33^, enabling the faithful discrete-time window Fourier transform of the time series data. Regarding noise suppression, crucially, any oscillating noise at frequency *f*_noise_ distinct from the quasi-eigenstates is strongly suppressed by QACF with the exponential factor $\exp (-{\tau }^{2}{({E}_{n}-{f}_{{{{\rm{noise}}}}})}^{2}/2)$ for each quasi-eigenenergy *E*_*n*_. Uncorrelated noise arising from experimental quantum gate implementations is also mitigated by QACF owing to its low temporal correlations. Remaining random noise from measurements can be further mitigated through singular spectrum renormalisation of the time series^34^, which enhances the noise robustness of QCS. QCS relies on the synergistic combination of quantum and classical resources. The quantum processor executes controlled-unitary evolutions to measure QACF, providing the intrinsic phase evolution (${e}^{-i{E}_{n}t}$) required for eigen-spectrum estimation. In contrast, performance enhancements such as spectral resolution and noise robustness are achieved classically: Gaussian windowing suppresses spectral leakage from finite-time truncation, while singular spectrum renormalisation filters out hardware and shot noise. This hybrid pipeline effectively circumvents the stringent coherence limits of purely digital algorithms. Given a truncation error *ϵ*_1_ and a statistical measurement error *ϵ*_2_, QCS estimates properties of gapped eigenstates with the total complexity of ${{{\mathcal{O}}}}\big[{(\sqrt{2{{{\rm{ln}}}}(1/{\epsilon }_{1})}R(\hat{H})/\Delta {E}_{\min })}^{2}/{\epsilon }_{2}^{2}\big]$, where $R(\hat{H})$ refers to the spectral radius of the Hamiltonian, and such complexity scales polynomially with system size for local Hamiltonians (details are provided in Supplementary Note 1).

The QCS provides an effective resolution to the challenge of extending quantum spectral estimation to general quantum dynamics. Complete elimination of spurious oscillatory signals arising from excitations, combined with strong suppression of random noise via classical techniques, yields a clean and stable eigenspectrum across diverse systems. The capability of QCS enables distinct applications: for time-independent Hermitian Hamiltonians, it facilitates accurate observable estimation and quantum state tomography of individual eigenstates; for non-Hermitian Hamiltonians, it preserves distinct spectral peaks despite non-orthogonal eigenstates, enabling clear identification of phenomena such as PT symmetry-breaking transitions^35^; and for time-dependent Hamiltonians, it can resolve complex band structures and fine sub-band energy levels for probing spectral fingerprints of topological holonomy^26^.

### A programmable silicon-photonic quantum chip

**Fig. 2:**
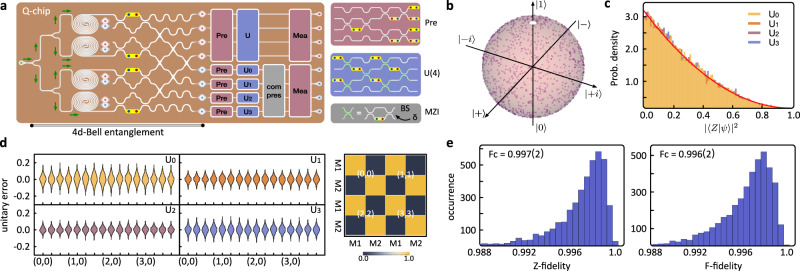
Characterisation of the programmable silicon-photonic quantum chip. **a** Quantum chip diagram for arbitrary controlled-unitary U(4) operation. A 4-dimensional entangled Bell state of two photons is generated on-chip by simultaneously pumping four quantum light sources through the spontaneous four-wave mixing process. The red idler photon serve as the ancilla qudit, while the blue signal photon act as the target qudit. Purple inset: circuit for state preparation (its reverse operation functions as a projection measurement). Blue inset: arbitrary unitary operations U(4) (including U_0_-U_3_) are implemented using a square-arranged interferometer network of MZIs and phase shifters, which collectively realise U(2). Gray inset: circuit diagram of an MZI, consisting of one phase shifter and two beam-splittes (BSs) with splitting angle error *δ*. The controlled-unitary operation is achieved by applying the space expansion (purple boxes) and coherent compression processes (gray box) on the target qudit state. **b** Coverage of U(2) operations on the Bloch sphere. Nearly the entire sphere is accessible, except for a small forbidden region (white area) caused by beam splitter imperfections, characterized by *δ* with an average value of 0.019(9) for 108 MZIs. This leads to a forbidden area ratio *A*_*FB*_/4*π* = 0.14%, corresponding to an extinction ratio about 30dB. **c** Experimental (*Q*(*x*)) and theoretical (*P*(*x*)) probability density of ∣〈*Zψ*〉∣^2^, where $\left.| \psi \right\rangle$ represents an ensemble of four-dimensional random states generated by Haar-random unitary sampling and $\left.| Z\right\rangle$ denotes the computational basis {$\left.| 0\right\rangle,\left.| 1\right\rangle,\left.| 2\right\rangle,\left.| 3\right\rangle$}. Ideally, the probability density follows *P*(*x*) = (*D* − 1)(1−*x*)^*D*−2^ (red line). The fidelity is defined as ${{{\mathcal{F}}}}(P,Q)=\int\sqrt{P(x)Q(x)}{{{\rm{d}}}}x=0.999.$ **d** Measured error distributions for unitary U_0_-U_3_, and corresponding error correlation matrices. Each matrix element’s error is analysed at position (*i*, *j*). The Haar-random unitary ensemble is partitioned into two subsets (Mat1 and Mat2) to estimate the error correlations. **e** Measured classical fidelity (F_c_) distribution for Haar-random U(4) operations in the computational basis (left) and the Fourier basis (right, defined as $\left\vert j\right\rangle=\frac{1}{2}\mathop{\sum }_{k=0}^{3}{e}^{2\pi i*jk/4}\left\vert k\right\rangle$, where *j* = 0, 1, 2, 3) across all four interferometer networks, showing high fidelity operations.

While the QCS is platform-independent and can be implemented on any quantum computing platform, we here validate it on a programmable silicon-photonic quantum processor capable of implementing controlled dynamics with high-degree arbitrariness and high-level fidelity. The quantum chip illustrated in Fig. 2a is devised to implement the generalised Hadamard test in Fig. 1c, the core operation of QCS, and fabricated by complementary metal oxide semiconductor (CMOS) processes (see Methods). Harnessing multidimensional quantum operations of integrated quantum photonic technologies^36,37^, this chip is able to generate four-dimensional Bell state of signal and idler photons as ${\left\vert \Phi \right\rangle }_{{{{\rm{Bell}}}}}$= $\frac{1}{\sqrt{d}}{\sum }_{i=0}^{d-1}\left\vert i\right\rangle \left\vert i\right\rangle$, *d*= 4. Then applying the Hilbert space expansion and coherent compression processes on the entangled state, we obtain the controlled-unitary operation as $\frac{1}{\sqrt{d}}\mathop{\sum }_{i=0}^{d-1}{\left\vert i\right\rangle }_{a}{{\mbox{U}}}_{i}{\left\vert \psi \right\rangle }_{T}$, where *a* denotes the ancilla qudit and *T* denotes the target qudit ${\left\vert \psi \right\rangle }_{T}$. Eventually, the chip implements a multi-valued controlled-unitary quantum circuit, where four four-dimensional unitary U(4) can be arbitrarily operated. In experiment, we set U_0_, U_1_ as $\hat{U}(\eta )$ and U_3_, U_4_ as ${\otimes }_{i}{\hat{\sigma }}_{i}\cdot \hat{U}(\eta+t)$. This duplicated setting of Hadamard test can effectively mitigate errors arising from device nonuniformity^38^, by the adoption of four-dimensional qudit in the ancilla register. We first characterised the arbitrariness and fidelity of the controlled unitary realised on the quantum chip.

Arbitrary unitary operations are implemented via a square network of integrated Mach-Zehnder interferometers (MZIs)^39^. The beamsplitter (BS) angle error *δ* in each MZI constrains the operation to a subspace of the Bloch sphere (Fig. 2b). To assess the interferometer network’s performance in QCS, we evaluate several key metrics. First, we characterise the coverage *Cr*, which quantifies the likelihood of successfully implementing an arbitrary unitary matrix with the U(4) network. For a *d* × *d* network, it can be measured by $Cr={e}^{-{d}^{3}{\delta }^{2}/3}$ under a uncorrelated Gaussian error model^40^. In our U(4) network device, it achieves a coverage of 99.2%, demonstrating near-whole operation range. We next characterise the error uniformity, that evaluates whether an uncorrelated uniform error distribution model could apply across all unitary settings. This is quantified by the fidelity between experimental and theoretical probability distributions for projections onto the computational basis states ∣〈*Zψ*〉∣^2^ (*Z*={0,1,2,3}), using an ensemble of Haar-random quantum states generated by Haar-random unitary matrices^41^. As shown in Fig. 2c, experimental results from four U(4) networks exhibit strong agreement with theoretical predictions across 1000 random unitaries per network, achieving a high fidelity. Furthermore, we analyse the error distribution and correlations across all U(4) networks sampled from the Haar-random unitary ensemble in Fig. 2d. All matrix element errors remain bounded within  ± 0.2 with a standard deviation of 0.06. When examining random bipartitions of the ensemble, the errors in the four diagonal matrix elements appear to be nearly uncorrelated. Another crucial aspect is classical unitary operation fidelity, which is quantified through a modified Hilbert-Schmidt inner product ${F}_{c}({U}_{thy},{U}_{exp})=\frac{1}{d}Tr(| {U}_{thy}^{{{\dagger}} }| | {U}_{exp}| )$, where ∣ ⋅ ∣ denotes element-wise absolute values. In Fig. 2e, the fidelity distributions for both computational and Fourier bases consistently exceed 0.996.

These results show that the performance of our quantum chip is sufficient for high-fidelity implementation of nearly arbitrary controlled time-evolution operators. Crucially, the operational noise demonstrates both uniformity and absence of correlations across different unitary operations—characteristics that permit effective noise suppression through singular spectrum renormalisation. We note that while high-fidelity arbitrary controlled unitary operators remain challenging on current quantum platforms, the integrated-photonic realizations demonstrated here show this capability-even at a small-scale proof-of-principle level. Further hardware scalability in terms of both dimension and photon number faces challenges in efficient multi-photon generation, increased loss, crosstalk, and calibration overheads in the quantum circuits. A detailed analysis is provided in Supplementary Note 5.

### Time-independent Hermitian quantum systems

**Fig. 3:**
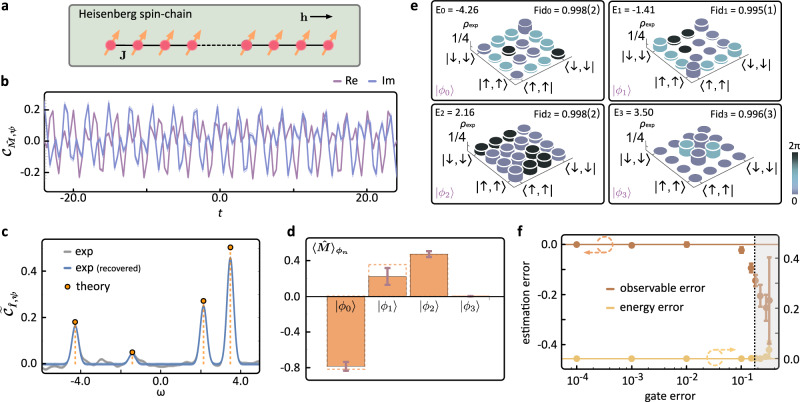
Benchmarking QCS for anisotropic Heisenberg spin-chain Hamiltonians. **a** Schematic of an anisotropic Heisenberg spin-chain with exchange coupling **J** and magnetic field **h**. Experimental validation adopted a two-spin Hamiltonian. **b** Measured QACF ${{{{\mathcal{C}}}}}_{\hat{M},\psi }(t)$ for the magnetisation operator $\hat{M}={\hat{\sigma }}_{1}^{z}\otimes {\hat{I}}_{2}+{\hat{I}}_{1}\otimes {\hat{\sigma }}_{2}^{z}$. Data points (120 time steps) are obtained through windowed auto-correlation integral of ${\langle {e}^{i\hat{H}\eta }\hat{M}{e}^{-i\hat{H}(\eta+t)}\rangle }_{\psi }$, with each point estimated from around 1000 photon-coincidence counts at discretised times *η*. Purple (blue) traces show the real (imaginary) parts, denoted by Re (Im) in labels. **c** Fourier-transformed of measured (gray lines) and recovered (blue lines) identity-operator QACF ${\widetilde{{{{\mathcal{C}}}}}}_{\hat{I},\psi }(\omega )$. The four Gaussian peaks represents the estimated eigenenergies centered at *ω* of { − 4.257, − 1.410, 2.158, 3.499}, perfectly matching with exact values *E*_*n*_ = { − 4.258, − 1.401, 2.159, 3.500} (orange dashed lines). The negligible imaginary component ( ~ 3 × 10^−5^, not shown) confirms measurement validity. Peak amplitudes correspond to eigenstate projection probabilities ∣〈*ϕ*_*n*_∣*ψ*〉∣^2^ (theory: orange points). **d** Estimation of the expectation value of magnetisation operator ${\langle \hat{M}\rangle }_{{\phi }_{n}}$ for each eigenstate $\left\vert {\phi }_{n}\right\rangle$, derived from the ratio of peak amplitudes of ${\widetilde{{{{\mathcal{C}}}}}}_{\hat{M},\psi }(\omega )$ and ${\widetilde{{{{\mathcal{C}}}}}}_{\hat{I},\psi }(\omega )$. Dashed boxes indicate theoretical values. **e** Reconstructed density matrices *ρ*_*exp*_ for four eigenstates $\left\vert {\phi }_{n}\right\rangle$ by using the QCS-enabled quantum state tomography. *E*_*n*_ denotes the estimated eigenenergy and Fid_*n*_ represents the reconstructed state fidelity. $\left.| \downarrow \right\rangle,\left.| \uparrow \right\rangle$ denote the spin-qubit basis states. **f** Error analysis for an 8-site spin-chain system implemented with QCS in noisy generalised Hadamard test circuits. We examine the ground state energy and spin-correlation operator ${\hat{\sigma }}_{1}^{z}{\hat{\sigma }}_{5}^{z}$. Gate errors (*ϵ*_*g*_) are modeled as noise following the circularly-symmetric complex Gaussian distribution ${{{\mathcal{C}}}}{{{\mathcal{N}}}}(0,{\epsilon }_{g}/(N/2))$ applied to each element of the single-step time-evolution unitary ${e}^{-i\hat{H}\delta t}$ (*δt* = 8*τ*/*N*, *N* = 120). The projection probability of ground state is denoted by the black dashed line. When the gate error exceeds the projection probability, the estimation error of ground state energy and spin-correlation increases fast (gray area). All error bands in (**b**, **c**) and error bars in (**d**) denote the  ± *σ* intervals, estimated by the Monte Carlo simulation for Poissonian sampling noise in the experiment, and by 10 independent noisy circuits in simulation in (**f**).

To validate the QCS and assess its noise robustness, we first benchmark its performance by studying spin-chain dynamics implemented on our quantum photonic chip. As a test case, we analyse the spectral properties of an anisotropic Heisenberg spin-chain Hamiltonian subject to an external magnetic field (Fig. 3a):

3 $$\hat{H}=\mathop{\sum}_{\alpha,i}{J}_{\alpha }{\hat{\sigma }}_{i}^{\alpha }{\hat{\sigma }}_{i+1}^{\alpha }+{\sum}_{\alpha,i}{h}_{\alpha }{\hat{\sigma }}_{i}^{\alpha },$$

where ${\hat{\sigma }}_{i}^{\alpha }$ (*α* = *x*, *y*, *z*, *i* = 1, 2) is the Pauli operator on the *i*-th site, **J** = [ − 1, − 1, − 1.5] denotes the exchange coupling, and **h** = [1.5, 0, 0.5] is the magnetic strength. We target at revealing the spectrum of the z-axis magnetisation operator $\hat{M}={\hat{\sigma }}_{1}^{z}\otimes {\hat{I}}_{2}+{\hat{I}}_{1}\otimes {\hat{\sigma }}_{2}^{z}$. To achieve high energy resolution, we opt for a broad Gaussian window function with *τ* = 6 for our spectral analysis. The upper bound of spectral radius follows directly from $R(\hat{H})\le {\sum }_{\alpha,i}| {J}_{\alpha }|+{\sum }_{\alpha,i}| {h}_{\alpha }|$, which yields a corresponding lower bound for the sampling frequency ${f}_{s}\ge \frac{15}{2\pi }{s}^{-1}$. On the quantum chip, the initial state is prepared in the product state as ${\left\vert \psi \right\rangle }_{T}=\left.| \uparrow \right\rangle \otimes \left.| \downarrow \right\rangle$ and the networks are mapped accordingly for the Hadamard test. We measured the quantum auto-correlation functions ${{{{\mathcal{C}}}}}_{\hat{M},\psi }(t)$ (see Fig. 3b) and ${{{{\mathcal{C}}}}}_{\hat{I},\psi }(t)$ (see Supplementary 3), when choosing a time discretisation of 120 stamps for each *t*. As reported in Fig. 3c, the eigen-spectrum for the identity operator ${\widetilde{{{{\mathcal{C}}}}}}_{\hat{I},\psi }(\omega )$ is obtained by applying a Fourier transform to the experimentally measured time-series data. This result contrasts the initial noisy data with the signal recovered after singular spectrum renormalisation, demonstrating the ability to suppress random measurement noises on the quantum chip. In the spectral distribution, the Gaussian peak amplitudes and the peak centers of recovered spectrum show an excellent agreement with theoretical predictions. The magnetization expectation values ${\langle \hat{M}\rangle }_{{\phi }_{n}}$ can be estimated as shown in Fig. 3d, where the evaluated error for ${\langle \hat{M}\rangle }_{{\phi }_{1}}$ arises as its projection probability near the gate-error floor. In addition, since the expectation value of any observable can be estimated using QCS, we applied this method to perform complete quantum state tomography. This allows for the reconstruction of density matrices for all eigenstates, as demonstrated in Fig. 3e. The results show high fidelities of Fid = {0.998(2), 0.995(1), 0.998(2), 0.996(3)}, defined as ${({\mbox{Tr}}[\sqrt{\sqrt{{\rho }_{0}}\cdot \rho \cdot \sqrt{{\rho }_{0}}}])}^{2}$, where *ρ*_0_ and *ρ* are ideal and measured eigenstates, respectively, confirming the accuracy of this approach.

We further investigate the noise resistance of QCS as the system size scales. For those dominantly uncorrelated random noise, QCS benefits from the property of QACF and the singular spectrum renormalisation, showing strong robustness when the defined gate error remains below the projection probability of the target eigenstate. To benchmark this, we simulate noisy generalised Hadamard test circuits for an 8-site spin chain with periodic boundary conditions using MindSpore Quantum^42,43^. The system is initialised in the trial state ${\left\vert \psi \right\rangle }_{T}={\left.| \downarrow \right\rangle }^{\otimes 8}$, and we estimate both the ground-state $\left\vert G\right\rangle$ energy and the spin-correlation $\left\langle G\right\vert {\hat{\sigma }}_{1}^{z}{\hat{\sigma }}_{5}^{z}\left\vert G\right\rangle$ between the two farthest sites. We simulated noisy controlled dynamics (non-unitary with error) at varying error levels, sampling 1000 shots per generalised Hadamard test. The simulation results are shown in Fig. 3f. After renormalising the singular spectrum of the sampled quantum auto-correlation time series, a small estimation error is observed until the noise approaches the ground state’s projection probability, as shown in Fig. 3f. These simulation results confirm that QCS reliably estimates eigen-spectra and eigenstate properties, provided the target eigenstate’s projection probability exceeds the gate error level. More detailed analysis is provided in Supplementary Note 1.5 and 2.2.

### Non-Hermitian quantum systems

For open quantum systems, the interaction with the environment could induce the non-Hermitian effective Hamiltonians ${\hat{H}}_{NH}$, which cannot be diagonalised by unitary like closed quantum systems. That forces a general diagonalisation in dual linear space, ${\hat{H}}_{NH}={\sum }_{i}{\lambda }_{i}\left\vert {r}_{i}\right\rangle \left\langle {l}_{i}\right\vert$ with ${\lambda }_{i}\in {\mathbb{C}}$, non-orthogonal right eigenvectors $\left\vert {r}_{i}\right\rangle$ (physical states) and their dual left counterparts $\left\langle {l}_{i}\right\vert$^25^. A notable exception is parity-time (PT) symmetric systems, which have attracted significant attention for their ability to retain real eigenenergies when in PT exact phase^35,44–46^. It provides an ideal testbed for QCS to probe open quantum system dynamics.

**Fig. 4:**
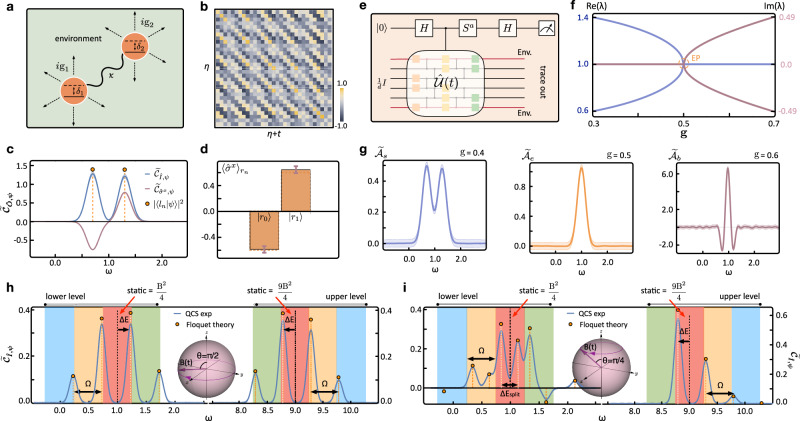
Benchmarking QCS for general dynamics in non-Hermitian and Floquet quantum systems. **a** Schematic of a non-Hermitian two-mode quantum system with detuning *δ*_1_, *δ*_2_, gain (loss) *g*_1_, *g*_2_ and coupling *κ*. **b** Measured auto-correlation matrix for the identity operator as ${\langle {e}^{i{\hat{H}}_{NH}^{{{\dagger}} }\eta }{e}^{-i{\hat{H}}_{NH}(\eta+t)}\rangle }_{\psi }$ with the gain parameter *g* = 0.4. The window sum of diagonals in the matrix gives rise to the QACF ${{{{\mathcal{C}}}}}_{\hat{I},\psi }(t)$. **c** Fourier-transformed eigen-spectra of identity operator ${\widetilde{{{{\mathcal{C}}}}}}_{\hat{I},\psi }(\omega )$ (blue lines) and Pauli-*X* operator ${\widetilde{{{{\mathcal{C}}}}}}_{{\hat{\sigma }}^{x},\psi }(\omega )$ (purple lines), peaking at {0.700, 1.302} (exact value ${\lambda }_{n}^{*}=\{0.7,1.3\}$, orange points), with amplitudes approximating the projection on the left eigenvectors ∣〈*l*_*n*_∣*ψ*〉∣^2^. **d** Estimation of the expectation value ${\langle {\hat{\sigma }}^{x}\rangle }_{{r}_{n}}$ for each physical eigenstate, derived from the ratio of peak amplitudes in (**c**). Dashed boxes indicate exact theoretical values. **e** The variant QCS circuits to track the PT phase transition. The utilizing of maximally mixed initial state (*ρ* = *I*/*d*) overcomes the plight of non-diagonalisability. **f** The change of eigenenergies in PT phase transition versus the gain parameter *g*. The real (blue line, Re(*λ*)) and imaginary (purple line, Im(*λ*)) parts of the eigenenergies coalesce at the EP. **g** Fourier-transformed eigen-spectra ${\widetilde{{{{\mathcal{A}}}}}}_{s}$ (before EP, *g* = 0.4), ${\widetilde{{{{\mathcal{A}}}}}}_{e}$ (at EP, g=0.5), ${\widetilde{{{{\mathcal{A}}}}}}_{b}$ (after EP, *g* = 0.6) of the samples from variant QCS circuits in (**e**). **h** and **i** Computation of Floquet spectrum and topological holonomy of periodically-driven systems. Fourier-transformed eigen-spectra of the identity operator ${\widetilde{{{{\mathcal{C}}}}}}_{\hat{I},\psi }(\omega )$ for a nuclear quadrupole resonance Hamiltonian with a periodically driven magnetic field **B**(*t*) at angular frequency Ω: (**h**), magnetic field circulates along the equator (*θ* = *π*/2), and (i), magnetic field follows a polar circle (*θ* = *π*/4), where *θ* denotes the zenith angle. Blue lines show experimental QCS results; orange points denote Floquet theoretical predictions. The static system (Ω = 0) has eigenenergies *B*^2^/4 and 9*B*^2^/4 (black dashed lines). Periodically driving generates the Floquet-Bloch bands with Ω-spacing (colored regimes). In **h** the energy shift Δ*E* (black arrows) from the static eigenenergy to the first-band single quasi-eigenenergy (white lines, for both lower and upper levels) enables estimation of the U(1) holonomy (Berry phase). In **i** the upper level retains a single quasi-eigenenergy band structure, while the lower level exhibits two distinct quasi-eigenenergies with splitting Δ*E*_split_. This splitting signifies genuine SU(2) holonomy, allowing extraction of the gauge-invariant Wilson loop. All error bands with spectrum lines denote the  ± *σ* intervals, estimated by the Monte Carlo simulation for Poissonian sampling noises.

To illustrate the general capability of QCS, we experimentally implement a prototypical non-Hermitian system comprising two coupled quantum modes with environmental interactions (Fig. 4a):

4 $${\hat{H}}_{NH}=\left(\begin{array}{cc}{\delta }_{1}-i{g}_{1}&\kappa \\ \kappa &{\delta }_{2}+i{g}_{2}\end{array}\right)$$

where *δ*_1_, *δ*_2_ are the detuning of two quantum modes, *g*_1_, *g*_2_ represent gain or loss for each quantum mode and *κ* is the coupling coefficient in between. In our case, we chose *δ*_1_ = *δ*_2_ = 1.0, *g*_1_ = *g*_2_ = *g* and *κ* = 0.5. When *g* < *κ* (take *g* = 0.4), the eigenenergies are real. We encode this two-mode non-Hermitian system in two optical modes (${\left\vert 1\right\rangle }_{d=4}$ and ${\left\vert 2\right\rangle }_{d=4}$) in the chip’s 4-dimensional Hilbert space. The non-unitary time evolution ${e}^{-i{\hat{H}}_{NH}t}$ is embedded within U(4) operations, initialised with state ${\left\vert \psi \right\rangle }_{T}={\left\vert 1\right\rangle }_{d=4}$. For this system, we select *τ* = 6 and sample the quantum auto-correlation function at 30 time steps for QACF and Fourier analysis. In experiment, the time correlations ${\langle {e}^{i{\hat{H}}_{NH}^{{{\dagger}} }\eta }{e}^{-i{\hat{H}}_{NH}(\eta+t)}\rangle }_{\psi }$ are measured on the quantum chip, the real part of which are partially reported in the matrix in Fig. 4b. The calculation of quantum auto-correlation function ${{{{\mathcal{C}}}}}_{\hat{I},\psi }(t)$ is realised by the windowed sum along different matrix diagonals. In addition, we also measure correlations for the parity-like operator ${\hat{\sigma }}^{x}$ in PT exact phase, which exhibits a pseudo-Hermitian relationship ${\hat{\sigma }}^{x}{\hat{H}}_{NH}{\hat{\sigma }}^{x}={\hat{H}}_{NH}^{{{\dagger}} }$ and flips sign under physical eigenstates $\left\vert {r}_{n}\right\rangle$. Fourier transform of these correlations reveals spectral peaks centered at the system’s real eigenenergies (Fig. 4c). Crucially, the peak amplitudes in ${\widetilde{{{{\mathcal{C}}}}}}_{\hat{I},\psi }(\omega )$ correspond to ∣〈*l*_*n*_*ψ*〉∣^2^ rather than conventional projection probabilities, reflecting the non-Hermitian nature of the system. Similar to the previous case, calculating the ratio of peaks between the two spectra returns the expectation values of ${\hat{\sigma }}^{x}$ (Fig. 4d), in good agreement with theoretical predictions.

Figure 4e illustrates a variant QCS quantum circuits with a single controlled time evolution and the maximally mixed initial state (*ρ* = *I*/*d*) to reveal the PT phase transition, where the system’s eigenenergies change from purely real to complex-conjugate pairs (shown in Fig. 4f). At the exceptional point (*g* = *κ*), ${\hat{H}}_{NH}$ becomes non-diagonalisable and admits only a Jordan canonical form, that is the hallmark of critical point in PT phase transition. The variant QCS circuits can still extract the diagonal terms’ oscillations even if there only exists a reduced Jordan canonical form for Hamiltonians, which represents challenging for conventional techniques. By measuring the ancilla qudit at 30 discrete time stamps, we obtain the time series ${{{\mathcal{A}}}}(t)={{{\rm{Tr}}}}({e}^{-i{\hat{H}}_{NH}t})/d$ for different *g* values, which captures oscillations associated with eigenenergies along the principal diagonal of the Jordan matrix. The Fourier transformed spectra are obtained and reported in Fig. 4g, which displays three distinct regimes: in PT exact phase (*g* = 0.4, ${\widetilde{{{{\mathcal{A}}}}}}_{s}$), at exceptional point (*g* = 0.5, ${\widetilde{{{{\mathcal{A}}}}}}_{e}$) and in PT broken phase (*g* = 0.6, ${\widetilde{{{{\mathcal{A}}}}}}_{b}$). The Gaussian peaks in all spectra are centered at the real parts of eigenenergies, with QCS clearly capturing their coalescence at the exceptional point. Note that the negative amplitudes and oscillating behavior in the PT-broken phase spectrum directly result from non-vanishing imaginary part of eigenenergies, serving as clear signatures of phase transition.

### Periodically driven quantum systems

The drive-induced non-equilibrium dynamics can bring out new phenomena not captured by the original system, such as Floquet topogical phases and Floquet topological insulator with quasi-eigenenergies band structures^26,47^. Traditional approaches for static eigen-problems fail to resolve these emergent topological features. Previous methods^22–24,32^ face challenges in resolving fine sub-band energy levels, as they cannot completely eliminate spectral lines arising from excitations (see Supplementary Note 2). In contrast, QCS overcomes this limitation, enabling the detection of subtle spectral fingerprints of topological dynamical behavior.

We investigate nuclear quadrupole resonance of a spin-$\frac{3}{2}$ system subjected to a periodically-driven magnetic field **B**(*t*) with angular frequency Ω:

5 $$\hat{H}(t)={({{{\bf{B}}}}(t)\cdot \hat{{{{\bf{S}}}}})}^{2},\quad {{{\bf{B}}}}(t)=B(\sin \theta \cos \Omega t,\sin \theta \sin \Omega t,\cos \theta ),$$

to demonstrate the quasi-eigenenergy spectroscopy and further estimate the topological holonomy for such dynamics^48–50^. The magnitude of the magnetic field is set as *B* = 2 in our experiment. We first consider the case of applying a static magnetic field, for which the Hamiltonian yields four eigenstates grouped into two doubly degenerate manifolds with eigenenergies *B*^2^/4 and 9*B*^2^/4, indicated by the two black dotted lines in Figs. 4h, 4i. For these two degenerate eigen-spaces, adiabatic transport along a closed loop in the parameter space would formulate a topological holonomy, characterized by the Wilczek-Zee (WZ) phase factor acting on the initial eigenstates^51^:

6 $${\Phi }_{{{{\rm{WZ}}}},ab}={{{\mathcal{P}}}}\exp \left[i\oint {{{{\bf{A}}}}}_{\mu,ab}{{{\rm{d}}}}{x}^{\mu }\right],$$

where ${{{\mathcal{P}}}}$ is path-ordering operator, and $\left.| {\phi }_{a}(x)\right\rangle$ is the eigenstate in a certain degenerate eigen-space and **A**_*μ*,*ab*_ = *i*〈*ϕ*_*a*_(*x*)∣∂_*μ*_∣*ϕ*_*b*_(*x*)〉 is the gauge field revealing the geometric structure of the parameter space (**B** in this case). Generally, *Φ*_WZ,*ab*_ forms a two-dimensional unitary transformation in the doubly degenerate eigen-space, corresponding to an SU(2) holonomy.

To approximate adiabatic evolution and accurately reveal topological phenomena, we choose the driving frequency Ω as 0.5. The initial state ${\left\vert \psi \right\rangle }_{T}$ is prepared as ${e}^{-i\theta {\hat{S}}_{y}}{(0,1,0,0)}^{T}$ for the lower degenerate eigenspace and ${e}^{-i\theta {\hat{S}}_{y}}{(0,0,0,1)}^{T}$ for the upper eigenspace, where ${\hat{S}}_{y}$ is the four-dimensional representation of the *y*-component spin operator $\hat{S}$. We select the Gaussian window width *τ* of 15 to ensure sufficient spectral resolution, with 150 discrete time steps for large spectral range. On the quantum chip, the time-ordered time-evolution operator $\hat{U}(t)={{{\mathcal{T}}}}{e}^{-i\int_{0}^{t}{({{{\bf{B}}}}(t^{\prime} )\cdot \hat{S})}^{2}{{\mbox{d}}}t^{\prime} }$ is implemented via the generalised Hadamard test circuits in Fig. 1c. Using QCS, we measured the quantum auto-correlation function ${{{{\mathcal{C}}}}}_{\hat{I},\psi }(t)$ and Fourier-transformed eigen-spectra ${\widetilde{{{{\mathcal{C}}}}}}_{\hat{I},\psi }(\omega )$ for two cases: *θ* = *π*/2 (Fig. 4h) and *θ* = *π*/4 (Fig. 4i), where *θ* is the zenith angle of the magnetic field on the sphere.

Experimental results reveal the comprehensive Floquet spectrum and topological signatures in this periodically driven systems. First, the number of quasi-energy states (represented by blue Gaussian peaks) exceeds the Hamiltonian’s dimension, which is a characteristic feature of periodically driven systems as predicted by Floquet theory^26^. Figure 4h and i show the Floquet-Bloch band structures (colored bands), where the Ω-periodic replica bands are fully resolved. Our results show excellent agreement with theoretical Floquet spectra. Second, the quasi-energy spectra show significant differences between Fig. 4h and i, particularly pronounced in the first Floquet-Brillouin zone (red-shaded bands), which contains the eigenenergies of the static system. That corresponds to different topological holonomy. When the gauge field accumulates a scalar phase *γ* during one driving period for states in the degenerate subspace, we observe the expected energy shift Δ*E* = *γ*Ω/(2*π*) relative to the static system. In Fig. 4h, evolution along the equator (e.g., *θ* = *π*/2) creates single shifted Gaussian peaks in both lower and upper level bands, signaling the reduction of SU(2) holonomy to the U(1) Berry phases for each eigenstate^52^. This originates from diagonal WZ phase factors, with measured energy shifts of 0.228 for both levels correspond to a topological phase of 0.912*π*, approaching the predicted *π*-phase from adiabatic evolution. Notably, evolution along the polar circle at *θ* = *π*/4 as shown in Fig. 4i reveals distinct behaviour: The double-peak structure emerges in the lower-level first band, implying genuine SU(2) holonomy. From this double-peak splitting energy Δ*E*_split_, we estimate the gauge-invariant Wilson loop for the SU(2) holonomy as ${{{\mathcal{W}}}}=Tr[{\Phi }_{WZ}]=2\cos (\pi \Delta {E}_{{{{\rm{split}}}}}/\Omega )=-0.499$, close to the theoretical prediction of  − 0.504. But for the upper level, degenerate eigenstates still acquire U(1) Berry phase as no emergent sub-band energy splitting. The slight deviation of estimated topological values stems from non-adiabatic coupling between the lower and upper levels. Thus, QCS enables precise computation of Floquet spectra, providing a powerful tool to investigate exotic topological phases and non-equilibrium phenomena in complex quantum systems. For more general time-dependent Hamiltonians, where the spectrum itself evolves in time, QCS can seamlessly incorporate time-frequency analysis (such as the short-time Fourier transform) to track these temporal variations. A simulation result is provided in Supplementary Fig. 8 to demonstrate this capability. This integrated capability further confirms the generality of the QCS in addressing non-stationary quantum dynamics.

## Discussion

We have demonstrated a generalised quantum computational spectroscopy that can efficiently compute spectral properties through quantum auto-correlation function measurements, and experimentally validated the methodology at a proof-of-principle level on a programmable silicon-photonic quantum chip that implements arbitrarily controlled unitaries with high fidelity. By leveraging phase orthogonality in quantum dynamics, the method resolves eigenstates gapped at the real part of eigenenergies, enables eigenstate tomography, detects exceptional points through PT phase transitions, and estimates topological holonomy from Floquet spectra. Numerical simulations further validate the method’s capability to resolve spectral features of general quantum dynamics under realistic noise conditions. Future extensions could integrate classical shadow techniques^24^ to broaden applicability, employ Laplace transforms^53^ to resolve imaginary part of eigenenergies in non-Hermitian systems, and incorporate wavelet analysis^54^ for multiscale and time-frequency analysis of non-equilibrium dynamics. Our approach provides a useful tool to study quantum many-body systems, open quantum systems and non-equilibrium systems. The synergy of quantum control and classical spectral analysis presented here could also inspire further refinements for quantum simulation.

## Methods

### Fourier expansion of general quantum dynamics

The analysis of the QCS for general quantum systems is based on the universal Fourier series expansion of the time-evolved initial state: $\hat{U}(t)\left\vert \psi \right\rangle={\sum }_{n}{c}_{n}{e}^{-i{E}_{n}t}\left\vert {u}_{n}\right\rangle$ with quasi-eigenenergies *E*_*n*_ and quasi-eigenstates *u*_*n*_. We here show the physical meanings of these quantities. The quantum dynamics is generally governed by the Schrödinger equation with Hamiltonian $\hat{H}(t)$ (not necessarily Herimtian):

7 $$i\frac{\partial }{\partial t}\left\vert \psi (t)\right\rangle=\hat{H}(t)\left\vert \psi (t)\right\rangle$$

which can be Fourier transformed:

8 $$\int{{{\rm{d}}}}t{e}^{i\omega t}i\frac{\partial }{\partial t}\left\vert \psi (t)\right\rangle=\int{{{\rm{d}}}}t{e}^{i\omega t}\hat{H}(t)\left\vert \psi (t)\right\rangle$$

suppose that the wave function vanishes at infinity, the left hand side can be integrated by parts:

9 $$-\int{{{\rm{d}}}}t(i\omega ){e}^{i\omega t}i\left\vert \psi (t)\right\rangle=\int{{{\rm{d}}}}t{e}^{i\omega t}\hat{H}(t)\left\vert \psi (t)\right\rangle$$

which can be finally simplified as:

10 $$\omega \left\vert u(\omega )\right\rangle=\int\frac{{{{\rm{d}}}}\omega ^{\prime} }{2\pi }\widetilde{H}(\omega -\omega ^{\prime} )\left\vert u(\omega ^{\prime} )\right\rangle$$

where $\left\vert u(\omega )\right\rangle=\int{{{\rm{d}}}}t{e}^{i\omega t}\left\vert \psi (t)\right\rangle$ is the Fourier transformed state vector for the time-evolved state, and $\widetilde{H}(\omega )=\int\,{\mbox{d}}\,t{e}^{i\omega t}\hat{H}(t)$ is the Fourier components of the time-dependent Hamiltonian.

For most concerned quantum systems, the Fourier components of the Hamiltonian and its generated spectrum are discretised. As such, we consider discretised Fourier decompositions: $\hat{H}(t)={\sum }_{m}{e}^{-i{\omega }_{m}t}{\widetilde{H}}^{(m)}$ ($m\in {\mathbb{Z}}$) and $\left\vert \psi (t)\right\rangle={\sum }_{n}{e}^{-i{E}_{n}t}\left\vert u({E}_{n})\right\rangle$ ($m\in {\mathbb{Z}}$). Note that $\left\vert u({E}_{n})\right\rangle$ is just the Fourier coefficients vector of $\left\vert \psi (t)\right\rangle$ and not necessarily orthonormal to each other. Then we can obtain the following continuous Fourier transformation: $\widetilde{H}(\omega )={\sum }_{m}2\pi \delta (\omega -{\omega }_{m}){\widetilde{H}}^{(m)}$ and $\left\vert u(\omega )\right\rangle={\sum }_{n}2\pi \delta (\omega -{E}_{n})\left\vert u({E}_{n})\right\rangle$. Substituting this into Eq.(10), we can derive:

11 $$\omega {\sum}_{n}2\pi \delta (\omega -{E}_{n})\left\vert u({E}_{n})\right\rangle={\sum}_{m,n^{\prime} }2\pi \delta (\omega -{E}_{n^{\prime} }-{\omega }_{m}){\widetilde{H}}^{(m)}\left\vert u({E}_{n^{\prime} })\right\rangle$$

For the gapped real *E*_*n*_, we can integrate the above equation through the interval (*E*_*n*_ − *ϵ*, *E*_*n*_ + *ϵ*) with the infinitesimal *ϵ*, which generates:

12 $${E}_{n}\left\vert u({E}_{n})\right\rangle={\widetilde{H}}^{(0)}\left\vert u({E}_{n})\right\rangle+{\sum }_{m\ne 0}{\widetilde{H}}^{(m)}\left\vert u({E}_{n}-{\omega }_{m})\right\rangle$$

Additionally, for any ${E}_{n^{\prime} }={E}_{n}-{\sum }_{l}{k}_{l}{\omega }_{l}$ with positive integers *k*_*l*_, which is the modulated energy associated with *E*_*n*_, we can derive that:

13 $${E}_{n}\left\vert u({E}_{n^{\prime} })\right\rangle=\left({\widetilde{H}}^{(0)}+{\sum}_{l}{k}_{l}{\omega }_{l}\right)\left\vert u({E}_{n^{\prime} })\right\rangle+{\sum }_{m\ne 0}{\widetilde{H}}^{(m)}\left\vert u({E}_{n^{\prime} }-{\omega }_{m})\right\rangle$$

Stacking up all equations associated with *E*_*n*_ (for any ${E}_{n^{\prime} }$), we arrive at an eigen-problem in the extended Hilbert space:

14 $${{{\mathcal{H}}}}{{{{\mathcal{U}}}}}_{n}={E}_{n}{{{{\mathcal{U}}}}}_{n}$$

15 $${{{\mathcal{H}}}}=\left(\begin{array}{ccccc}\ddots &&&&\\ &{\hat{H}}^{(0)}+{\sum }_{l}{k}_{l}{\omega }_{l}-{\omega }_{p}&{\widetilde{H}}^{(p)}&{\widetilde{H}}^{(p^{\prime} )}&\\ &{\hat{H}}^{(-p)}&{\hat{H}}^{(0)}+{\sum }_{l}{k}_{l}{\omega }_{l}&{\hat{H}}^{(p)}&\\ &{\hat{H}}^{(-p^{\prime} )}&{\hat{H}}^{(-p)}&{\hat{H}}^{(0)}+{\sum }_{l}{k}_{l}{\omega }_{l}+{\omega }_{p}&\\ &&&&\ddots \end{array}\right)$$

16 $${{{{\mathcal{U}}}}}_{n}=\left(\begin{array}{c}\vdots \\ \left\vert u({E}_{n^{\prime} }+{\omega }_{p})\right\rangle \\ \left\vert u({E}_{n^{\prime} })\right\rangle \\ \left\vert u({E}_{n^{\prime} }-{\omega }_{p})\right\rangle \\ \vdots \end{array}\right)$$

where *p* is a integer, ${\widetilde{H}}^{(-p)}$ denotes the Fourier component of Hamiltonians corresponding to ${e}^{i{\omega }_{p}t}$ and ${\widetilde{H}}^{(p^{\prime} )}$ (${\widetilde{H}}^{(-p^{\prime} )}$) represents the component with the frequency ${\omega }_{p^{\prime} }=2{\omega }_{p}$ (${\omega }_{p^{\prime} }=-2{\omega }_{p}$). We only show the interactions between three Fourier components, but in general the eigen-problem can be infinite-dimensional, and we usually truncate those components in high-frequency for good enough approximation.

The *E*_*n*_ from solving the Eq. (14) together with its modulated energy ${E}_{n^{\prime} }={E}_{n}-{\sum }_{l}{k}_{l}{\omega }_{l}$ constructs the full spectrum of general quantum systems, all of which we call quasi-eigenenergies denoted in the same form *E*_*n*_ (including all ${E}_{n^{\prime} }$). Associated with each quasi-eigenenergy *E*_*n*_, the solved $\left\vert {u}_{n}\right\rangle=\left\vert u({E}_{n})\right\rangle$ is named as quasi-eigenstate and obviously not necessarily orthonormal to each other. Hence, any time-evolved state can be expanded by these Fourier components $\hat{U}(t)\left\vert \psi \right\rangle={\sum }_{n}{c}_{n}{e}^{-i{E}_{n}t}\left\vert {u}_{n}\right\rangle$, and for the unit-norm condition of quantum states, we induce additional coefficients *c*_*n*_ to normalise the time-evolved state.

For the time-independent Hamiltonian, the Eq. (12) degrades into the traditional eigen-problem in the stationary Schrödinger equation

17 $${E}_{n}\left\vert u({E}_{n})\right\rangle={\widetilde{H}}^{(0)}\left\vert u({E}_{n})\right\rangle=\hat{H}\left\vert u({E}_{n})\right\rangle$$

with eigenerengy *E*_*n*_ and eigenstate $\left\vert u({E}_{n})\right\rangle$. For the periodically-driven Hamiltonian with driven frequency Ω, *ω*_*m*_ = *m*Ω ($m\in {\mathbb{Z}}$) and *E*_*n*_ is associated with ${E}_{n^{\prime} }={E}_{n}-m\Omega$, which forms the repetitive Floquet-Bloch bands separated by Ω. The distinct eigenvalues of Eq. (14) generate the sub-band spectrum in a single band, as shown in Fig. 4i.

### Query depth

QCS scales as ${{{\mathcal{O}}}}[{(\sqrt{2{{{\rm{ln}}}}(1/{\epsilon }_{1})}R(\hat{H})/\Delta {E}_{\min })}^{2}/{\epsilon }_{2}^{2}]$. The quantity $\sqrt{2{{{\rm{ln}}}}(1/{\epsilon }_{1})}R(\hat{H})/\Delta {E}_{\min }$ represents the required points in the discrete-time Fourier transformation for the required integral truncation error *ϵ*_1_. Since we consider the query unitary as the single-step time-evolution unitary, this quantity is proportional to the query depth. Then we need translate the Fourier integral truncation error to the energy estimation error. For ideal case, the Gaussian peak in the spectrum is $f(\omega )={\zeta }^{2}\exp (-\frac{1}{2}{\tau }^{2}{(\omega -{E}_{n})}^{2})$. Then we can obtain the dependence of energy error *δω* on the function error *δf* (i.e. Fourier integral error) near the peak: ∣*δω*∣^2^ ≈ 2∣*δf*∣/(*ζ*^2^*τ*^2^). The quantity can be translated to $\sqrt{2{{{\rm{ln}}}}(2/({\epsilon }^{2}{\zeta }^{2}{\tau }^{2}))}R(\hat{H})/\Delta {E}_{\min }$. With relation of the rescaled energy gap $\Delta=\Delta {E}_{\min }/R(\hat{H})$ and *τ* ~ 1/Δ, we can obtain the query depth of QCS scales as ${{{\mathcal{O}}}}({\Delta }^{-1}\sqrt{\log ({\epsilon }^{-1}{\zeta }^{-1}\Delta )})$.

### Query complexity for Hermitian eigenenergy estimation

We first outline this task for QCS. The estimation of ground-state (or eigenstate) energy of time-independent Hermitian Hamiltonians only involves the identity-operator QACF, and it would degrade into the Hadamard test estimator ${{{{\mathcal{C}}}}}_{\hat{I},\psi }=\int{{{\rm{d}}}}\eta {{{\mathcal{G}}}}(\eta,\tau )\langle \psi | {e}^{i\hat{H}\eta }{e}^{-i\hat{H}(\eta+t)}| \psi \rangle=\langle \psi | {e}^{-i\hat{H}t}| \psi \rangle$. This estimator together with the discrete-time Fourier transformation contributes the query complexity as ${{{\mathcal{O}}}}(\sqrt{2ln(1/{\epsilon }_{1})}\tau R(\hat{H})/{\epsilon }_{2}^{2})$. Note that the square dependence is dropped, but it is not the best query complexity of QCS. We can choose constant *ϵ*_1_ and *ϵ*_2_ that contribute a constant spectral function error *δf*. The energy estimation error can be represented by $\delta \omega \approx 2\sqrt{\delta f}/(\zeta \tau )$, hence *τ* can scale as ${{{\mathcal{O}}}}({\epsilon }^{-1}{\zeta }^{-1})$ to achieve the target estimation error *δω* < *ϵ*. In this case, when $R(\hat{H})$ is determined, the query complexity of QCS efficiently scales as ${{{\mathcal{O}}}}({\epsilon }^{-1}{\zeta }^{-1})$. Note that this best query complexity comes at the expense of increasing query depth for QCS, since *τ* changes the scaling behaviour from ${{{\mathcal{O}}}}({\Delta }^{-1})$ to ${{{\mathcal{O}}}}({\epsilon }^{-1})$.

### Device fabrication

Integrated silicon-photonic quantum chips implementing the generalized Hadamard test were fabricated using standard CMOS processes. The waveguide-based quantum circuits (Fig. 2a) were patterned on an 8-inch silicon-on-insulator (SOI) wafer through 248 nm deep ultraviolet (DUV) photolithography and inductively coupled plasma (ICP) etching. Following waveguide fabrication, a 1 μm-thick silicon dioxide layer was deposited via plasma-enhanced chemical vapor deposition (PECVD). A 50 nm-thick titanium nitride layer was then deposited atop the waveguides to serve as thermo-optic phase shifters. The silicon waveguides feature a cross-section of 450 nm × 220 nm. Photon-pair sources with 1.5 cm-long waveguide sections were designed to generate sufficiently bright entangled photons. Balanced beamsplitters were implemented using multimode interferometers measuring 27 μm × 2.8 μm. The chip is controlled by a multi-channel digital-to-analog converter driver with a maximum output voltage of 12 V.

### Experimental setup

In our experiment, a tunable continuous-wave laser centered at 1550.12 nm pumped the waveguide photon sources after amplification to 200 mW using an erbium-doped fiber amplifier (EDFA). Photon pairs were generated via spontaneous four-wave mixing (SFWM) in integrated sources and subsequently separated by on-chip asymmetric Mach-Zehnder interferometers (AMZIs), with signal and idler photons at 1545.32 nm and 1554.94 nm, respectively. The photons were coupled off-chip via grating couplers and detected by fiber-coupled superconducting nanowire single-photon detectors (SNSPDs, average efficiency: 85%). Coincidence counts were recorded using a multichannel time-interval analyzer. Photon rates varied with the prepared quantum states and measurement bases. For each generalized Hadamard test expectation value estimation, we used 5 s integration time in projective measurements to achieve ~1000 two-photon coincidences, ensuring sufficiently small shot noise.

## Supplementary information

Supplementary Information

Transparent Peer Review file

## Data Availability

The data that support the findings of this study are available from the corresponding author upon request.

## Code availability

The numerical simulation of noisy quantum circuits is performed by MindSpore Quantum^42^, the codes of which are available on GitHub https://github.com/Celesoar/MindQuantum-for-UQCS.git and cited in the reference list^43^.
