# Supplementary material for: Generalised quantum computational spectroscopy on a quantum chip
Source: Nat Commun. 2026 Jul 11;17:8589. doi: 10.1038/s41467-026-74936-7 (PMC13486694; doi:10.1038/s41467-026-74936-7)
Supplement: Supplementary file 1 [file 41467_2026_74936_MOESM1_ESM.pdf]

Supplementary Information: Generalised Quantum Computational Spectroscopy on a Quantum Chip

Chonghao Zhai[1,†,⋆], Jinzhao Sun[2,3,†], Jieshan Huang[1], Jun Mao[1], Hongchang Bao[1], Siyuan Zhang[1], Vlatko Vedral[2], Xiao Yuan[4,⋆], Jianwei Wang[1,5,6,7,⋆]

[1] *State Key Laboratory for Mesoscopic Physics, School of Physics, Peking University, Beijing, 100871, China*
[2] *Clarendon Laboratory, University of Oxford, Parks Road, Oxford OX1 3PU, United Kingdom*
[3] *School of Physical and Chemical Sciences, Queen Mary University of London, London, E1 4NS, UK*
[4] *Center on Frontiers of Computing Studies, School of Computer Science, Peking University, Beijing 100871, China*
[5] *Frontiers Science Center for Nano-optoelectronics & Collaborative Innovation Center of Quantum Matter, Peking University, Beijing, 100871, China*
[6] *Collaborative Innovation Center of Extreme Optics, Shanxi University, Taiyuan, Shanxi 030006, China*
[7] *Hefei National Laboratory, Hefei 230088, China*
† *These authors contributed equally to this work.* ⋆ *emails: chonghao.zhai@pku.edu.cn, xiaoyuan@pku.edu.cn, jww@pku.edu.cn*

Supplementary Note 1 – Quantum computational spectroscopy

Our quantum computational spectroscopy is based on the quantum auto-correlation function that utilise the phase orthogonality emerging from quantum dynamics to extract the oscillating signal of quasi-eigenenergies. Afterwards, the Fourier transformation could translate such time-series to the frequency domain, in which we can obtain several properties of quasi-eigenstates. We here take the time-independent Hermitian Hamiltonian as an example to show the implementation of quantum computational spectroscopy. Then we discuss how to reduce the uncorrelated errors in our experiments by carrying out the singular spectrum renormalisation of sampled time-series.

1.1 Windowed quantum auto-correlation function and Fourier transformation

The quantum auto-correlation function is defined as the auto-correlation on the infinite time range of the time-evolution operator $\hat{U}(t) = e^{-i\hat{H}t}$:

$$\mathcal{C}_{\hat{O},\psi}(t) = \int \mathrm{d}\eta \langle\psi|e^{i\hat{H}\eta}\hat{O}e^{-i\hat{H}(\eta+t)}|\psi\rangle = \sum_{n,m} c_n^* c_m e^{-iE_m t}\langle\phi_n|\hat{O}|\phi_m\rangle \int \mathrm{d}\eta e^{i(E_n-E_m)\eta} \tag{1}$$

where E_n is the n-th eigenenergy with the eigenstate $|\phi_n\rangle$. The integral of the phase factor is obviously not convergent, and we uitilise the Dirac-δ function to define this integral: $\int d\eta e^{i(E_m-E_n)\eta} = 2\pi\delta(E_m - E_n)$, which formally shows the phase orthogonality from the dynamical phase. To avoid the singularity in the Dirac-δ function, we add a Gaussian window $\mathcal{G}(\eta,\tau) = \frac{1}{\sqrt{2\pi}\tau}e^{-\frac{\eta^2}{2\tau^2}}$ to the integral:

$$\mathcal{C}_{\hat{O},\psi}(t) = \int \frac{\mathrm{d}\eta}{\sqrt{2\pi}\tau} e^{-\frac{\eta^2}{2\tau^2}}\langle\psi|e^{i\hat{H}\eta}\hat{O}e^{-i\hat{H}(\eta+t)}|\psi\rangle = \sum_{n,m} c_n^* c_m e^{-\frac{1}{2}(E_m-E_n)^2\tau^2}e^{-iE_m t}\langle\phi_n|\hat{O}|\phi_m\rangle = \sum_n (1+\mathcal{O}[e^{-\frac{1}{2}\Delta_n^2\tau^2}])|c_n|^2 e^{-iE_n t}\langle\phi_n|\hat{O}|\phi_n\rangle \tag{2}$$

where $\Delta_n = \min_{m,m\neq n}|E_m - E_n|$ denotes the minimum energy gap of the n-th eigenenergy. The windowed quantum auto-correlation function forms a weak phase orthogonality for the gapped eigenstate, where the crosstalk of phase oscillating from other eigenenergies is suppressed exponentially (dependent on the minimum energy gap Δ_n and the width τ of Gaussian window: $\mathcal{O}[e^{-\frac{1}{2}\Delta_n^2\tau^2}]$). In our experiment, we take a large width of Gaussian window to make the crosstalk ignorable, then the quantum auto-correlation function can be approximated as: $\mathcal{C}_{\hat{O},\psi}(t) = \sum_n |c_n|^2 e^{-iE_n t}\langle\phi_n|\hat{O}|\phi_n\rangle$. To investigate the eigen-spectrum, we apply the Fourier transformation on the quantum auto-correlation function and a Gaussian window $\mathcal{G}(t,\tau) = \frac{1}{\sqrt{2\pi}\tau}e^{-\frac{t^2}{2\tau^2}}$ is also added to the integral:

$$\widetilde{\mathcal{C}}_{\hat{O},\psi}(\omega) = \int \frac{\mathrm{d}t}{\sqrt{2\pi}\tau} e^{-\frac{t^2}{2\tau^2}} e^{i\omega t}\mathcal{C}_{\hat{O},\psi}(t) = \sum_n |c_n|^2 e^{-\frac{1}{2}\tau^2(\omega-E_n)^2}\langle\phi_n|\hat{O}|\phi_n\rangle \tag{3}$$

Therefore, each gapped eigenstate corresponds to the Gaussian peak centred at the eigenenergy E_n with the amplitude $|c_n|^2\langle\phi_n|\hat{O}|\phi_n\rangle$ in the transformed spectrum defined by $\widetilde{C}_{\hat{O},\psi}(\omega)$. Especially, the identity observable generates the amplitude $|c_n|^2\langle\phi_n|\phi_n\rangle$. Hence, we can estimate each eigenenergy by fitting the Gaussian peaks in the spectrum and calculate the expectation value of a certain observable under eigenstates by extracting the ratio of two spectra generated by the observable $\hat{O}$ and the identity operator: $\langle\hat{O}\rangle_{\phi_n} = \langle\phi_n|\hat{O}|\phi_n\rangle/\langle\phi_n|\phi_n\rangle = \widetilde{C}_{\hat{O},\psi}(E_n)/\widetilde{C}_{\hat{I},\psi}(E_n)$.

Although the Gaussian window function gives a smooth truncation on the time correlation expectation values $\langle\psi|e^{i\hat{H}\eta}\hat{O}e^{-i\hat{H}(\eta+t)}|\psi\rangle$ and quantum auto-correlation time-series $C_{\hat{O},\psi}(t)$ to remove the singularity of Dirac-δ function, we still need to cut off the infinite integral, whose error can be exponentially small. For the integral cut-off to $[-t_m, t_m]$, the truncation error ϵ_1 can be bounded by:

$$\epsilon_1 \leq \int_{|t|>t_m} dt \frac{1}{\sqrt{2\pi}\tau}e^{-\frac{t^2}{2\tau^2}} = \mathrm{erfc}\left(\frac{t_m}{\sqrt{2}\tau}\right) \leq \sqrt{\frac{2}{\pi}}\frac{\tau}{t_m}\exp(-\frac{1}{2}\frac{t_m^2}{\tau^2}),\ \ t_m \geq 0 \tag{4}$$

The second inequality comes from the continued fraction representation of the complementary error function $\mathrm{erfc}(x) = \frac{e^{-x^2}}{\sqrt{\pi}}(\frac{1}{x+}\frac{1/2}{x+}\frac{1}{x+}\frac{3/2}{x+}...)$. Hence, for the cut-off error less than ϵ_1, we can choose the truncation range as $t_m \geq \tau\sqrt{2\ln(\sqrt{\frac{2}{\pi}}\frac{1}{\epsilon_1})} \sim \tau\sqrt{2\ln(\frac{1}{\epsilon_1})}$, which is efficient as

forming the square-root of logarithmic complexity. For instance, we take $t_m = 4\tau$ to suppress the truncation error below 0.01% for both the calculation of quantum auto-correlation function and Fourier transformation.

1.2 Discretisation and sampling

The integral cut-off is not enough for the practical experiment, and we need to further discretise the sampling time to approximate the quantum auto-correlation function and apply the discrete-time Fourier transformation (DTFT), which can be written as:

$$\mathcal{C}_{\hat{O},\psi}(t) = \int d\eta \frac{1}{\sqrt{2\pi}\tau} e^{-\frac{\eta^2}{2\tau^2}} \langle\psi| e^{i\hat{H}\eta} \hat{O} e^{-i\hat{H}(\eta+t)} |\psi\rangle \xrightarrow{\text{discretise}} \sum_j \frac{1}{f_s} \frac{1}{\sqrt{2\pi}\tau} e^{-\frac{\eta_j^2}{2\tau^2}} \langle\psi| e^{i\hat{H}\eta_j} \hat{O} e^{-i\hat{H}(\eta_j+t)} |\psi\rangle \tag{5}$$

$$\widetilde{\mathcal{C}}_{\hat{O},\psi}(\omega) = \int dt \frac{1}{\sqrt{2\pi}\tau} e^{-\frac{t^2}{2\tau^2}} e^{i\omega t} \mathcal{C}_{\hat{O},\psi}(t) \xrightarrow{\text{discretise}} \sum_j \frac{1}{f_s} \frac{1}{\sqrt{2\pi}\tau} e^{-\frac{t_j^2}{2\tau^2}} e^{i\omega t_j} \mathcal{C}_{\hat{O},\psi}(t_j) \tag{6}$$

where we sample the time-points t_j with frequency f_s in time-range $[-t_m, t_m]$. The sampling frequency f_s should be at least larger than the Nyquist frequency $f_s \geq 2f_{\max}$, where $f_{\max}$ is the maximum frequency in the spectrum and can be defined by the spectrum radius of Hamiltonian $R(\hat{H})$, $f_{\max} = R(\hat{H})/(2\pi)$. For large enough t_m, the result of DTFT for a certain eigenstate can be estimated by (the discretised calculation of quantum auto-correlation function can be regarded as a kind of DTFT):

$$\sum_{j=-\infty}^{+\infty} \frac{T_s}{\sqrt{2\pi}\tau} e^{-\frac{(jT_s)^2}{2\tau^2}} e^{-i(E_n-\omega)jT_s} = e^{-\frac{1}{2}\tau^2(E_n-\omega)^2} \vartheta_3\left(\frac{i\pi\tau^2(E_n-\omega)}{T_s}, e^{-\frac{2\pi\tau^2}{T_s^2}}\right) \tag{7}$$

where $T_s = 1/f_s$ is the sampling interval, and $\vartheta_3(u,q) = 1 + 2\sum_{n=1}^{+\infty} q^{n^2}\cos(2nu)$ is the Elliptic-theta function. The discretization error ε near the Gaussian peak can be estimated by the leading-order term of the Elliptic-theta function:

$$\varepsilon \sim e^{-\frac{2\pi\tau^2}{T_s^2}} e^{\frac{\pi\tau^2(E_n-\omega)}{T_s}} \sim e^{-2\pi N_t^2} e^{\pi N_t} \tag{8}$$

where we give two estimations for the error: the number of sampled time points N_t is in the order of $\frac{\tau}{T_s}$ since t_m is chosen as 4τ in our experiment, and the region near the Gaussian peak satisfies $\tau(E_n - \omega) \sim 1$. Hence, the error ε exponentially decreases with the number of sampled time points, forming a good approximation for the windowed Fourier integral.

Considering the statistical error ϵ_2 from the Poissonian noise in the quantum measurement, the total sample complexity is:

$$N_s \sim \frac{1}{\epsilon_2^2}\left(\sqrt{2\ln(\frac{1}{\epsilon_1})}\tau R(\hat{H})\right)^2 \sim \frac{1}{\epsilon_2^2}\left(\sqrt{2\ln(\frac{1}{\epsilon_1})}\frac{R(\hat{H})}{\Delta E_{min}}\right)^2 \tag{9}$$

where ΔE_{min} is the minimum distinguishable energy gap in the spectrum and the overall square comes from the two-dimensional sampling both on the η and t. For local Hamiltonians, the spectrum radius $R(\hat{H})$ grows polynomially with the system size since the local interaction only induces the polynomial growth of bounded operators (like Pauli strings). Hence, for the finitely gapped eigenstates, this sampling complexity grows polynomially with the system size and error requirement, which is efficient for the quantum computational spectroscopy.

1.3 The generalised Hadamard test and circuit realisation

The calculation of quantum auto-correlation function requires weighted linear combination of large amount of unitary operators ($\hat{U}^\dagger(\eta)\hat{O}\hat{U}(\eta+t)$, $\hat{O}$ is a certain Pauli string) at different time points, which is hard to directly implement in the quantum circuits with high-fidelity. Fortunately, the generalised Hadamard test can extract the complex expectation value of unitary operators into the statistical distribution of measurement results on the ancilla qubit under some observables.

The generalised Hadamard test is implemented by the quantum circuit shown in Supplementary Figure 1b, where the controlled time-evolution operator $\hat{U}(t) = e^{-i\hat{H}t}$ is applied, the time-evolution operator $\hat{U}(\eta) = e^{-i\hat{H}\eta}$ is applied on the target qubits and the Hadamard gate $H = \frac{1}{\sqrt{2}}\begin{pmatrix}1 & 1\\ 1 & -1\end{pmatrix}$ and a phase gate $S = \begin{pmatrix}1 & 0\\ 0 & -i\end{pmatrix}$ is applied on the ancilla qubit. The initial states of control and target qubits are prepared as $|0\rangle$ and $|\psi\rangle$, respectively. This quantum circuit works similarly as the Hadamard test (Supplementary Figure 1a). When S-gate is not applied ($a = 0$), the statistical distribution of measurement results is $\Pr(X=0) = \frac{1}{2}(1 + \mathrm{Re}\langle\psi|\hat{U}(\eta)^\dagger\hat{O}\hat{U}(\eta+t)|\psi\rangle), \Pr(X=1) = \frac{1}{2}(1 - \mathrm{Re}\langle\psi|\hat{U}(\eta)^\dagger\hat{O}\hat{U}(\eta+t)|\psi\rangle)$, which can be used to estimate the real part of the expectation value of the unitary. When S-gate is applied ($a = 1$), the distribution is $\Pr(X=0) = \frac{1}{2}(1 + \mathrm{Im}\langle\psi|\hat{U}(\eta)^\dagger\hat{O}\hat{U}(\eta+t)(t)|\psi\rangle), \Pr(X=1) = \frac{1}{2}(1 - \mathrm{Im}\langle\psi|\hat{U}(\eta)^\dagger\hat{O}\hat{U}(\eta+t)(t)|\psi\rangle)$ for the imaginary part. The estimation of the statistical distribution is done by about 1000 single-shot measurements at each η and t for relative low shot noise in the experiment. This circuit model can also be applied for non-Hermitian Hamiltonians by additional preparation circuits and additional target qubits simulating the environment (see Supplementary Note 3).

We realise the generalised Hadamard test by a circuits shown in Supplementary Figure 1c. By simultaneously pumping four sources, a four-dimensional Bell-state $|\Phi\rangle = \frac{1}{2}\sum_{i=0}^{3}|i\rangle_a|i\rangle_L$ is generated, where 'a' denotes ancilla qudit and 'L' denotes layer qudit. To realise the controlled-unitary operation, we need first expand the Hilbert-space of the layer qudit $|i\rangle_L \rightarrow |i\rangle_L|\psi\rangle_T$ ('T' denotes target qudit) by the preparation circuits and apply four U(4) operations on the target qudit. Then, after the coherent compression (CC, projecting the layer qudit onto the state $\frac{1}{2}\sum_i |i\rangle_L$), we can realise a four-dimensional controlled-unitary operation:

$$\frac{1}{2}\sum_i |i\rangle_a|i\rangle_L U_i|\psi\rangle_T \xrightarrow{CC} \frac{1}{2}\sum_i |i\rangle_a U_i|\psi\rangle_T \tag{10}$$

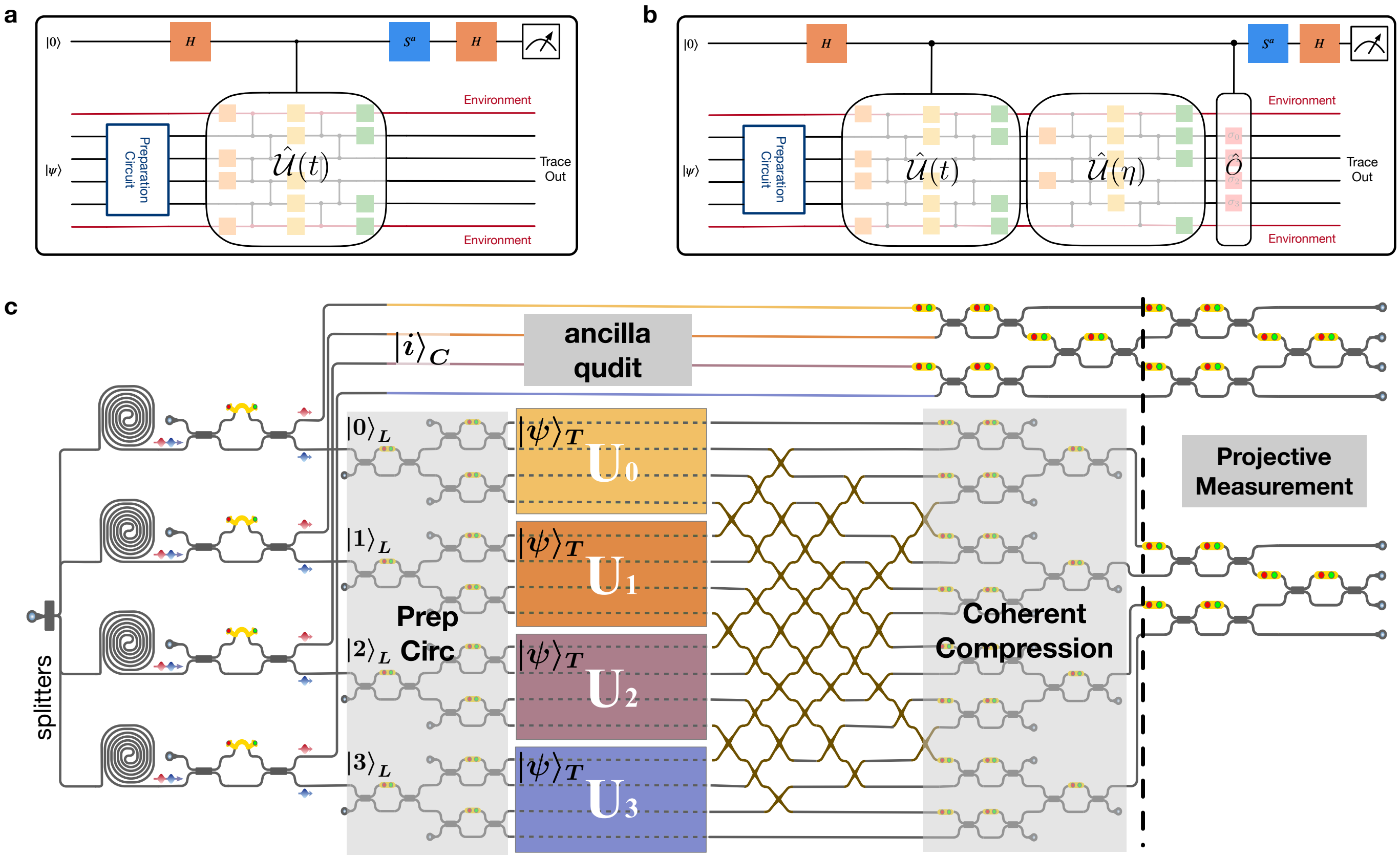

Supplementary Figure 1 a, Circuits model of Hadamard test. b, Generalised Hadamard test. c, Quantum circuits on the silicon photonic chip.

The four-dimensional ancilla qudit could offer duplicated controlled time-evolution in different U(4) quantum optical interferometer networks, which would help mitigate the error from nonuniformity of devices. For instance, when we estimate the real part of a certain unitary U, for ancilla qubit we actually investigate the projection probability onto the state $|+\rangle_a = \frac{1}{\sqrt{2}}(|0\rangle_a + |1\rangle_a)$ and $|-\rangle_a = \frac{1}{\sqrt{2}}(|0\rangle_a - |1\rangle_a)$ with $\Pr(|+\rangle_a) = \frac{1}{2}(1 + \mathrm{Re}\langle\psi|\mathrm{U}|\psi\rangle), \Pr(|-\rangle_a) = \frac{1}{2}(1 - \mathrm{Re}\langle\psi|\mathrm{U}|\psi\rangle)$, which inherits the error of devices. However, when the ancilla register goes to high-dimension, we can apply controlled-U twice in U_2 and U_3 networks, then investigate the projection probability onto the state $|+\rangle_a = \frac{1}{2}(|0\rangle_a + |1\rangle_a + |2\rangle_a + |3\rangle_a)$ and $|-\rangle_a = \frac{1}{2}(|0\rangle_a + |1\rangle_a - |2\rangle_a - |3\rangle_a)$. The projection probability would be $\Pr(|+\rangle_a) = \frac{1}{8}(3 + \mathrm{Re}\langle\psi|\mathrm{U}'^{\dagger}\mathrm{U}|\psi\rangle) + \frac{1}{2}\mathrm{Re}\langle\psi|\frac{\mathrm{U}+\mathrm{U}'}{2}|\psi\rangle, \Pr(|-\rangle_a) = \frac{1}{8}(3 + \mathrm{Re}\langle\psi|\mathrm{U}'^{\dagger}\mathrm{U}|\psi\rangle) - \frac{1}{2}\mathrm{Re}\langle\psi|\frac{\mathrm{U}+\mathrm{U}'}{2}|\psi\rangle$ with slightly different $\mathrm{U}=\mathrm{U}_{ideal} + \delta_1$ and $\mathrm{U}'=\mathrm{U}_{ideal} + \delta_2$ implemented in two networks. Normalising these two probabilities will give the estimation of the real part of expectation value: $(\mathrm{Re}\langle\psi|\mathrm{U}_{ideal}|\psi\rangle + \mathrm{Re}\langle\psi|\frac{\delta_1+\delta_2}{2}|\psi\rangle)/(1 + \frac{1}{4}\mathrm{Re}\langle\psi|\delta_2^{\dagger}\mathrm{U}_{ideal}|\psi\rangle + \frac{1}{4}\mathrm{Re}\langle\psi|\mathrm{U}_{ideal}^{\dagger}\delta_1|\psi\rangle) \sim \mathrm{Re}\langle\psi|\mathrm{U}_{ideal}|\psi\rangle + \mathrm{Re}\langle\psi|\frac{\delta_1+\delta_2}{2} - \mathrm{Re}\langle\psi|\mathrm{U}_{ideal}|\psi\rangle\frac{\delta_1'+\delta_2'}{4}|\psi\rangle)$, using the fact that statistically, the product of the unitary matrix U_{ideal} and the noisy matrix $\delta_{1,2}$ gives the noisy matrix following the same ZMCSCG distribution. Hence, if the elements of δ_1 or δ_2 follows the error distribution $\mathcal{CN}(0, \sigma^2)$, the resulting error of the estimation using four-dimension ancilla qudit follows the error distribution $\mathcal{CN}(0, (\frac{1}{2} + \frac{u^2}{8})\sigma^2)$ with $u = \mathrm{Re}\langle\psi|\mathrm{U}_{ideal}|\psi\rangle$. Since u must be less than 1, the variance of estimation error reduces to at least $\frac{5}{8}$ of the initial error from the quantum devices, forming passive quantum error correction for the linear optics networks[1].

1.4 Singular spectrum renormalisation of sampled quantum auto-correlation function

As the discussion in the main text, through the quantum auto-correlation function, any oscillating noise at frequency f_{noise} distinct from the quasi-eigenstates is strongly suppressed by the Gaussian windowing factor $\exp\left(-\tau^2(E_n - f_{noise})^2/2\right)$ for each quasi-eigenenergy E_n. However, the signal in the quantum auto-correlation function samples could still be submerged by the uncorrelated random noise, which would disturb the extraction of the eigen-spectrum (as shown in Supplementary Figure 2a). The detailed analysis of our silicon-photonic device shows that the gate noise in the experiment is uniform and uncorrelated, along with the photon Poissonian noise in the measurement. These uniform and uncorrelated noises can be efficiently mitigated by the singular spectrum renormalisation of time-series samples[2] as shown in Supplementary Figure 2b. The basic idea of this method is to decompose different time variation modes from the singular value decomposition (SVD) of the trajectory matrix stacked by the time-series samples, which contains three main steps.

For a quantum auto-correlation function time-series $\mathcal{C}_{\hat{I},\psi}(t_j)$ denoted as $\{s_1, s_2, ..., s_N\}$ (shown in Supplementary Figure 3a), we firstly

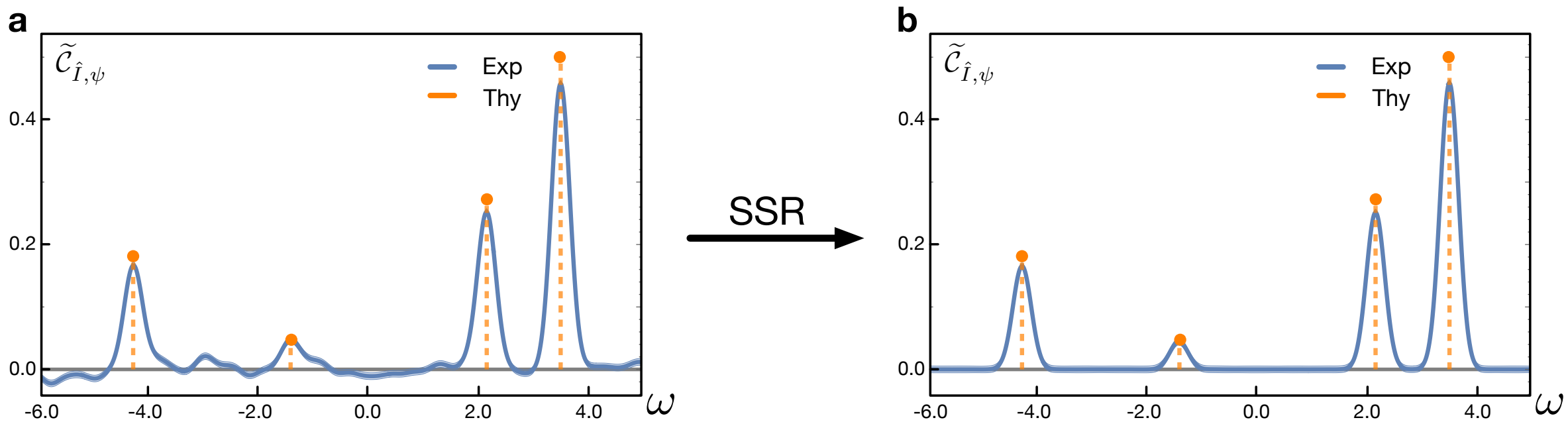

Supplementary Figure 2 The extracted spectrum $\widetilde{C}_{\hat{I},\psi}$ before and after the singular spectrum renormalisation (SSR).

construct the trajectory matrix X by stacking the subseries extracted by the size-L sliding window:

$$X = \begin{pmatrix} s_1 & s_2 & \dots & s_K \\ s_2 & s_3 & \dots & s_{K+1} \\ \dots & \dots & \dots & \dots \\ s_L & s_{L+1} & \dots & s_N \end{pmatrix} \tag{11}$$

where $K = N - L + 1$ is the number of subseries and the trajectory matrix is obviously a Hankel matrix with constant skew diagonals as shown in Supplementary Figure 3b. Secondly, we perform the SVD on the trajectory matrix X:

$$X = U\Lambda V^{\dagger} = \sum_{l} \lambda_l |u_l\rangle\langle v_l| \tag{12}$$

where different singular values λ_l and corresponding left and right singular vectors $|u_l\rangle$ and $|v_l\rangle$ represent the different time variation modes in the time-series samples. The rational analysis should be taken to regroup the singular values to extract the significant modes. Assuming the regrouped trajectory matrix for certain modes is X', the corresponding time-series can be finally reconstructed by the diagonal averaging. As X' is not necessarily a Hankel matrix, the diagonal averaging method take the average of each skew diagonals of X' as a point value in the reconstructed time-series to Hankelized X'. The Fourier transformation of the reconstructed time-series generates a clean spectrum as shown in Supplementary Figure 2.

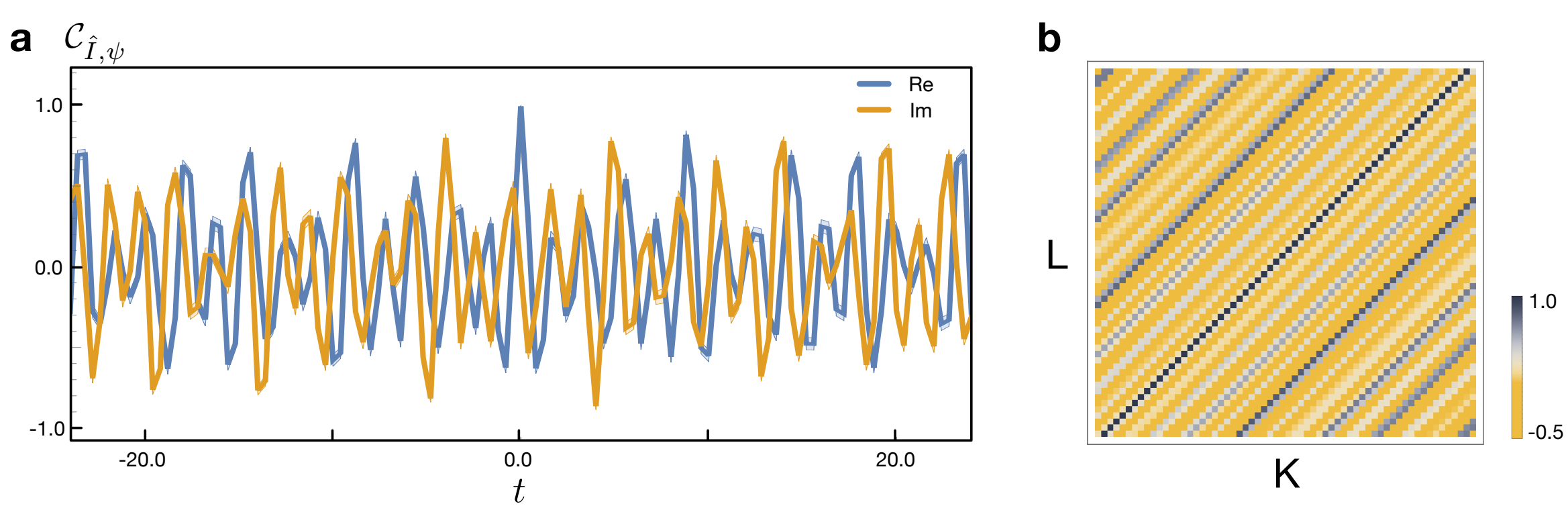

Supplementary Figure 3 The quantum auto-correlation function samples $C_{\hat{I},\psi}(t)$ and its trajectory matrix with dimensin $L \times K$.

This singular spectrum analysis is extremely effective for our case that needs to extract the oscillating components at certain frequencies from the uniform and uncorrelated noise background, which can be seen as the following. Since the time-series are sampled uniformly in the range $[-t_m, t_m]$, we assume $N = 2M + 1$ for M points both in the positive and negative time, $L = \lfloor N/2 \rfloor = M$ and $K = M + 2$. For any oscillating eigenstates at eigenenergy E_n, the trajectory matrix X_n can be written as:

$$X_n = \begin{pmatrix} e^{iE_n \cdot M \cdot T_s} & e^{iE_n \cdot (M-1) \cdot T_s} & \dots & e^{-iE_n \cdot T_s} \\ e^{iE_n \cdot (M-1) \cdot T_s} & e^{iE_n \cdot (M-2) \cdot T_s} & \dots & e^{-iE_n \cdot 2 \cdot T_s} \\ \dots & \dots & \dots & \dots \\ e^{iE_n \cdot T_s} & 1 & \dots & e^{-iE_n \cdot M \cdot T_s} \end{pmatrix} \tag{13}$$

where T_s is the sampling time-interval. Obviously, the rank of this trajectory matrix is one, which can be written as:

$$X_n = \sqrt{L \cdot K} |u_n\rangle\langle v_n| = \sqrt{L \cdot K} \frac{1}{\sqrt{L}} \begin{pmatrix} e^{iE_n \cdot M \cdot T_s} \\ e^{iE_n \cdot (M-1) \cdot T_s} \\ \dots \\ e^{iE_n \cdot T_s} \end{pmatrix} \begin{pmatrix} 1 & e^{-iE_n \cdot T_s} & \dots & e^{-iE_n \cdot (M+1) \cdot T_s} \end{pmatrix} / \sqrt{K} \tag{14}$$

which is a rank-one matrix with the singular value $\sqrt{L \cdot K}$. For the time-series linearly combined by several oscillating eigenstates, the trajectory matrix X can be written as the linear combination of rank-one matrices:

$$X = \sum_n c_n X_n = \sqrt{L \cdot K} \sum_n c_n |u_n\rangle\langle v_n| \tag{15}$$

According to the inequality of the rank of the sum of matrices, $rank(X) \leq N_e$ where N_e denotes the number of concerned oscillating eigenstates. The similarity of two oscillating eigenstates with different frequencies E_n and E_m in the trajectory matrix context can be defined as the inner product of the corresponding left singular vectors:

$$\langle u_n | u_m \rangle = \frac{1}{L} \sum_{j=1}^{L} e^{-i(E_n - E_m) \cdot j \cdot T_s} = \frac{e^{-i(E_n - E_m)T_s}}{L} \frac{1 - e^{-i(E_n - E_m) \cdot L \cdot T_s}}{1 - e^{-i(E_n - E_m)T_s}} \xrightarrow{(E_n - E_m)T_s \to 0} 1 \tag{16}$$

which can also be defined by the right singular vectors. The equation above shows that for a finite energy range, the large sampling frequency (small T_s) would lead to the high similarity of different oscillating eigenstates, resulting in the low-rank trajectory matrix. It means that although the rank of the trajectory matrix is bounded by the number of eigenstates, it would not necessarily increase exponentially with the number of eigenstates. Specially, if $(E_n - E_m)T_s \ll 1$ is satisfied for all $n \neq m$, the trajectory matrix X would approximately be rank-one matrix.

While the oscillating components at certain frequencies form the low-rank trajectory matrix, the uniform and uncorrelated noise background would form full-rank trajectory matrix. The noise background in our experiment can be described by complex-value Gaussian random process: each sample point in time-series $\{g_1, g_2, ..., g_N\}$ follow the independent and identical Zero Mean Circle Symmetric Complex Gaussian (ZMCSCG) distribution $g_i \sim \mathcal{CN}(0, \sigma^2) = \frac{1}{\pi\sigma^2} e^{-|g_i|^2/\sigma^2}$, which can be written as the trajectory matrix G:

$$G = \begin{pmatrix} g_1 & g_2 & \cdots & g_K \\ g_2 & g_3 & \cdots & g_{K+1} \\ \cdots & \cdots & \cdots & \cdots \\ g_L & g_{L+1} & \cdots & g_N \end{pmatrix} \tag{17}$$

Statistically, we can obtain that:

$$\mathbb{E}(GG^\dagger) = K\sigma^2 I_L, \;\; \mathbb{E}(G^\dagger G) = L\sigma^2 I_K, \;\; \mathbb{E}(GX^\dagger) = \mathbb{E}(XG^\dagger) = 0 \tag{18}$$

Hence, the calculation of the SVD on the trajectory matrix contaminated by noise $X + G$ can be statistically considered as the eigenvalue solution of two matrices:

$$\mathbb{E}((X+G)(X+G)^\dagger) = L \cdot K \sum_{n,m} c_n c_m^* |u_n\rangle\langle v_n|v_m\rangle\langle u_m| + K\sigma^2 I_L, \;\; \mathbb{E}((X+G)^\dagger(X+G)) = L \cdot K \sum_{n,m} c_n^* c_m |v_n\rangle\langle u_n|u_m\rangle\langle v_m| + L\sigma^2 I_K \tag{19}$$

The signal-to-noise ratio (SNR) in this context is $\frac{L \cdot K}{K\sigma^2} = \frac{L}{\sigma^2}$ and $\frac{K}{\sigma^2}$, which is typically much larger than one for significant low noise, and the low-rank truncation reserving several largest singular values can efficiently extract the oscillating components from the noise background since most of noise power is in the removed linear space. For the quantum auto-correlation function time-series in our experiment, we usually take the first r largest singular values with r equals to the number of concerned oscillating eigenstates since $(E_n - E_m)T_s \ll 1$ is not satisfied for all eigenstates in consideration of our chosen T_s.

1.5 Limitation of the noise mitigation methods

Although the singular spectrum renormalisation can retrieve the time variation modes from the background noise, the oscillating signal would still be fully submerged by the noise when the noise level exceeds the target eigenstate's projection probability as we show in the Fig. 3f. Here we would explain this limitation by modelling the noise distribution as independent and identical ZMCSCG distribution.

Considering the noise analysis of our quantum chip in Fig. 2, the gate error can be modelled as a statistical noise matrix $\mathcal{N}$, whose elements follow the independent and identical ZMCSCG distribution: $\mathcal{N}_{ij} \sim \mathcal{CN}(0, A) = \frac{1}{\pi A} e^{-|\mathcal{N}_{ij}|^2/A}$. The gate error would be introduced in each discretised sampling time. Although, in our experiment, we directly set the time-evolution operator $e^{-i\hat{H}jT_s}$ for the jth time step on the unitary circuits, the gate error should be considered in each time-evolution step for more general application or the Trotterisation quantum circuits. Then the noisy time-evolution for the jth time step can be modelled as $\prod_{k=1}^{j}(e^{-i\hat{H}T_s} + \mathcal{N}_k)$, where $\mathcal{N}_k$ is the noisy matrix for each time step and independent of each other. Applying this noisy time evolution on the intial state $|\psi\rangle = \sum_l c_l |\phi_l\rangle$, we retain the first-order noisy term and obtain that:

$$\prod_{k=1}^{j}(e^{-i\hat{H}T_s} + \mathcal{N}_k)|\psi\rangle \approx e^{-i\hat{H}jT_s}|\psi\rangle + (\mathcal{N}_1 e^{-i\hat{H}(j-1)T_s} + e^{-i\hat{H}T_s}\mathcal{N}_2 e^{-i\hat{H}(j-2)T_s} + ... + e^{-i\hat{H}(j-1)T_s}\mathcal{N}_j)|\psi\rangle \tag{20}$$

Since the gate we apply on the quantum chip is unitary, the product of the unitary matrix and the noisy matrix $\mathcal{N}_k$ is also a noisy matrix with elements following the independent and identical ZMCSCG distribution. The noisy term can be reduced to the sum of j independent noisy matrices and we denoted it as $\mathcal{N}'$ with each element $\mathcal{N}'_{ij} \sim \mathcal{CN}(0, Aj)$. The Supplementary Equation 20 can be furtherly decomposed into:

$$\sum_l \{c_l e^{-iE_l jT_s} + \langle \phi_l | \mathcal{N}' | \psi \rangle\} |\phi_l\rangle = \sum_l \{c_l e^{-iE_l jT_s} + n_l\} |\phi_l\rangle \tag{21}$$

where $n_l = \langle \phi_l | \mathcal{N}' | \psi \rangle \sim \mathcal{CN}(0, Aj)$ is just a linear superposition with normalised weight of independent and identical ZMCSCG variables, therefore it is also follow the same distribution function. Qualitatively, for a concerned eigenstate, when the standard deviation of n_l exceeds the absolute value of c_l at the jth step, the oscillating signal $e^{-iE_l m T_s}$ for $m \geq j$ would be fully submerged in the noise background. To faithfully extract the spectrum properties for the concerned eigenstate, we would expect j greater than half of total sampling points N, which would cause half reduction to the spectrum resolution. Therefore, we quantify the gate error with the variance of noise at $\frac{N}{2}$th step: $\epsilon_g = A\frac{N}{2}$ and we cannot faithfully extract the spectrum properties when $\sqrt{\epsilon_g} \geq |c_l|$, which equals to $\epsilon_g \geq P_l$ ($P_l = |c_l|^2$ is the projection probability of concerned eigenstates). This qualitative relation between the gate error and projection probability is verified by our simulation shown in Fig. 3f.

Supplementary Note 2 – Comparison of time-correlation-based methods for eigen-property estimation

The defined quantum auto-correlation function (QACF) estimator is the central idea of our QCS algorithm, which is similar to previous time-correlation-based methods[3–6] but has markedly different conceptual nature from them. Since time-correlation-based methods is highly dependent on the characteristics of the time-correlation signal, the advanced application of auto-correlation function in QCS produces rigorously cleaner and more stable eigenspectrum by eliminating the excitation dynamics and suppressing random circuit noise, which is inaccessible for previous ones. It is precisely because of this characteristic that QCS is capable to be applied for a wider range of quantum dynamics systems and shows good agreement with theoretical predictions.

2.1 Elimination of additional cross-term energy peaks from excitations

As emphasized in the main text, the quantum computational spectroscopy methods in Ref. [3–5] measure and transform the time-varying observables $\langle \hat{O}(t) \rangle_\psi$ or their time correlation functions $\langle \hat{A}(t)\hat{B}(t') \rangle_\psi$ with or without ancilla qubits, which could only reveal the cross-term energy peaks from excitations. This is because the estimators of these methods are all composed of Hermitian operators (i.e. $\hat{O}(t) = e^{iHt}\hat{O}e^{-iHt}$). The measured oscillation signal over time only contains the excitation dynamics: $\langle \hat{O}(t) \rangle_\psi = \sum_n \sum_m e^{i(E_n - E_m)t} O_{nm} c_n^* c_m$ and $\langle \hat{A}(t)\hat{B}(t') \rangle_\psi = \sum_{n,m,l} e^{i(E_n - E_m)t} e^{i(E_m - E_l)t'} A_{nm} B_{ml} c_n^* c_l$, where $O_{nm} = \langle n | \hat{O} | m \rangle$, $c_n = \langle n | \psi \rangle$. Hence, this type of time-correlation-based method is difficult to apply to the estimation of eigen-properties.

On the other hand, the Ref. [6] also proposed a time-correlation-based method with a non-Hermitian estimator $\langle \psi | e^{iHt} \hat{O} e^{iHt'} | \psi \rangle$, from which the eigen-properties can be extracted. However, in practice, the windowed Fourier transform should be applied to avoid the infinite integral:

$$\begin{aligned} f(\omega, \omega') &= \int dt \int dt' \mathcal{G}(t,\tau)\mathcal{G}(t',\tau) e^{-i\omega t} e^{-i\omega' t'} \langle \psi | e^{iHt} \hat{O} e^{iHt'} | \psi \rangle \\ &= \sum_n \sum_m e^{-\frac{1}{2}(\omega - E_n)^2\tau^2} e^{-\frac{1}{2}(\omega' - E_m)^2\tau^2} O_{nm} c_n^* c_m \\ &\overset{\omega=\omega'}{\longrightarrow} \sum_n \sum_m e^{-(\omega - \frac{E_n + E_m}{2})^2\tau^2} e^{-\frac{1}{4}(E_n - E_m)^2\tau^2} O_{nm} c_n^* c_m \end{aligned} \tag{22}$$

where $\mathcal{G}(t,\tau) = \exp[-t^2/(2\tau^2)]/(\sqrt{2\pi}\tau)$ and $\omega = \omega'$ is the necessary condition of obtaining the eigen-spectrum. The cross term introduces additional energy peaks centred at $\omega = (E_n + E_m)/2$ in the spectrum of this time-correlation-based method. Although the peak amplitude is suppressed by the coefficient $e^{-\frac{1}{4}(E_n - E_m)^2\tau^2}$, the misidentification of eigenenergy peaks still cannot be avoided when the spectrum is dense ($(E_n - E_m) < 1/\tau$) or the amplitude of some eigenenergy peak is comparatively small. Note that although we can enhance the suppression of cross-terms by increasing τ, that means this method requires higher computational complexity than QCS.

On the contrary, the excitation dynamics (or cross-term energy peaks) is fully eliminated in the practical windowed QACF:

$$C(t) = \int \frac{d\eta}{\sqrt{2\pi}\tau} \mathcal{G}(\eta, \tau) \langle \psi | e^{iH\eta} \hat{O} e^{-iH(\eta + t)} | \psi \rangle = \sum_n \sum_m e^{-\frac{1}{2}(E_n - E_m)^2\tau^2} e^{-iE_m t} O_{nm} c_n^* c_m \tag{23}$$

The oscillation signal of QACF has and only has the contribution from eigenstate evolution dynamics (i.e. $e^{-iE_m t}$), which is more obvious in its windowed Fourier transform:

$$\widetilde{C}(\omega) = \sum_n \sum_m e^{-\frac{1}{2}(\omega - E_m)^2\tau^2} e^{-\frac{1}{2}(E_n - E_m)^2\tau^2} O_{nm} c_n^* c_m \tag{24}$$

The cross term of QACF only contributes to the error in estimation of spectrum intensity, but in no way disturbs the estimation of eigenenergy. For the error from cross terms, the QACF gives a squared stronger suppression coefficient $e^{-\frac{1}{2}(E_n - E_m)^2\tau^2}$ than the time-correlation-based method in Ref. [6] ($e^{-\frac{1}{4}(E_n - E_m)^2\tau^2}$). This advanced properties of QACF is critical to the extension of the QCS to general quantum dynamics, not only give a cleaner eigen-spectrum.

In the general case, the eigen-peak amplitude may be comparable small and the spectrum may be relatively dense ($(E_n - E_m) < 1/\tau$), which would impede the identification of eigenenergies and eigenstates for previous time-correlation-based methods. Explicitly, for the open quantum system, including non-Hermitian Hamiltonians, the evolution dynamics usually experience damping or amplifying (the energy has an imaginary part $E_n + i\lambda_n$). The positive (negative) λ_n leads to a exponentially increasing (decreasing) evolution $e^{\lambda_n t}$. Since the positivity and negativity of λ_n will alter for different eigenstates, the proportions of different eigenstates can vary greatly due to different evolution behaviours (damping or amplifying). The influence of the cross term is especially significant in this case, even exceeding some diagonal terms related to O_{nn}. For this reason, we must use QACF to first completely eliminate the emerging peak at $\frac{E_n + E_m}{2}$ and second largely suppress the cross term with the coefficient $e^{-\frac{1}{2}(E_n - E_m)^2\tau^2}$. Hence, the QACF is necessary for the accurate recognition of eigen peaks in non-Hermitian quantum dynamics.

For the time-dependent Hamiltonian $\hat{H}(t)$, it is difficult for the previous time-correlation-based methods to resolve its quasi-eigen-spectrum, since the spectrum would be more complex and denser. Concretely, the time-dependent Hamiltonian can be decomposed into different frequency components $\hat{H}(t) = \sum_m e^{-i\Omega_p t}\hat{H}^{(p)}$. According to the deduction in Methods, the eigen-spectrum of such Hamiltonian shows the structure like $E_n - m\Omega_p,\ n, m, p \in \mathbb{Z}$, which simultaneously has band (periodic by Ω) and sub-band (distinct E_ns in a single band) structure as shown in the Fig.4(h) in the main text. This complex structure usually makes the spectrum denser, resulting in more hard quasi-eigenenergy identification. The emerging peaks at $\frac{E_n+E_m}{2}$ of the time-correlation-based method in Ref. [6] are significantly disruptive in this case since $(E_n - E_m) < 1/\tau$ weaken the suppression coefficient $e^{-\frac{1}{4}(E_n-E_m)^2\tau^2}$, while the QCS can still give a clean spectrum with no additional energy peaks. More importantly, the accurate identification of quasi-eigenenergy is extremely critical for the recognition of different topological holonomy and the estimation of topological invariants. Concretely, the spectrum fingerprint for the recognition of different topological holonomy relies on the resolution of sub-band structure (i.e. a single peak in a band but shifted indicates the existence of U(1) Berry phase and double-peak splitting in a band implies the transition to SU(2) topological holonomy). Since the driving frequency Ω_p needs to be set low enough ($\Omega_p = 0.5$ in our case) to simulate the adiabatic transport for estimating topological invariant, the width of one band (equals to Ω_p) becomes very narrow, resulting in much denser sub-band structure. Therefore, the additional energy peaks emerging at $\omega = (E_n + E_m)/2$ are disastrous for the recognition of correct topology and topological invariants if we use the previous time-correlation-based methods.

In a word, the advanced feature of eliminating additional cross-term energy peaks is the foundation for QCS to generate cleaner eigen-spectrum and have general applicability to recognize novel spectrum fingerprints in general quantum dynamics.

2.2 Suppression of random circuit noise

The QACF shows novel noise robustness for quantum spectroscopy as well. Since each periodic component of the signal only correlates with its own lagged version and the random noise does not correlate with itself, the QACF can not only ensure no cross term would contribute to the misleading energy peaks, but also identify the periodic signals even in a strong random noise background, which is inaccessible for previous time-correlation-based methods.

The time-correlated circuit noise with a certain frequency range could be suppressed as the cross excitation term shown above (i.e. suppressed by a coefficient $e^{-\frac{1}{2}(E_n-f_{noise})^2\tau^2}$). More importantly, since random circuit noise is time-uncorrelated, it just contributes a rapidly damping signal in QACF. Explicitly, for the circuit noise in our experiment modeled by Gaussian white noise (GWN), its auto-correlation function vanishes except for the zero time:

$$C_{GWN}(t) = \int d\eta\ GWN(\eta) * GWN(\eta + t) = \langle GWN(0) * GWN(t)\rangle = \delta_{t,0} \tag{25}$$

the Fourier transform of which only generates uniform background in the spectrum and can be eliminated easily. The above equation utilizes the statistical averaging to substitute the time averaging, since the GWN is stationary stochastic noise satisfying ergodic conditions. Compared with this feature, the previous time-correlation-based methods could not eliminate the random noise in the time domain, hence the spectrum would be severely contaminated by random noise. The intrinsic suppression of random circuit noise in QACF enables more stable eigen-spectrum with excellent noise resistance.

Supplementary Note 3 – The spectrum analysis of time-independent non-Hermitian Hamiltonian

For a general quantum system, the interaction with the ambient environment can induce complex dynamics, which can be described by the so-called Lindblad master equation for a Markovian environment[7]:

$$\frac{d\hat{\rho}}{dt} = -i[\hat{H}, \hat{\rho}] + \sum_k \left(\hat{\Gamma}_k\hat{\rho}\hat{\Gamma}_k^\dagger - \frac{1}{2}\left(\hat{\Gamma}_k^\dagger\hat{\Gamma}_k\hat{\rho} + \hat{\rho}\hat{\Gamma}_k^\dagger\hat{\Gamma}_k\right)\right) \tag{26}$$

where $\hat{\rho}$ is the density matrix of the quantum system, $\hat{H}$ is a Hermitian Hamiltonian of internal interactions and $\hat{\Gamma}_k$ is jump operators associated with external interactions. The effect of external interactions can be split into two parts: the continuous nonunitary dissipation terms $\hat{\Gamma}_k^\dagger\hat{\Gamma}_k\hat{\rho} + \hat{\rho}\hat{\Gamma}_k^\dagger\hat{\Gamma}_k$ and the quantum jump terms $\hat{\Gamma}_k\hat{\rho}\hat{\Gamma}_k^\dagger$. For an environment with ignorable abrupt stochastic changes (following continuous dynamics), we can neglect the effect of quantum jumps and introduce an effective non-Hermitian Hamiltonian:

$$\hat{H}_{\text{eff}} = \hat{H} - i\sum_k \frac{1}{2}\hat{\Gamma}_k^\dagger\hat{\Gamma}_k \tag{27}$$

The non-Hermitian Hamiltonian generally loses the unitary diagonalisability of the Hermitian Hamiltonian. However, this challenge for the spectrum analysis can be resolved by our quantum computational spectroscopy with the mixed-initial-state Hadamard test.

3.1 Spectral properties of non-Hermitian matrices

Unitary diagonalisation forms a set of orthonormal eigenvectors of the Hermitian matrices. This property holds for a kind of matrices called normal matrices, which satisfy the relation $M^\dagger M = MM^\dagger$. Obviously, the non-Hermitian matrix cannot usually be the normal matrix and the normal non-Hermitian matrix has no difference with the Hermitian matrix except for its complex eigenvalues. Hence, interesting spectral properties of non-Hermitian matrices lie in the non-normal part. For any square matrix $M \in \mathbb{C}^{n\times n}$, we can always reduce it to the Jordan canonical form $M = VJV^{-1}$ by similarity transformation V, where $J = \oplus_{j=1}^{N} \oplus_{\alpha=1}^{G_j} J_{n_{j,\alpha}}(\lambda_j)$ is a block-diagonal matrix with Jordan blocks $J_{n_{j,\alpha}}$

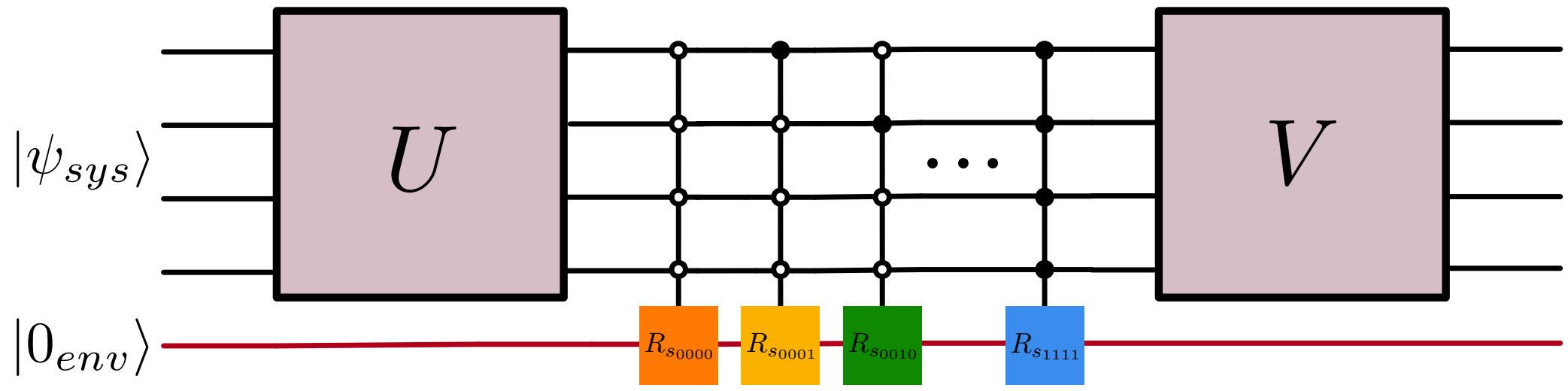

Supplementary Figure 4 Embedded non-Hermitian time-evolution operator into unitary circuits.

on the diagonal (N is the number of eigenvalues, G_j is the geometric multiplicity of λ_j and $n_{j,\alpha}$ is the dimension of the Jordan block) [8]. The Jordan block for eigenvalue λ_j with dimension $n_{j,\alpha}$ can be written as:

$$J_{n_{j,\alpha}}(\lambda_j) = \begin{pmatrix} \lambda_j & 1 & 0 & ... & 0 \\ 0 & \lambda_j & 1 & ... & 0 \\ ... & ... & ... & ... & ... \\ 0 & 0 & 0 & ... & \lambda_j \end{pmatrix}_{n_{j,\alpha} \times n_{j,\alpha}} \tag{28}$$

This general transformation would lead to two key spectral properties of non-Hermitian matrices different from the Hermitian one. The first one is the change of the orthogonality of eigenvectors. For those non-Hermitian matrices with all $n_{j,\alpha}$'s equal to one, the column vectors of V satisfy the eigen-equation of M called the right eigenvectors $M|r_j\rangle = \lambda_j|r_j\rangle$. While the right eigenvectors are not necessarily orthogonal to each other, the row vectors of V^{-1} called the left eigenvectors form the dual eigen-equation $\langle l_j|M = \lambda_j\langle l_j|$ and are orthogonal to the corresponding right eigenvectors $\langle l_k|r_j\rangle = \delta_{k,j}$. The second one is the change of the diagonalizability. The non-Hermitian matrix is not necessarily diagonalizable even with the similarity transformation V, and there could exist some points for a continuous family of non-Hermitian matrices at which the matrix could be only reduced to the Jordan blocks with dimension greater than one, called exceptional points. For the general condition, the function of matrix M can be represented as:

$$f(M) = \sum_{j=1}^{N}[f(\lambda_j)P_j + \sum_{\alpha=1}^{G_j}\sum_{p=1}^{n_{j,\alpha}-1}\frac{1}{p!}f^{(p)}(\lambda_j)A_{j,\alpha}^{p}] \tag{29}$$

where $P_j = \sum_{\alpha=1}^{G_j}\sum_{p=1}^{n_{j,\alpha}}|r_{j,\alpha,p}\rangle\langle l_{j,\alpha,p}|$ denotes the diagonal part in the Jordan canonical form and $A_{j,\alpha} = \sum_{p=1}^{n_{j,\alpha}-1}|r_{j,\alpha,p}\rangle\langle l_{j,\alpha,p+1}|$ represents the off-diagonal part.

These two remarkable characters in the non-Hermitian scenario could bring challenges to the spectrum analysis. The physical eigenstates of non-Hermitian Hamiltonians (the right eigenvectors of non-Hermitian Hamiltonians) could not form an orthogonal complete basis in the Hilbert space, which means we cannot find the eigenstates one-by-one based on the orthogonality. Even worse, when the non-Hermitian system is at exceptional points, the existing off-diagonal components would generate polynomial-form time evolution, which could strongly interfere the extraction of eigen-oscillation and the spectrum.

3.2 Quantum computational spectroscopy for non-Hermitian Hamiltonians

As described above, the normal non-Hermitian matrix has no difference with the Hermitian one except for the existing of imaginary part in the eigenenergies, which would lead to the exponential decay or magnification of the quantum oscillation. This effect would not cause the divergence of the Fourier integral since the Gaussian window function can decay much faster when time goes to large. Hence, the challenge of the spectrum analysis for the non-Hermitian Hamiltonian mainly comes from the non-diagonalisability at exceptional points and the non-orthogonality of the physical eigenstates.

Firstly, we need to embed the non-unitary time-evolution operator into a unitary operator to apply it on the quantum circuits. For simplicity, we use the singular value decomposition (SVD) to embed the non-unitary time-evolution operator into the unitary circuits (shown in the Supplementary Figure 4) such that the Hadamard test can be directly applied to the non-Hermitian scenario. For any non-unitary time-evolution generated by the non-Hermitian Hamiltonian, we can apply the SVD: $e^{-i\hat{H}_{NH}t} = U \cdot diag\{s_{000...0}, s_{000...1}, ...s_{111...1}\} \cdot V$, where U and V are unitary matrices, $s_{z_1z_2z_3...z_n}$ $(z_i = 0, 1)$ are singular values for n-qubit non-Hermitian system and $s_{000...0}$ is the largest singular value. By normalizing the singular values to $diag\{1, s_{000...1}/s_{000...0}, ..., s_{111...1}/s_{000...0}\}$, we can embed the non-unitary time-evolution operator into the unitary circuits by multi-valued controlled rotations with

$$R_{s_{z_1z_2z_3...z_n}} = \begin{pmatrix} s_{z_1z_2z_3...z_n} & -\sqrt{1-s_{z_1z_2z_3...z_n}^2} \\ \sqrt{1-s_{z_1z_2z_3...z_n}^2} & s_{z_1z_2z_3...z_n} \end{pmatrix} \tag{30}$$

where $s_{z_1z_2z_3...z_n}$ has been normalized by $s_{000...0}$. This embedding leads to the relation $\langle 0_{env}|U_{embed}|\psi_{sys}, 0_{env}\rangle = \frac{1}{s_{000...0}}e^{-i\hat{H}_{NH}t}|\psi_{sys}\rangle$. Hence, using the Hadamard test shown in the Supplementary Figure 1a and U_{embed}, we can estimate the expectation value under the intial state $|\psi_{sys}, 0_{env}\rangle$: $\langle\psi_{sys}, 0_{env}|U_{embed}|\psi_{sys}, 0_{env}\rangle$, which is equal to $\frac{1}{s_{000...0}}\langle\psi_{sys}|e^{-i\hat{H}_{NH}t}|\psi_{sys}\rangle$. For the augmented Hadamard

test, the embedding of the non-unitary time-evolutions $e^{i\hat{H}_{NH}\eta}$ and $e^{-i\hat{H}_{NH}(t+\eta)}$ must be applied through different environment qubits, such as $\langle 0_{env1}|U^{(1)}_{embed}|\psi_{sys}, 0_{env1}\rangle = \frac{1}{s^{(1)}_{000...0}} e^{-i\hat{H}_{NH}(t+\eta)}|\psi_{sys}\rangle$ and $\langle 0_{env2}|U^{(2)}_{embed}|\psi_{sys}, 0_{env2}\rangle = \frac{1}{s^{(2)}_{000...0}} e^{-i\hat{H}_{NH}\eta}|\psi_{sys}\rangle$. Therefore, the resulting expectation value $\langle \psi_{sys}, 0_{env1}, 0_{env2}|U^{(2),\dagger}_{embed}\hat{O}U^{(1)}_{embed}|\psi_{sys}, 0_{env1}, 0_{env2}\rangle$ would lead to the same estimation for the non-Hermitian system: $\frac{1}{s^{(1)}_{000...0}s^{(2)}_{000...0}}\langle \psi_{sys}|e^{i\hat{H}^{\dagger}_{NH}\eta}\hat{O}e^{-i\hat{H}_{NH}(t+\eta)}|\psi_{sys}\rangle$.

Secondly, we would deal with the two main challenges in the spectrum analysis of non-Hermitian Hamiltonians. According to Supplementary Equation (29), when the non-Hermitian Hamiltonian cannot be diagonalizable, the generated time-evolution operator contains the polynomial-form time evolution:

$$e^{-i\hat{H}_{NH}t} = \sum_{j=1}^{N}[e^{-i\lambda_j t}P_j + \sum_{\alpha=1}^{G_j}\sum_{p=1}^{n_{j,\alpha}-1}\frac{(-it)^p}{p!}e^{-i\lambda_j t}A^p_{j,\alpha}] \tag{31}$$

in which the polynomial term would ruin the spectrum purely generated by the oscillating term $e^{-i\lambda_j t}$. This would impede estimating the eigenenergies when the non-Hermitian system transitions through the exceptional point. Fortunately, this challenge can be easily overcome in our Quantum computational spectroscopy by initialize the maximally mixed state. The key idea here is that the Hadamard test with maximally mixed initial state would estimate $Tr(e^{-i\hat{H}_{NH}t})$ and $Tr(A^p_{j,\alpha}) = 0$, so that

$$Tr(e^{-i\hat{H}_{NH}t}) = \sum_{j=1}^{N} e^{-i\lambda_j t} * \sum_{\alpha}^{G_j} n_{j,\alpha} = \sum_{j=1}^{N} e^{-i\lambda_j t} n_j \tag{32}$$

where we have extracted the purely oscillating term whether the non-Hermitian system is at the exceptional point or not.

3.3 Pseudo-Hermitian Hamiltonian

Among non-Hermitian Hamiltonians, the pseudo-Hermitian Hamiltonian forms a spectrum most similar to the Hermitian one, although it has non-orthogonal eigenstates and cannot be diagonalized at exceptional points. The η-pseudo-Hermitian Hamiltonian satisfies the relation $\hat{H}^{\dagger}_{PH} = \eta\hat{H}_{PH}\eta^{-1}$, where η is a Hermitian and invertible operator. The spectrum of such Hamiltonians is entirely real or comes into complex conjugate pairs [8]. It is worth noting that the Hamiltonian with $\mathcal{PT}$-symmetry is a special case of the pseudo-Hermitian Hamiltonian, which is actually the $\mathcal{P}$-pseudo-Hermitian Hamiltonian $\hat{H}^{\dagger}_{\mathcal{PT}} = \mathcal{P}\hat{H}_{\mathcal{PT}}\mathcal{P}^{-1}$. When the spectrum of $\mathcal{PT}$-symmetric Hamiltonian changes from entirely real to complex, there will be a phase transition through exceptional points from '$\mathcal{PT}$ exact phase' to '$\mathcal{PT}$ broken phase'.

To demonstrate the application of our Quantum computational spectroscopy on revealing this spectral transition, we choose the toy model of the $\mathcal{PT}$-symmetric-like Hamiltonian in the main text:

$$\hat{H}_{NH} = \begin{pmatrix} \delta - ig & \kappa \\ \kappa & \delta + ig \end{pmatrix} \tag{33}$$

It can be easily verified that the Hamiltonian is Pauli-σ_x-pseudo-Hermitian and σ_x is the parity-like operator in this model. The eigenvalues of this Hamiltonian are $\lambda_{\pm} = \delta \pm \sqrt{\kappa^2 - g^2}$, which are real when $\kappa^2 > g^2$ and come into complex conjugate pairs when $\kappa^2 < g^2$ (the exceptional point is at $\kappa^2 = g^2$). Explicitly, when $g = \kappa$, the Hamiltonian can only be reduced to the Jordan canonical form by similarity transformation:

$$\hat{H}_{NH} = \begin{pmatrix} i & 1/\kappa \\ 1 & 0 \end{pmatrix}\begin{pmatrix} \delta & 1 \\ 0 & \delta \end{pmatrix}\begin{pmatrix} 0 & 1 \\ \kappa & -i\kappa \end{pmatrix} \tag{34}$$

As described in the main text, for a certain $\kappa = 0.5$, when g goes from 0.4 to 0.6, the spectral transition can be revealed by our Quantum computational spectroscopy. Since the initial state is maximally mixed, the windowed Fourier transformation of $Tr(e^{-i\hat{H}_{NH}t})$ would lead to a spectrum $A(\omega) = e^{-\frac{1}{2}\tau^2(\lambda_+ - \omega)^2} + e^{-\frac{1}{2}\tau^2(\lambda_- - \omega)^2}$. Hence, when $g < \kappa$, $\lambda_{\pm}$ form a real and gapped spectrum with two separated Gaussian peaks; when $g > \kappa$, the complex conjugate pairs are degenerate in the real part, but the different imaginary part would cause the oscillation in the spectrum: $A(\omega) = 2e^{-\frac{1}{2}\tau^2[(\delta-\omega)^2-(g^2-\kappa^2)]}\cos(\tau^2\sqrt{g^2-\kappa^2}(\delta-\omega))$; when at the exceptional point $g = \kappa$, the degenerate $\lambda_{\pm}$ just form a single Gaussian peak in the spectrum centered at δ. The behavior of the spectrum totally reveals the $\mathcal{PT}$-symmetric phase transition through the exceptional point.

The other interesting property of the η-pseudo-Hermitian Hamiltonian is that $\lambda_i \neq \lambda^*_j$ implies $\langle r_i|\eta|r_j\rangle = 0$. For the $\mathcal{PT}$-symmetric-like Hamiltonian in Supplementary Equation (33), when $g < \kappa$, the two eigenstates satisfy the relation $\langle r_+|\sigma_x|r_-\rangle = 0$. Since the σ_x is traceless, the expectation value of the observable σ_x would be opposite number for the two eigenstates, which has been verified by the observable estimation function of our Quantum computational spectroscopy in the main text.

3.4 Estimation of general non-Hermitian Hamiltonians

We would clarify the generality of QCS by applying it to the general non-Hermitian system with non-vanishing imaginary parts of eigenenergies. Supposing the eigenenergy has imaginary part and gapped in the real part (i.e. eignenergy $E_n + i\lambda_n$ with $\lambda_n \neq 0$ and $|E_n - E_m| > 1/\tau$), the QACF with Gaussian window $\mathcal{G}(t,\tau) = \exp[-t^2/(2\tau^2)]/(\sqrt{2\pi}\tau)$ can be written as:

$$C(t) = \int \frac{d\eta}{\sqrt{2\pi}\tau}\mathcal{G}(\eta,\tau)\langle\psi|e^{iH_{NH}^{\dagger}\eta}\hat{O}e^{-iH_{NH}(\eta+t)}|\psi\rangle$$
$$= \int \frac{d\eta}{\sqrt{2\pi}\tau}\mathcal{G}(\eta,\tau)\sum_n\sum_m e^{i(E_n-i\lambda_n)\eta}e^{-i(E_m+i\lambda_m)(\eta+t)}\langle\psi|l_n\rangle\langle r_n|\hat{O}|r_m\rangle\langle l_m|\psi\rangle \tag{35}$$
$$= \sum_n\sum_m e^{-\frac{1}{2}(E_n-E_m)^2\tau^2}e^{\frac{1}{2}(\lambda_n+\lambda_m)^2\tau^2}e^{-i(E_n-E_m)(\lambda_n+\lambda_m)\tau^2}e^{i(E_m+i\lambda_m)t}\langle r_n|\hat{O}|r_m\rangle c_n^* c_m$$

where $c_n = \langle l_n|\psi\rangle$, $|r_n\rangle$ and $\langle l_n|$ are the right (physical eigenstates) and left eigenvector of the non-Hermitian Hamiltonian $\hat{H}_{NH}$, respectively. Owing to the existence of imaginary energy part, the QACF experiences two influencing factors.

First, the term $e^{\frac{1}{2}(\lambda_n+\lambda_m)^2\tau^2}$ generated by damping rates (imaginary parts) weaken the exponential suppression $e^{-\frac{1}{2}(E_n-E_m)^2\tau^2}$ of the cross term. Even so, unlike the previous time-correlation-based methods (Ref. [3–6, 9]), there is still no misleading frequency components (excitation energy peaks) emerging in the spectrum. Hence, the real part of eigenenergy can still be estimated accurately in this case. The accuracy of the estimation of expectation value $\langle r_n|\hat{O}|r_n\rangle$ would be affected, since the damping rate broaden the eigen-spectrum lines, magnifying the influences of the cross term. This affect can only be completely eliminated in a special case that if $|E_n - E_m| > |\lambda_n + \lambda_m|$, we can completely solve this problem by increasing τ. In other cases, we should choose an intermediate τ to balance the two terms $e^{-\frac{1}{2}(E_n-E_m)^2\tau^2}$ and $e^{-\frac{1}{2}(\lambda_n+\lambda_m)^2}$.

Second, the term $e^{i(E_n-E_m)(\lambda_n+\lambda_m)\tau^2}$ would give a complex-number spectrum. The windowed Fourier transformation of this QACF reads:

$$\widetilde{C}(\omega) = \sum_n\sum_m e^{-\frac{1}{2}(\omega-E_m)^2\tau^2}e^{-\frac{1}{2}(E_n-E_m)^2\tau^2}e^{\frac{1}{2}(\lambda_m^2+(\lambda_n+\lambda_m)^2)\tau^2}e^{i((\omega-E_m)\lambda_m+(E_n-E_m)(\lambda_n+\lambda_m))\tau^2}O_{nm}c_n^* c_m \tag{36}$$

where $O_{nm} = \langle r_n|\hat{O}|r_m\rangle$. The main contribution in the spectrum comes from the diagonal terms (cross term is suppressed to some degree):

$$diag.\ term = \sum_n e^{-\frac{1}{2}(\omega-E_n)^2\tau^2}e^{\frac{5}{2}\lambda_n^2\tau^2}e^{i(\omega-E_n)\lambda_n\tau^2}O_{nn}|c_n|^2 \tag{37}$$

which gives the unique spectral line shape. For each peak in the spectrum, the imaginary part of eigenenergies can be obtained by fitting with the parametrized function:

$$f_{E,\lambda}(\omega) = Ae^{-\frac{1}{2}(\omega-E)^2\tau^2}e^{\frac{5}{2}\lambda^2\tau^2}e^{i(\omega-E)\lambda\tau^2} \tag{38}$$

with a indefinite parameter A. In a word, although the accuracy of the estimation of expectation value on the eigenstates would be affected by imaginary eigenenergies, we can still extract the accurate eigenenergies and acquire largely correct expectation values since the cross terms are still suppressed to some degree.

Supplementary Note 4 – The spectrum analysis for the periodically-driven system and the revealing of implicit holonomy

Time-periodic fields are widely used to coherently manipulate quantum systems, driving the system to the non-equilibrium regime and engineering the single-particle spectrum and its topological properties [10]. Such system is governed by a certain time-periodic Hamiltonian:

$$i\hbar\frac{d}{dt}|\psi(t)\rangle = \hat{H}(t)|\psi(t)\rangle,\ \ \hat{H}(t) = \hat{H}(t+T) \tag{39}$$

Although the effective time-independent Hamiltonian can be defined by the stroboscopic evolution operator

$$\hat{U}(t_0+T,t_0) = \mathcal{T}e^{-\frac{i}{\hbar}\int_{t_0}^{t_0+T}\hat{H}(t)dt} = e^{-\frac{i}{\hbar}T\hat{H}_{t_0}^F} \tag{40}$$

where $\hat{H}_{t_0}^F$ is called the Floquet Hamiltonian, the spectrum is not necessarily the same as those time-independent Hamiltonians. The difference results from the absence of energy conservation in these periodically-driven systems, since the logarithm in Supplementary Equation (40) is multivalued. In other words, any quasienergies differ from the eigenvalues of $\hat{H}_{t_0}^F$ by the driven frequency $\hbar\omega = \hbar\frac{2\pi}{T}$ could possibly exist in the spectrum. This scenario would challenge any quantum eigenvalue estimation algorithms based on the time-independent Hamiltonian, since the periodically-driven dynamics cannot be reasonably considered. On the contrary, this challenge can be naturally overcome by the Quantum computational spectroscopy, which can reveal any quasienergies contributing to the state evolution under the time-dependent Hamiltonian.

4.1 Floquet theorem and the analysis of the quasienergy spectrum

According to the Floquet theorem, the time evolution of periodically-driven systems can be expanded by the orthonormal Floquet states $\{|\psi_n(t)\rangle\}$, which satisfy the time-evolution relation through the driven period: $|\psi_n(t+T)\rangle = e^{-i\varepsilon_n T/\hbar}|\psi_n(t)\rangle$, with the Floquet quasienergy ε_n. Further, the Floquet state can be decomposed into the periodic wave function:

$$|\psi_n(t)\rangle = e^{-i\varepsilon_n t/\hbar}|u_n(t)\rangle,\ \ |u_n(t+T)\rangle = |u_n(t)\rangle \tag{41}$$

The Schrödinger equation can be rewritten as:

$$(\varepsilon_n + i\hbar\frac{d}{dt})|u_n(t)\rangle = \hat{H}(t)|u_n(t)\rangle \tag{42}$$

Using the Fourier decomposition of the periodic wave function $|u_n(t)\rangle$ and the periodically-driven Hamiltonian $\hat{H}(t)$, we can obtain that:

$$(\varepsilon_n + m\hbar\omega)|u_n^{(m)}\rangle = \sum_{m'} \hat{H}^{(m-m')}|u_n^{(m')}\rangle \tag{43}$$

where $|u_n^{(m)}\rangle = \frac{1}{T}\int_0^T dte^{im\omega t}|u_n(t)\rangle$ and $\hat{H}^{(m)} = \frac{1}{T}\int_0^T dte^{im\omega t}\hat{H}(t)$ with $\omega = 2\pi/T$. This equation can be mapped to an eigen-equation in the extended Hilbert space with infinite dimension:

$$\mathcal{H}\mathcal{U}_n = \varepsilon_n\mathcal{U}_n,\ \ \mathcal{H} = \begin{pmatrix} \ddots & \hat{H}^{(-1)} & \hat{H}^{(-2)} & \\ \hat{H}^{(1)} & \hat{H}^{(0)} - m\hbar\omega & \hat{H}^{(-1)} & \\ \hat{H}^{(2)} & \hat{H}^{(1)} & \hat{H}^{(0)} - (m+1)\hbar\omega & \hat{H}^{(-1)} \\ & \hat{H}^{(2)} & \hat{H}^{(1)} & \ddots \end{pmatrix},\ \ \mathcal{U}_n = \begin{pmatrix} \vdots \\ |u_n^{(m)}\rangle \\ |u_n^{(m+1)}\rangle \\ \vdots \end{pmatrix} \tag{44}$$

By solving this eigen problem, we can obtain the Floquet quasienergy spectrum ε_n and the corresponding Floquet states $|\psi_n(t)\rangle = e^{-i\varepsilon_n t/\hbar}\sum_m e^{-im\omega t}|u_n^{(m)}\rangle$. It's worth to note that the solution of Supplementary Equation (44) is overcomplete, since quasienergies $\varepsilon_n + m\hbar\omega$ would lead to the same Floquet state $|\psi_n(t)\rangle$. Hence, the distinct Floquet states can be indexed by the quasienergies falling in the first Floquet-Brillouin zone $\varepsilon_{min} \leq \varepsilon \leq \varepsilon_{min} + \hbar\omega$ and all quasienergies form the band structure in the periodically-driven system's spectrum.

In general, the time-evolution of the initial state under the periodically-driven Hamiltonian would generate oscillations at any frequency falling in the first Floquet-Brillouin zone and its shifted zones by driven frequency ω. For a certain initial state expanded by the distinct Floquet states $|\psi\rangle = \sum_n c_n|\psi_n(0)\rangle$, the time-evolution of the initial state can be written as $\hat{U}(t,0)|\psi\rangle = \sum_n c_n e^{-i\varepsilon_n t/\hbar}\sum_m e^{-im\omega t}|u_n^{(m)}\rangle$. Obviously, the quantum auto-correlation function in our Quantum computational spectroscopy can also eliminate the cross-correlation signal between different quasienergies:

$$C_{\hat{I},\psi}(t) = \int d\eta\langle\psi|\hat{U}^{\dagger}(\eta,0)\hat{U}(\eta+t,0)|\psi\rangle = 2\pi\delta(0)\sum_{n,m}|c_n|^2 e^{-i\varepsilon_n t/\hbar}e^{im\omega t}\langle u_n^{(m)}|u_n^{(m)}\rangle \tag{45}$$

where $|u_n^{(m)}\rangle$ is not necessarily normalized or orthogonal to each other since they are just the Fourier coefficients of normalized periodic wave function $|u_n(t)\rangle$. Therefore, the partial band structure supported by the initial state (those $c_n \neq 0$) of the Floquet system can be easily revealed by Fourier transforming the quantum auto-correlation function.

4.2 The implicit holonomy of topologically dynamical systems in the Floquet spectrum

The remarkable discovery of Berry's U(1) geometric phase reveals the gauge structure naturally existing in the slowly varying quantum system along a circuit in the parameter space. As shown in Berry's initial paper [11], this novel topological property can be seen in a model Hamiltonian defined by a three-dimensional parameter $\boldsymbol{R}$:

$$\hat{H}(\boldsymbol{R}) = \boldsymbol{R}\cdot\boldsymbol{\sigma},\ \ \boldsymbol{R} = R(\sin\theta\cos\phi, \sin\theta\sin\phi, \cos\theta),\ \ \boldsymbol{\sigma} = (\sigma_x, \sigma_y, \sigma_z) \tag{46}$$

The eigenvalues of the Hamiltonian are only determined by the norm of the parameter: $E_+(\boldsymbol{R}) = -E_-(\boldsymbol{R}) = R$, and they are degenerate at the origin. If the eigenstate is adiabatically driven alone a closed circuit C in the parameter space, it would acquire an additional geometric phase $\gamma_\pm(C) = \mp\frac{1}{2}\Omega(C)$ ($\Omega(C)$ is the solid angle that C subtends at the degeneracy point), except for the dynamical phase. Explicitly, when the circuit C is an equator around the origin, the geometric phase would be $\gamma_\pm(C) = \mp\pi$.

Actually, this topological manner can be understood in the way of the periodically-driven system, since the system's Hamiltonian would come back to the same form after a period T. Suppose that the system is periodically driven along an equator in the parameter space, the time-dependent Hamiltonian can be written as:

$$\hat{H}(t) = \frac{1}{2}R\begin{pmatrix} 0 & e^{-i\omega t} \\ e^{i\omega t} & 0 \end{pmatrix} \tag{47}$$

the Floquet extended Hamiltonian of which possesses a simple tridiagonal form:

$$\mathcal{H} = \begin{pmatrix} \ddots & \ddots & & & & & & \\ \ddots & \omega & 0 & & & & & \\ & 0 & \omega & R & & & & \\ & & R & 0 & 0 & & & \\ & & & 0 & 0 & R & & \\ & & & & R & -\omega & 0 & \\ & & & & & 0 & -\omega & \ddots \\ & & & & & & \ddots & \ddots \end{pmatrix} \tag{48}$$

The eigen problem of this extended Hamiltonian can be solved easily by reducing it to the eigen problem of a general block diagonal form $\begin{pmatrix} -m\omega & R \\ R & -(m+1)\omega \end{pmatrix}$, giving the quasienergies $\varepsilon_{m,\pm} = -(m+\frac{1}{2})\omega \pm \sqrt{R^2 + \omega^2/4}$. The distinct Floquet states can be indexed by

$\varepsilon_{\pm} = \frac{1}{2}\omega \pm \sqrt{R^2 + \omega^2/4}$, corresponding to the gapped eigenstates of $\hat{H}(\boldsymbol{R})$ labeled by $E_{\pm}(\boldsymbol{R}) = \pm R$. It is precisely because the Berry phase accumulating in a period is π, the quasienergy is shifted by $\frac{1}{2}\omega$ in the Floquet spectrum rather than the driven frequency ω, which can be seen more clearly when taking $\omega/2R \ll 1$, $\varepsilon \approx \frac{1}{2}\omega \pm R$. In addition, the nonlinear deviation term $\sqrt{R^2 + \omega^2/4}$ originates from the interaction between two eigenstates through the non-adiabatic transport, which would approach to the static eigenvalues when the driven frequency is enough small relative to the energy gap $2R$. Hence, the analysis of Floquet spectrum can reveal this U(1) holonomy by testing the shift of eigenenergies if we drive the system slowly enough. It is worth to note that the U(1) holonomy results in a single shift for each eigenstates in a Floquet band and the interval of Floquet bands equals to the driven frequency and the accumulated phase γ can be related to the eigenenergy shift $\Delta\omega$ by the driven frequency: $\Delta\omega = \frac{\gamma}{2\pi}\omega$.

The topological manner can be further generalized to more complex holonomy when eigenstates forms the multiple degeneracy, as Wilczek and Zee shows [12]. We take the model Hamiltonian of spin-$\frac{3}{2}$ atom's nuclear quadrupole resonance for example, $\hat{H}_{NQR} = (\boldsymbol{B}\cdot\hat{\boldsymbol{S}})^2$, to reveal the SU(2) holonomy in the corresponding Floquet spectrum. Firstly, we briefly introduce the gauge structure in the parameter space of $\boldsymbol{B} = B(\sin\theta\cos\phi, \sin\theta\sin\phi, \cos\theta)$. Taking the four-dimensional representation of the spin operator $\hat{\boldsymbol{S}}$ for spin-$\frac{3}{2}$ atom:

$$\hat{S}_x = \begin{pmatrix} 0 & \frac{\sqrt{3}}{2} & 0 & 0 \\ \frac{\sqrt{3}}{2} & 0 & 1 & 0 \\ 0 & 1 & 0 & \frac{\sqrt{3}}{2} \\ 0 & 0 & \frac{\sqrt{3}}{2} & 0 \end{pmatrix}, \quad \hat{S}_y = \begin{pmatrix} 0 & -i\frac{\sqrt{3}}{2} & 0 & 0 \\ i\frac{\sqrt{3}}{2} & 0 & -i & 0 \\ 0 & i & 0 & -i\frac{\sqrt{3}}{2} \\ 0 & 0 & i\frac{\sqrt{3}}{2} & 0 \end{pmatrix}, \quad \hat{S}_z = \begin{pmatrix} \frac{3}{2} & 0 & 0 & 0 \\ 0 & \frac{1}{2} & 0 & 0 \\ 0 & 0 & -\frac{1}{2} & 0 \\ 0 & 0 & 0 & -\frac{3}{2} \end{pmatrix} \tag{49}$$

then we can easily find two double-degenerate bands with different eigenenergies $E_{\pm\frac{1}{2}}(\boldsymbol{B}) = B^2/4$, $E_{\pm\frac{3}{2}}(\boldsymbol{B}) = 9B^2/4$, the corresponding eigenstates can be written as $|\pm\frac{1}{2}(\boldsymbol{B})\rangle = e^{-i\phi\hat{S}_z}e^{-i\theta\hat{S}_y}|\boldsymbol{e}_{1,2}\rangle$, $|\pm\frac{3}{2}(\boldsymbol{B})\rangle = e^{-i\phi\hat{S}_z}e^{-i\theta\hat{S}_y}|\boldsymbol{e}_{0,3}\rangle$, where $|\boldsymbol{e}_i\rangle = (\delta_{0,i}, \delta_{1,i}, \delta_{2,i}, \delta_{3,i})^T$. The Wilczek-Zee phase factor or the SU(2) holonomy in the adiabatic transport along a closed circuit C in the parameter space can be defined in the degenerate subspace:

$$\mathrm{U}_{WZ,ab} = \mathcal{P}\exp[i\oint_C \boldsymbol{A}_{\mu,ab}dx^\mu], \quad \boldsymbol{A}_{\mu,ab} = i\langle\phi_a(x)|\partial_\mu|\phi_b(x)\rangle \tag{50}$$

where $|\phi_a(x)\rangle$ is the eigenstate in a certain degenerate subspace and $\boldsymbol{A}$ is the gauge potential to show the gauge structure in the space of parameter x^μ. Since the degeneracy leads to the nonscalar gauge potential, the WZ phase factor forms a SU(2) holonomy. In the gauge defined by the eigenstates $|\pm\frac{1}{2}(\boldsymbol{B})\rangle$ and $|\pm\frac{3}{2}(\boldsymbol{B})\rangle$, the gauge potential can be easily calculated for two degenerate bands: $\boldsymbol{A}_\mu^{\frac{1}{2}}dx^\mu = (\frac{\cos\theta}{2}\sigma_z - \sin\theta\sigma_x)d\phi + \sigma_y d\theta$, $\boldsymbol{A}_\mu^{\frac{3}{2}}dx^\mu = \frac{3\cos\theta}{2}\sigma_z d\phi$. Note that there exist singularities both at the north pole ($\theta = 0$) and south pole ($\theta = \pi/2$), then this local gauge is defined on a patch which is not simply connected. Hence, we take a gauge transformation $W_N^{\frac{1}{2}} = e^{i\sigma_z\phi/2}$ and $W_N^{\frac{3}{2}} = e^{i3\sigma_z\phi/2}$ to remove the singularity at the north pole, then the WZ phase factor in the new gauge is written as $\mathrm{U}'_{WZ} = W^{\dagger}_{N,C_f}\mathrm{U}_{WZ}W_{N,C_i}$, where C_f and C_i are the final and initial ends of the closed circuit C.

We reveal the implicit holonomy of the parameterised NQR Hamiltonian in the main text for different cases with fixed θ and varying ϕ. When driving the parameter along the equator ($\theta = \pi/2$), the WZ phase factor of two degenerate bands can be written as:

$$\mathrm{U}'_{WZ,\frac{1}{2}}\Big|_{\theta=\pi/2} = e^{i\pi\sigma_z}e^{-i2\pi\sigma_x} = \begin{pmatrix} e^{i\pi} & 0 \\ 0 & e^{i\pi} \end{pmatrix}, \quad \mathrm{U}'_{WZ,\frac{3}{2}}\Big|_{\theta=\pi/2} = e^{i3\pi\sigma_z} = \begin{pmatrix} e^{i\pi} & 0 \\ 0 & e^{i\pi} \end{pmatrix} \tag{51}$$

Since the transport along the equator only contributes the diagonal phase factors on each eigenstates, the U(1) holonomy can be genuinely revealed in the Floquet spectrum with a single eigenenergy shift near $\frac{1}{2}\omega$ and Floquet bands seperated by the driven frequency as shown in our experiment. The true SU(2) holonomy could emerge when the parameter driven along the fixed-θ circle (taking $\theta = \pi/4$). The WZ factor can be written as:

$$\begin{aligned} \mathrm{U}'_{WZ,\frac{1}{2}}\Big|_{\theta=\pi/4} &= e^{i\pi\sigma_z}e^{i2\pi(\frac{\cos\frac{\pi}{4}}{2}\sigma_z - \sin\frac{\pi}{4}\sigma_x)} = \begin{pmatrix} -0.2522+0.4328i & -0.8655i \\ -0.8655i & -0.2522-0.4328i \end{pmatrix} \\ \mathrm{U}'_{WZ,\frac{3}{2}}\Big|_{\theta=\pi/4} &= e^{i3\pi\sigma_z}e^{i3\pi\cos\frac{\pi}{4}\sigma_z} = \begin{pmatrix} e^{i\frac{3\sqrt{2}-2}{2}\pi} & 0 \\ 0 & e^{i\frac{6-3\sqrt{2}}{2}\pi} \end{pmatrix} \end{aligned} \tag{52}$$

It is obvious that the true SU(2) holonomy is only existing in the lower degenerate band expanded by $|\pm\frac{1}{2}(\boldsymbol{B})\rangle$, while the actual U(1) holonomy of the upper band would just cause the eigenenergy shift near $\Delta\omega = \frac{3\sqrt{2}-2}{4}\omega$ for $|+\frac{3}{2}(\boldsymbol{B})\rangle$ and $\Delta\omega = \frac{6-3\sqrt{2}}{4}\omega$ for $|-\frac{3}{2}(\boldsymbol{B})\rangle$ in the Floquet spectrum. Since the SU(2) holonomy leads to the rotation between two degenerate eigenstates $|\pm\frac{1}{2}(\boldsymbol{B})\rangle$, it would cause the eigenenergy splitting in the Floquet band and the sub-band structure would emerge. The splitting can be estimated by the eigenvalues of $\mathrm{U}_{WZ,\frac{1}{2}}\Big|_{\theta=\pi/4}$, which equals $\exp[i\gamma_\pm] = \exp[\pm i\frac{\sqrt{10}-2}{2}\pi]$. That means when we drive the system for a period, there exists two components accumulating different phase, γ_+ and γ_-, respectively, which causes the eigenenergy splitting $\Delta\omega_{split} = \frac{\gamma_+ - \gamma_-}{2\pi}\omega = \frac{\sqrt{10}-2}{2}\omega$. Additionally, the phase factor of SU(2) holonomy is not gauge-invariant any more, while a gauge invariant related to this factor is the Wilson loop $\mathcal{W} = Tr[\mathrm{U}_{WZ,\frac{1}{2}}\Big|_{\theta=\pi/4}] = 2\cos\gamma_+ = 2\cos(\frac{\Delta\omega_{split}}{\omega}\pi) \approx -0.504$, which can be directly figured out through the analysis of the Floquet spectrum. In our experiment, we drive the parameter along the fixed-θ circle by varying ϕ periodically with frequency $\omega = 0.5$ as the simple case mentioned above to show the Berry phase. Also, the nonadiabatic transport would cause the interaction between two degenerate bands, which leads to slight deviation from the eigenenergy shift or splitting calculated by the theoretical WZ phase factor. But it can be corrected at enough precision by solving the extended eigen-equation in Supplementary Equation (44) with high-frequency truncation for $|m| > 5$, see main text.

Supplementary Note 5 – Hardware scalability of the silicon photonic quantum chip for QCS

High-fidelity arbitrary controlled unitary operators remain challenging on current quantum platforms, including superconducting, trapped-ion, and atomic systems. Nevertheless, the integrated-photonic quantum chip realizations in our work demonstrate this capability—even at a small scale. We emphasize that the experimental demonstrations reported here are proof-of-principle, involving small effective system sizes that remain well within the classically simulable regime. Scaling such devices to larger system sizes remains an experimental challenge but it is possible. In this section, we analyze potential scaling pathways for our integrated photonic platform.

5.1 Hardware limitations of the current device

Before discussing potential scaling pathways, we first acknowledge two major practical limitations that currently prevent immediate large-scale demonstrations on our current integrated photonic device and platform:

Device losses: Propagation loss in silicon-on-insulator (SOI) waveguides of the current chip is about 2.0dB/cm. Each MZI introduces an insertion loss of approximately 0.46 dB, and the chip-fibre coupling loss is about 4 dB. In the current chip design, the photon carrying the target qudit experiences one asymmetric MZI for spectral filtering and four layers of linear optical network (preparation, unitary, compression, and measurement, as shown in Fig. 2a in the main text), with about 2.5 dB insertion loss, resulting in a total 14 dB insertion loss for the target photon. For the compression operation, we coherently compress the expanded Hilbert space to superpose four unitary operations (U_0–U_3) for the realisation of controlled time evolution. This compression operation projectively collapses the state into a quarter of the subspace, introducing an additional 6 dB loss for the target photon. Hence, the target photon experiences about 20 dB loss, and the ancilla photon about 10 dB loss, resulting in a total coincidence loss of 30 dB. Overcoming this challenge would require better devices and advanced schemes, which are not implemented in the current design.

Probabilistic multi-Photon generation: Our current platform relies on probabilistic spontaneous four-wave mixing (SFWM). To suppress multi-pair emission noise, the generation probability per pulse is typically restricted to $P \approx 0.01$–0.05. For an $N = 6$ photon state, the coincidence rate drops to less than 0.1 Hz, making experiments at $N \geq 8$ practically infeasible without deterministic sources or active multiplexing (see below more details). Also, because the multi-photon source we mentioned above would be more vulnerable to the purity of photons, the additional noise induced by this make the current integrated multi-photon platform not suitable for the precise spectrum estimation in QCS.

Thermal crosstalk and calibration overheads: Thermo-optic phase tuning dissipates about 30 mW of electrical power per heater. For high-dimensional encoding of quantum states, the required number of MZIs increases dramatically. We operate 108 MZIs on the current chip. When integrating larger devices with more heaters (see the discussion of scaling possibilities in the following sections), tuning many more MZIs simultaneously will cause thermal gradients and crosstalk across the chip. Beyond crosstalk, the challenge lies in how to electronically drive a large number of thermo-optic phase shifters in a scalable manner. Managing thermal crosstalk and implementing scalable electronic controls must be addressed as the system dimension scales. Fortunately, thermal crosstalk can be mitigated through techniques such as deep-trench thermal isolation, while scalable electronic control can be effectively supported by advanced electronic–photonic co-packaging technologies.

5.2 The scaling of dimensions of integrated photonic unitary network

The achievable dimensionality of the high-dimensional entanglement as well as the corresponding high-dimensional controlled-unitary operation circuit, are determined by several factors: the circuit's physical footprint, the optical loss throughout the circuit, and the success probability for generating N-dimensional entangled states.

(1) Circuit footprint: The dimensionality of integrated high-dimensional entanglement sources, as well as the corresponding high-dimensional controlled-unitary operation circuits, is determined by the number of SFWM processes and the number of spatial paths for the N-dimensional unitary circuit. This means that the achievable dimensions mainly depend on the size of the MZIs relative to the silicon photonic chip area and, more generally, on the number of integrated photonic components available on a silicon chip. To achieve arbitrary control over the unitary operations – which is essential for implementing the QCS – the N-dimensional circuit requires $N \times (N-1)/2$ MZIs. For a fixed MZI size (currently about $300\mu m \times 60\mu m$), the length or width of the network scales linearly with the dimension N. One die fabricated using our CMOS photolithography processes (see Methods) on a 200 mm diameter silicon-on-insulator wafer is approximately $24mm \times 32mm$. Thus, a single chip can accommodate $N > 100$ dimensions, as $N = 32mm/300\mu m \approx 107$. By using similar fabrication technology, previous works have previously demonstrated 16-dimensional quantum entanglement generation[13] and 32-dimensional quantum graph lattices[14], for different purposes but indicating the dimension scalability. Moreover, employing die-to-die stitching techniques[15], where the coupling loss can be optimized down to about 0.004 dB, enables further expansion of the accessible dimensionality. In addition, reducing the footprint of basic beam splitters can dramatically enhance dimensional scalability. On the current chip, multimode interferometers (MMIs) of size $27\mu m \times 2.8\mu m$ are used, while recent work reports an inverse-designed MMI as compact as $4\mu m \times 2\mu m$[16], suggesting that dimensionality can be increased by roughly an order of magnitude.

(2) Loss analysis: The total loss of the high-dimensional controlled-unitary device is primarily determined by the number of MZIs in the circuit. In our current device, the MMI loss was optimized to approximately 0.2dB, while the typical loss of each MZI, accounting for a waveguide propagation loss of 2dB/cm, was about 0.46dB. As a result, the overall loss of an $N \times N$ unitary circuit scales roughly as $0.46N$dB, which inevitably reduces the detectable photon count rate. Strategies for loss management include: replacing MMIs with ultra-low-loss directional couplers (albeit at the cost of stricter fabrication tolerances); adopting multimode waveguides, which have demonstrated propagation losses as low as 0.28dB/cm[17]; migrating to ultra-low-loss silicon nitride integrated photonic platforms. For instance, a 20-dimensional unitary network implemented on a silicon nitride platform with a propagation loss of 0.07dB/cm has achieved a total loss of only 1.1dB[18] – about

an order of magnitude lower than comparable silicon-based designs. It should be noted, however, that the silicon nitride platform typically offers lower integration density, which may entail a trade-off in the achievable dimensionality of the unitary circuit when compared with high-density silicon photonics.

(3) Entanglement probability: The success probability of generating N-dimensional entanglement does not decay rapidly with increasing dimension. For two-photon high-dimensional entangled states, we pump an array of N SFWM-based photon-pair sources simultaneously, where each source (or spatial mode) corresponds to one encoding basis state $|0\rangle, |1\rangle, ..., |N-1\rangle$. The N-dimensional entanglement is effectively the coherent superposition of "single-source pumping" events, i.e., events in which exactly one source emits an entangled photon pair. If the probability of generating a photon pair from a single SFWM source is p, the probability of obtaining a two-photon N-dimensional entangled state is given by

$$\text{Prob}_{\text{success}} = Np(1-p)^{N-1}. \tag{53}$$

When p is sufficiently small, this expression can be approximated as

$$\text{Prob}_{\text{success}} \approx Np * e^{-p(N-1)}, \tag{54}$$

which reaches a maximum value of approx. $\frac{N}{e(N-1)} \approx \frac{1}{e}$ when $p = 1/(N-1)$. Given that the photon-pair generation probability scales quadratically with the pump power ($\text{Power}_{\text{pump}}$), i.e, $p \propto \text{Power}^2_{\text{pump}}$, the optimal total pump power required for N sources is

$$\text{Power}_{\text{total}} = N\text{Power}_{\text{pump}} \propto N\sqrt{p} = \frac{N}{\sqrt{N-1}} \approx \sqrt{N}. \tag{55}$$

Therefore, by scaling the total pump power as $\sqrt{N}$ (which is favorable), the success probability of generating N-dimensional entanglement can be maintained near the constant value $1/e$.

In addition, given the efficienct generation of high-dimensional entanglement, the high-dimensional controlled-unitary operation is then realized through Hilbert-space expansion and coherent compression processes applied to the high-dimensional entangled state, as shown in Fig. 2a in main text. With the current device design and the measurement configuration, this process achieves a success probability of $1/2$. The probability can be improved by optimizing the compression stage. Using a general high-dimensional Hadamard-type measurement scheme will result in a deterministic—i.e., 100%—success probability for the high-dimensional controlled unitary operation[19].

5.3 The scaling of multi-photon high-dimensional quantum chip

When using probabilistic photon sources, the detection rate for n-photon coincidence events scales exponentially. This poses a well-known challenge for scaling photonic quantum information processing. The computational capacity of a photonic quantum processor is determined by the size of its accessible Hilbert space, which scales as d^n for n qudits of dimension d. The generalized multi-qudit controlled-unitary operation – the key operation for QCS in larger quantum systems – can be realized using one auxiliary qudit to control n target qudits. This scheme avoids the need for direct qudit-qudit interactions after state preparation and local operations, eliminating a key scaling bottleneck. The operation can be generalized employing an n-photon d-dimensional Greenberger–Horne–Zeilinger (GHZ) state as the resource[19]:

$$|\text{GHZ}(n,d)\rangle = \frac{1}{\sqrt{d}} \sum_{i=0}^{d-1} |i\rangle^{\otimes n}. \tag{56}$$

Experimentally, high-dimensional multi-photon GHZ states have already been realized in bulk optics[20–22] and on integrated photonic chips[14]. Scaling to more photons builds on established techniques for multi-qubit GHZ generation[23], which rely on high-purity, highly indistinguishable photon-pair sources. Recent advances in integrated quantum photonics have demonstrated increasingly complex multi-photon states, including 4-photon states[24,25], 8-photon states[26], 18-photon device[27], and high-purity high-indistinguishability photon-pairs[28]. By integrating these technologies, including multi-photon qudit GHZ generation, high-fidelity qudit control, high-quality photon sources, low-loss fiber–chip coupling, and large-scale integration, we estimate that a 10-photon device is achievable in the near term, as detailed in the following analysis. The photon rate for realizing this generalized multi-qudit controlled-unitary operation using the $|\text{GHZ}(n,d)\rangle$ entangled state can be expressed as follows:

$$R_{tot}(n,d) = R_{GHZ}(n,d) \cdot \eta_{compress}(d) \cdot \eta_{cir}(n,d), \tag{57}$$

where $R_{GHZ}(n,d)$ denotes the generation rate of the n-photon d-dimensional GHZ resource state, $\eta_{compress}(d)$ presents the success probability of the coherent compression process (that is $1/d$) and $\eta_{cir}(n,d)$ denotes the propagation loss. The GHZ rate can be estimated as[20,21]:

$$R_{GHZ}(n,d) = R_0 \cdot (\eta_{herald}\eta_{OI}\eta_{det})^n \cdot p_0^{n/2} \cdot \frac{d}{d^n}, \tag{58}$$

where R_0 is the repetition rate of the pulsed pump laser (500MHz in our case), η_{herald} is the heralding efficiency of photon-pair sources (0.9 efficiency has been reported in silicon[28]), η_{OI} is the coupling efficiency between silicon chip and optical fiber (0.9 for low-loss edge coupling), η_{det} is the detection efficiency (0.9 for the typical SNSPD), p_0 denotes the probability of photon-pair generation and $\frac{d}{d^n}$ presents the post-selection probability of generating the GHZ state. The $\eta_{cir}(n,d)$ is estimated by:

$$\eta_{cir}(n,d) = (\eta_{MZI})^{2dn}, \tag{59}$$

where the total circuit loss on the n-photon d-dimensional GHZ state is the product of the loss of cascaded MZIs and all n photons and η_{MZI} is estimated as 0.98 ($\approx 0.1dB$) for low-loss realisation in silicon. When $p_0 = 0.667$ can be chosen according to the state-of-the-art multiplexing

photon-pair source (demonstrated in the bulk-optics system[29]), we estimate the scaling of total detection rate for different photon number and dimensions as shown in Supplementary Figure 5.

However, we would like to emphasize that this exponential bottleneck is not a fundamental limitation of photonic systems, but rather a challenge associated with probabilistic generation. While current probabilistic sources impose exponential scaling on coincidence rates, the integration of deterministic sources, advanced multiplexing, or alternative continuous-variable architectures represents a speculative pathway that will require substantial experimental progress to realize at scale.

- **Deterministic or multiplexed single-photon sources:** Semiconductor quantum dot sources are achieving near-unity efficiency and high indistinguishability. A cluster-state gun scheme immediate with a single electron spin can achieve 1D multi-photon cluster state entanglement[30,31]. Recent work reports spontaneous two-photon emission with a single high-brightness semiconductor quantum dot[32]. These demonstrations have shown the possibility of realising deterministic generation of multi-photon entanglement. Additionally, multiplexed single-photon sources and entangled states have been proposed or experimentally demonstrated with improved efficiency. While not yet near-deterministic, these systems show promising progress in both bulk-optics[29] and integrated optics[33]. Moreover, heralded generation of qudit GHZ states has been proposed as a pathway to improve photon-number scaling, leveraging multiplexing techniques to boost efficiency[34].

- **Continuous-variable quantum chips**: The rapid development of continuous-variable (CV) quantum photonics offers a complementary and scalable architecture. This scalability stems from the deterministic generation of squeezed states (serving as the fundamental quantum units, i.e., qumodes), combined with deterministic entangling operations between these qumodes. Recent advances further strengthen this platform: notable achievements include the integrated photonic continuous-variable time-spatial cluster state demonstrated by Xanadu[35,36], as well as the realization of frequency-bin cluster states[37] and spatial cluster states[38]. This integrated continuous-variable photonic platform presents a highly promising alternative route for implementing the QCS.

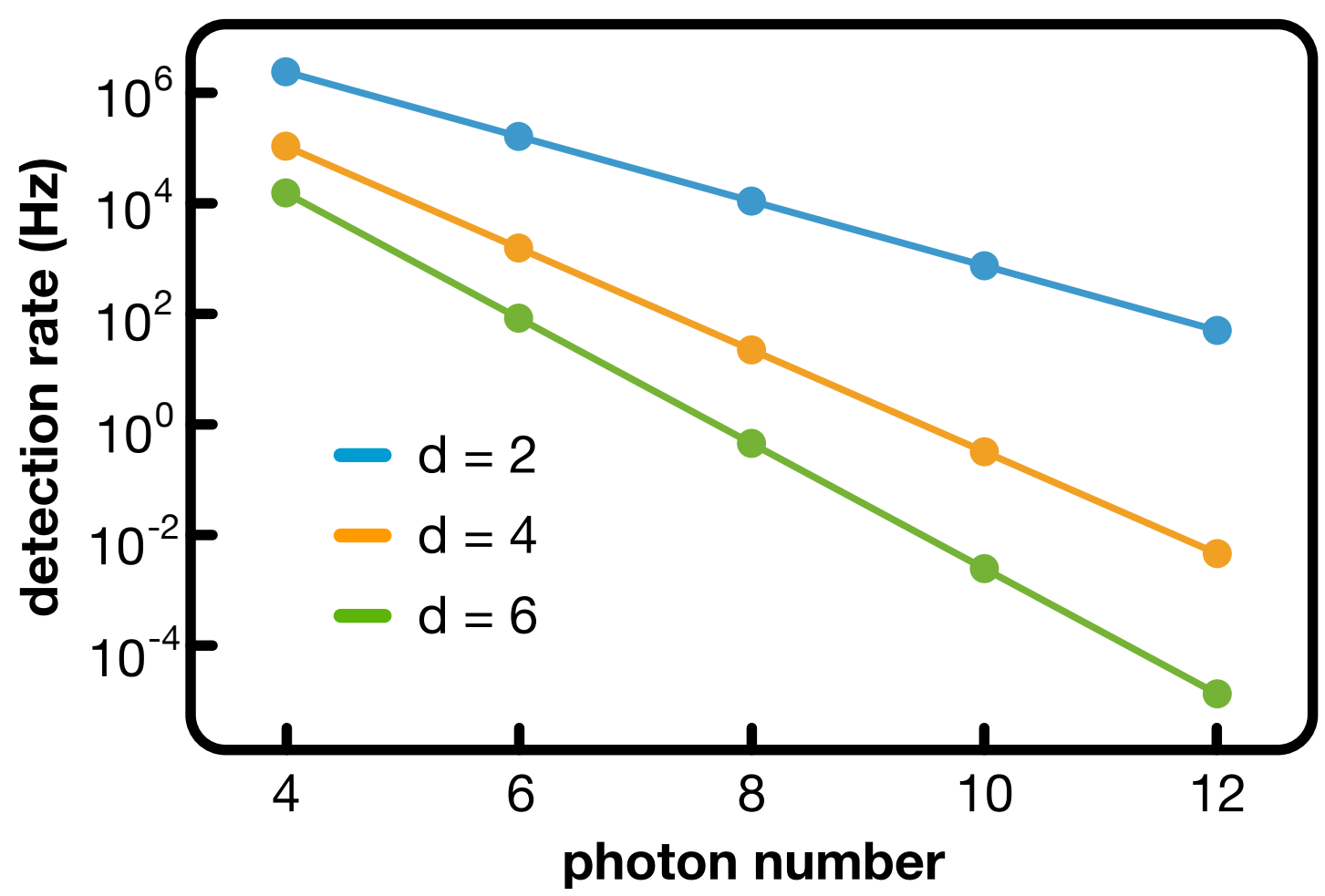

Supplementary Figure 5 Estimated photon detection rate of the generalized multi-qudit controlled-unitary operation for different photon numbers and dimensions.

5.4 Categorizing scaling pathways

To provide a clear assessment, we organize the discussion into three categories: (1) what has been experimentally demonstrated elsewhere, independent of the present work; (2) what is possible in principle, based on theoretical or conceptual arguments; (3) what may be achievable with future hardware advances, which remains speculative at this stage.

- **What has been demonstrated elsewhere:** Several key capabilities required for scaling QCS have already been experimentally demonstrated in other research efforts.
  (1) High-dimensional unitary networks: Previous works have demonstrated 16-dimensional quantum entanglement generation[13] and 32-dimensional quantum graph lattices[14] on integrated photonic platforms, indicating the feasibility of scaling dimensionality.
  (2) Low-loss components and platforms: Compact MMIs as small as 4 μm $\times$ 2 μm have been reported[16]. Die-to-die stitching techniques have achieved coupling losses as low as 0.004 dB[15]. A 20-dimensional unitary network implemented on a silicon nitride platform with propagation loss of 0.07 dB/cm achieved a total loss of only 1.1 dB[18].
  (3) Multi-photon and high-dimensional entangled states: High-dimensional multi-photon GHZ states have been realized on integrated photonic chips[14]. Recent advances have demonstrated 4-photon states[24,25], 8-photon states[26], and 18-photon devices[27], as well as high-purity, high-indistinguishability photon-pair sources[28].
  (4) Deterministic multi-photon sources: Quantum dot sources have achieved near-unity efficiency and high indistinguishability, with demonstrations of deterministic multi-photon entanglement generation[30,31] and spontaneous two-photon emission from a single dot[32].
  (5) Continuous-variable paltforms: Integrated continuous-variable platforms have demonstrated deterministic generation of squeezed states and entangling operations, including time–spatial cluster states[35,36], spatial cluster states[38], and frequency-bin cluster states[37].

- **What is possible in principle:** Several scaling arguments suggest that large-scale implementation of QCS is not fundamentally prohibited.
(1) Circuit footprint scaling: An N-dimensional controlled-unitary circuit requires $N \times (N-1)/2$ MZIs. With current MZI dimensions (approximately 300 μm $\times$ 60 μm), a standard CMOS-fabricated die (approximately 24 mm $\times$ 32 mm) could theoretically accommodate $N > 100$ dimensions (32 mm/300 μm $\approx$ 107). By employing die-to-die stitching techniques[15], a total of $N \times M$ dimensions is achievable in principle, where M is the number of stitched dies. For example, when stitching four dies, a device with approximately 400 dimensions is possible.
(2) Entanglement probability: For two-photon N-dimensional entanglement generated by pumping an array of N SFWM sources, the success probability is $\text{Prob}_{\text{success}} = Np(1-p)^{N-1}$. For small p, this approximates to $\text{Prob}_{\text{success}} \approx Npe^{-p(N-1)}$, which reaches a maximum of approximately $1/e$ when $p = 1/(N-1)$. Since $p \propto \text{Power}^2_{\text{pump}}$, the required total pump power scales as $\sqrt{N}$, which is favorable. In principle, therefore, the success probability can be maintained near a constant value independent of N.
(3) Deterministic controlled-unitary operations: A general high-dimensional Hadamard-type measurement scheme could, in principle, achieve deterministic (100%) success probability for high-dimensional controlled-unitary operations[19], eliminating the $1/d$ factor from coherent compression.

- **What may be achievable with future hardware advances:** The following scaling pathways, while promising, remain speculative at present. Their practical realization will require substantial experimental progress.
(1) Deterministic high-dimensional multi-photon entanglement: Though deterministic quantum dot single-photon sources and multiplexed sources have shown promise, the deterministic generation of high-dimensional multi-photon GHZ states suitable for QCS has not yet been experimentally realized.
(2) Active thermal crosstalk mitigation at scale: While deep-trench thermal isolation and advanced electronic–photonic co-packaging have been proposed to manage thermal crosstalk, their effectiveness when scaling to thousands of MZIs has not been experimentally validated.
(3) Practical multi-photon QCS devices: Based on the technologies demonstrated elsewhere, we estimate that a 10-photon device may be achievable in the near term (see Supplementary Figure 5). However, this estimate relies on simultaneous progress across multiple fronts and has not yet been experimentally verified.

Supplementary Note 6 – Discussion and analysis of algorithmic performance

In this section, we provide a numerical benchmarking of QCS against existing quantum algorithms for spectroscopic estimation of various quantum systems. The QCS is compared with the iterative quantum phase estimation algorithm (IQPE)[39], standard QPE, and quantum eigenvalue transformation of unitary matrices (QETU)[40]. Before that, we clarify that the comparison between our hybrid analog–digital approach (which relies on continuous-time dynamics, signal processing, and classical post-processing) and fully digital, gate-based algorithms (typically analyzed in a fault-tolerant setting) remains conceptually different, as they differ in computational model and physical resources. Nevertheless, the hybrid analog–digital approach represents a promising noisy intermediate-scale quantum (NISQ) application that leverages both quantum and classical processing. The benchmarking results (summarized in Supplementary Table 1 and Supplementary Figure 6) show that the primary advantage of QCS lies in its applicability—specifically, its ability to naturally handle non-orthogonal eigenstates, non-Hermitian Hamiltonians, and time-dependent driven dynamics, where standard digital eigenvalue algorithms face fundamental formulation constraints.

As a test case for Hermitian time-independent quantum system, we adopt the spin chain Hamiltonian as same as Fig. 3f with 8 sites. We account for query errors (ϵ_q) arising from imperfections in the execution of single-step controlled unitary operations $e^{i\hat{H}\delta t}$ (δt is set as 0.4), which are fundamental to eigenenergy estimation in those algorithms. Considering experimental errors on the quantum chip, we induce the query error by adding circularly-symmetric complex Gaussian noise to each element of the query unitary: $U_{q,ij} + \epsilon_q * \mathcal{CN}(0,1)$. As shown in Supplementary Figure 6a, under this specified noise model, the error of IQPE grows significantly due to its deeper query depth requirement. QETU, based on the fuzzy bisection method[40], remains robust under small ϵ_q, while it breaks down when ϵ_q exceeds the fuzzy interval width of the approximate shifted sign function. Under these simulated conditions, the QCS maintains an estimation error within $[10^{-3}, 10^{-2}]$ due to its different query requirement and the application of singular spectrum renormalisation. Supplementary Figure 6b presents the asymptotic scaling of query depth with respect to the target estimation error (ϵ) in the absence of query errors. The query depth of QCS scales efficiently as $\mathcal{O}(\Delta^{-1}\sqrt{\log(\epsilon^{-1}\zeta^{-1}\Delta)})$ (see Methods), where $\zeta = |\langle\phi_k|\psi\rangle_T|$ denotes the overlap between the target eigenstate $|\phi_k\rangle$ and the trial state $|\psi\rangle_T$ and $\Delta = \Delta E_{min}/R(\hat{H})$ is the rescaled energy gap. Compared to the standard gate-based digital algorithms under their respective settings, QCS exhibits a different scaling behavior in terms of query depth. For the total computational time, we compare the query complexity of QPE ($\mathcal{O}(\epsilon^{-1}\zeta^{-2})$) and QETU ($\mathcal{O}(\epsilon^{-1}\zeta^{-1})$). In QCS, for time-independent Hermitian Hamiltonians, a query complexity of $\mathcal{O}(\epsilon^{-1}\zeta^{-1})$ can also be formally achieved. We stress, however, that these comparisons are based on query depth in idealized settings. In practice, the analog-digital hybrid nature of QCS, physical resource requirements, noise models, repetition rates, and classical post-processing overheads differ fundamentally from gate-based digital algorithms, making direct complexity comparisons qualitative rather than representative of an absolute scaling advantage.

We also benchmark the capabilities of QCS for non-Hermitian systems modeled in Fig. 4a. We choose the non-collapse eigenstate as the target eigenstate to estimate the real part of eigenenergy through the PT phase transition as shown in Fig. 4g. Results are shown in Supplementary Figure 6c. Under these conditions, the different algorithms exhibit distinct behaviors. For QETU, it requries query to the time-evolution operator $e^{-i\hat{H}_{NH}\delta t}$ as well as its transpose conjugate $e^{i\hat{H}^{\dagger}_{NH}\delta t}$. Because the physical eigenstate $|r_k\rangle$ of non-Hermitian Hamiltonian is not an eigenstate of its transpose conjugate $\hat{H}^{\dagger}_{NH}$, the QETU circuit fails to preserve the dual orthogonal eigenspace. This

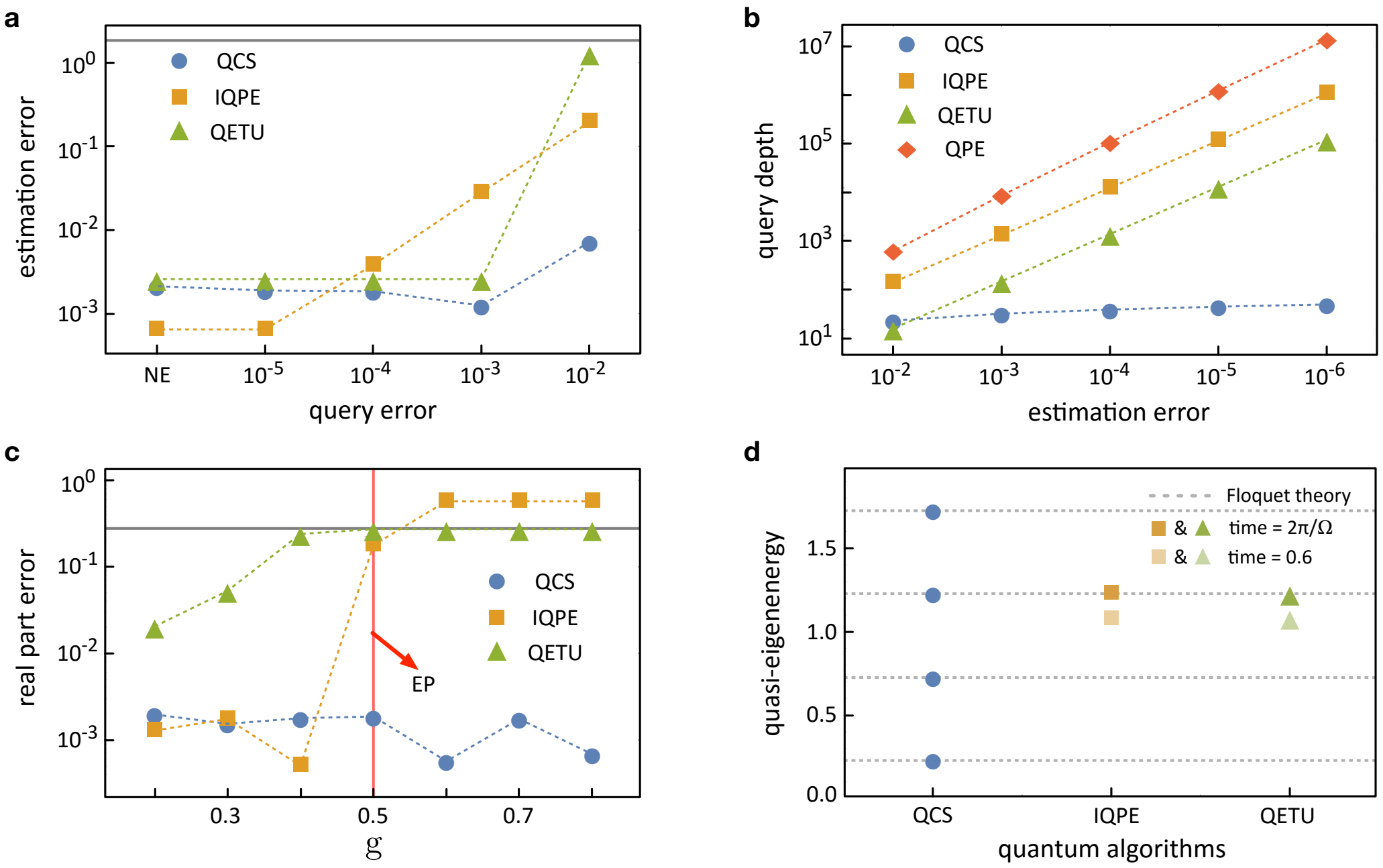

Supplementary Figure 6 Benchmarking quantum algorithms for spectroscopic estimation of general systems. a, and **b,** Ground-state energy estimation for the 8-site spin chain Hamiltonian: (a), energy estimation error (ϵ) versus query error (ϵ_q), and (b), query depth versus target estimation error ϵ with no query error ($\epsilon_q = 0$). For IQPE and QETU, we apply a shift of eigen-spectrum $\hat{H}\delta t \rightarrow \hat{H}\delta t + 11 * \hat{I}$, to ensure the rescaled ground-state energy falls within $(0, 2\pi)$. To achieve comparable estimation accuracy when no query error (NE), the query depths are chosen to {30, 2^{11}, 274} for QCS, IQPE and QETU, respectively. **c,** Estimation of eigenenergies (real part) in non-Hermitian system with no query error. While QCS can estimate the non-Hermitian eigenenergies across the PT-symmetry-breaking transition, IQPE fails when passing through the EP at $g = 0.5$, and QETU remains invalid throughout the entire parameter regime. **d,** Estimation of quasi-eigenenergy in Floquet quantum system with no query error. The dashed horizontal lines denote theoretical quasi-eigenenergies of the Floquet system. While QCS can resolve all oscillatory components associated with distinct quasi-eigenenergies, both IQPE and QETU are constrained to accurately estimating only a single quasi-eigenenergy, provided the evolution time of the query unitary matches the driving period ($2\pi/\Omega$). IQPE: iterative quantum phase estimation; QETU: quantum eigenvalue transformation of unitary matrices. In (a) and (c), The gray line indicates the initial guess bound for ground-state energy or the real part of eigenenergy. In (a)-(d), the initial state has $\zeta = 0.9$ overlap with the target eigenstate. Simulation results are obtained using MindSpore Quantum with 1000 shots per run.

fundamental limitation renders QETU invalid throughout the entire parameter regime for general non-Hermitian eigen-problems. The algorithm's relatively small estimation error observed at small g values arises solely because the non-Hermitian Hamiltonian approaches a Hermitian system in this regime. Additionally, IQPE does not need query to the transpose conjugate of the time-evolution operator, so it can estimate the real eigenenergy in the PT-exact phase. However, two fundamental limitations arise: (1) the presence of additional polynomial terms in the time-evolution operator invalidates IQPE at the EP, and (2) in the PT-broken phase, the imaginary components of eigenenergies induce ancilla qubit amplitude damping, concealing the phase information. In contrast, QCS estimates the real part of the eigenenergies across the transition without requiring the transpose conjugate or being limited by these specific constraints.

We compare the algorithms for the time-varying Floquet quantum system modelled in Fig. 4h,i. As an example, we estimate Floquet quasi-eigenenergies for the lower degenerate eigenspace with $\theta = \pi/2$, choosing the initial state $|\psi\rangle_T = e^{-i\hat{S}_y\pi/2}(0,1,0,0)^T$. Both of IQPE and QETU require a fixed time-evolution operator for their query operations, fundamentally limiting their ability to completely characterise periodically driven systems. We examine two evolution times for the query unitaries: the driving period ($\delta t = 2\pi/\Omega$), and the discrete time step used in our QCS experiment ($\delta t = 0.6$). Crucially, IQPE and QETU can only estimate eigenvalues of an effective Hamiltonian $\hat{H}_{eff}$ defined as $e^{-i\hat{H}_{eff}\delta t} = \mathcal{T}e^{-i\int_0^{\delta t}(\boldsymbol{B}(t')\cdot\hat{S})^2 \mathrm{d}t}$. When δt matches the driving period, $\hat{H}_{eff}$ correctly captures quasi-eigenenergies within a single Floquet-Bloch band[10]. However, for other δt values, they yield completely incorrect quasi-eigenenergies. This is confirmed in Supplementary Figure 6d: only QCS agrees with theoretical predictions, while IQPE and QETU either return erroneous quasi-eigenenergies or are restricted to estimating a single eigenvalue. Additionally, the capability of VQE to solve non-Hermitian eigenproblems across PT symmetric transitions remains uncertain without knowledge of appropriate ansatz. VQE is generally unsuitable for Floquet quantum systems because the effective Hamiltonian describing the full-period time evolution typically lacks a closed form.

Supplementary Table 1 Comparison of quantum algorithms for spectroscopic estimation of various quantum systems.

Quantum algorithms	Quantum systems	Query depth[a]	Excitation- or eigen-energy	State properties
QPE[41,42]	Hermitian TI	$\mathcal{O}(\epsilon^{-1}\log(\epsilon^{-1}))$	eigen	Yes
IQPE[39]	Hermitian TI, PT exact phase	$\mathcal{O}(\epsilon^{-1})$	eigen	No
QETU (prepare ground state)[40]	Hermitian TI	$\mathcal{O}(\zeta^{-2}\Delta^{-1}\log(\epsilon^{-1}\zeta^{-1}))$	eigen	Yes
QETU (estimate eigenenergy)[40]	Hermitian TI	$\mathcal{O}(\epsilon^{-1}\log(\zeta^{-1}))$	eigen	Yes
spectroscopic algorithms[4,5]	Hermitian TI	$\mathcal{O}(\Delta^{-1}\sqrt{\log(\epsilon^{-1})})$	excitation	No
VQE[43–45]	Hermitian TI[b]	ansatz-dependant	eigen	Yes
QCS (this work)	Hermitian TI, non-Hermitian, Floquet (general dynamics)	$\mathcal{O}(\Delta^{-1}\sqrt{\log(\epsilon^{-1}\zeta^{-1}\Delta)})$	eigen	Yes

IQPE: iterative quantum phase estimation; QETU: quantum eigenvalue transformation of unitary matrices; VQE: variational quantum eigensolver; EP: exceptional point; TI: time-independent quantum system; **a**: query depth accounts only for the parameters discussed in the corresponding references; **b**: it remains unknown whether VQE can solve non-Hermitian eigenproblems.

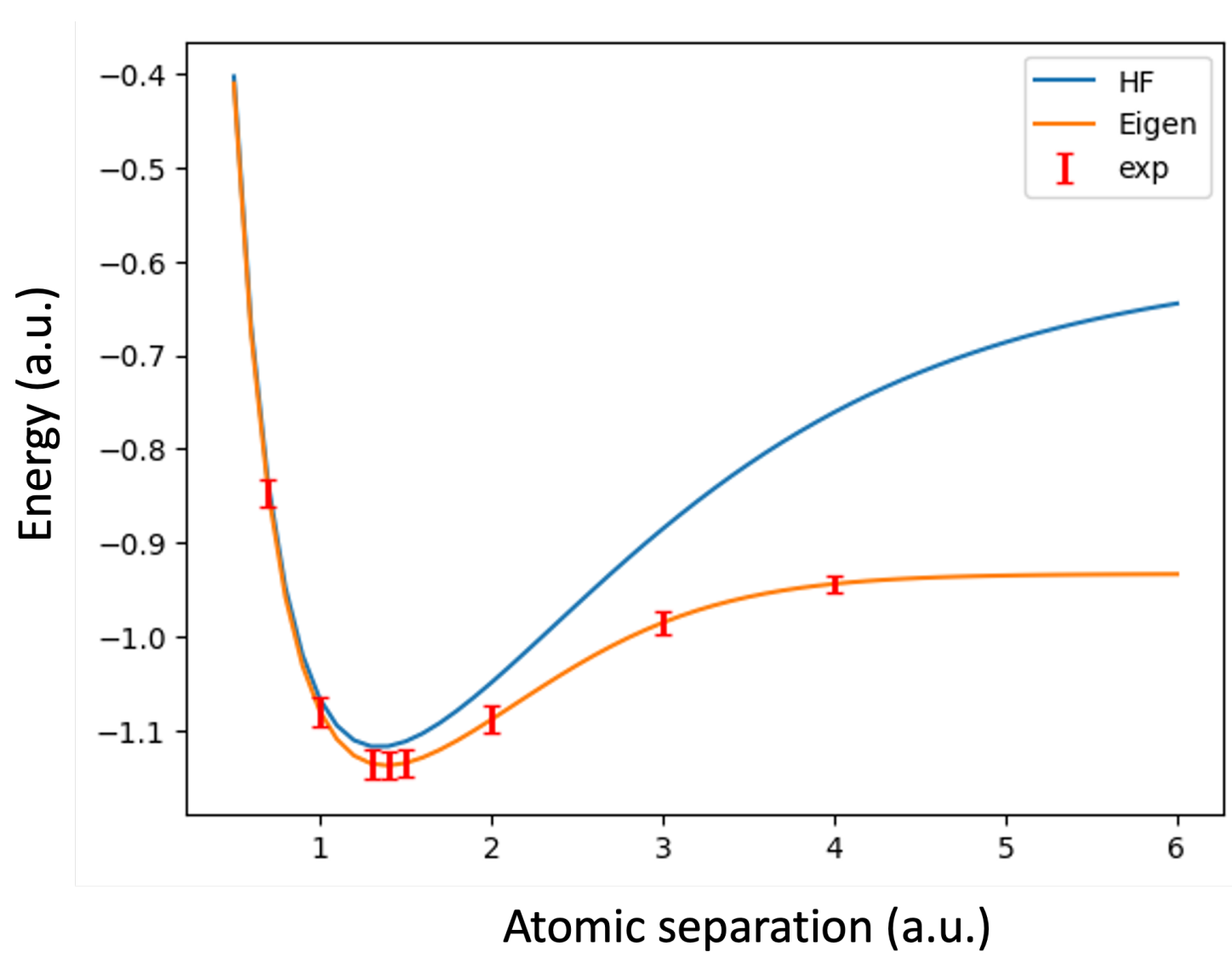

Supplementary Figure 7 The bond dissociation curve of hydrogen molecule estimated by QCS. The bar on each point denotes the range of Gaussian peaks in the QCS spectrum, not the estimation error. HF: Hartree-Fock ground-state energy; Eigen: exact ground-state energy of Jordan-Wigner transformed Hamiltonians; exp: experimental results by QCS.

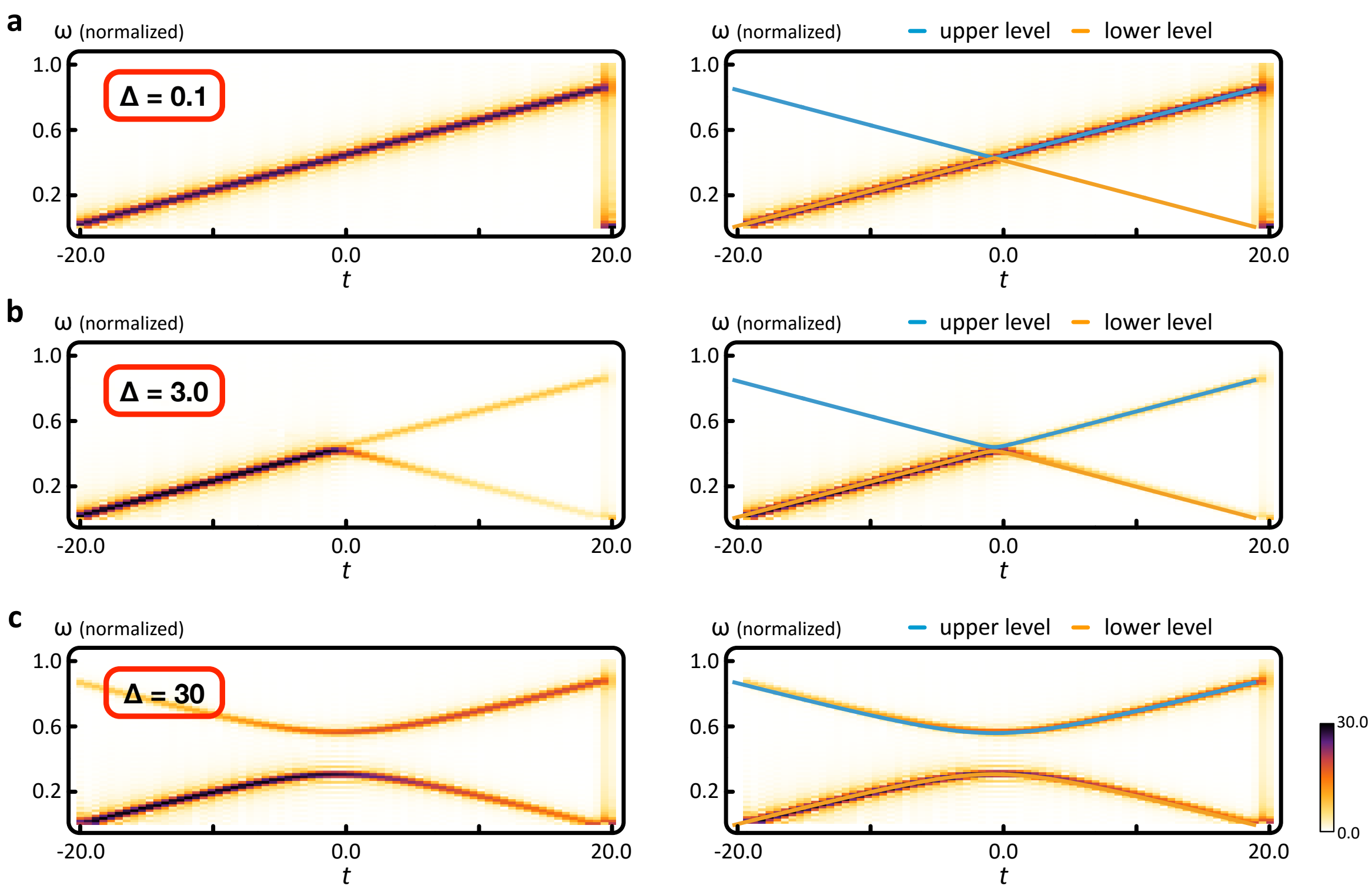

Supplementary Figure 8 Time-frequency analysis of the Landau-Zener model through simulated QCS. The Landau-Zener model describes the quantum dynamics of a two-level system driven by a linearly time-varying Hamiltonian $\hat{H}(t) = \frac{1}{2}\begin{pmatrix} vt & 2\Delta \\ 2\Delta & -vt \end{pmatrix}$, where v is the linear sweep rate ($v = 10.0$ in our case) and Δ is the constant coupling strength between the two levels. It interpret the aspects of the Rapid Adiabatic Passage (RAP) process in qubit manipulation, particularly the non-adiabatic transitions and transfer efficiency in two-level quantum systems during chirped frequency sweeps. Without coupling ($\Delta = 0$), the bare (diabatic) energy levels cross linearly at $t = 0$. Finite coupling opens an avoided crossing with a minimum energy gap of 2Δ. The instantaneous eigenenergies are $\pm\sqrt{(vt/2)^2 + \Delta^2}$. The panels show short-time Fourier transform (STFT) spectrograms (left) and corresponding theoretical adiabatic energy bands (right; blue: upper level, orange: lower level) for increasing coupling strengths. The eigenenergy estimation (ω) is normalized by the Nyquist-Shannon sampling frequency. The initial state is selected as the eigenstate at the lower energy level. The color of pixels denotes the intensity of STFT spectrum of $\mathcal{C}_{\hat{I},\psi}(t)$, indicated by the legend shown at the right bottom. **a,** Weak coupling ($\Delta = 0.1$): diabatic regime—the frequency trajectory closely follows the linear diabatic levels with almost no avoidance, resulting in the transition from the lower level to upper level. **b,** Intermediate coupling ($\Delta = 3.0$): transition regime—partial avoidance is visible as the trajectory begins to curve around $t = 0$. **c,** Strong coupling ($\Delta = 30$): near adiabatic regime—the bright ridge in the spectrogram precisely traces the hyperbolic adiabatic energy bands, reflecting strong avoidance of the crossing. The simulated results of QCS with STFT demonstrate excellent agreement with the theoretical time-varying eigenenergies and the transition behaviour of Landau-Zener model across the full range from diabatic (straight-line crossing) to adiabatic (wide hyperbolic avoidance) regime.

References

1. R. J. Marshman, A. P. Lund, P. P. Rohde & T. C. Ralph. Passive quantum error correction of linear optics networks through error averaging. *Phys. Rev. A* **97,** 022324 (2018).
2. N. Golyandina & A. Zhigljavsky. Singular Spectrum Analysis for Time Series (Springer Berlin Heidelberg, Berlin, Heidelberg, 2013).
3. E. Kökcü, H. A. Labib, J. K. Freericks & A. F. Kemper. A linear response framework for quantum simulation of bosonic and fermionic correlation functions. *Nat. Commun.* **15,** 3881 (2024).
4. J. Sun, L. Vilchez-Estevez, V. Vedral, A. T. Boothroyd & M. S. Kim. Probing spectral features of quantum many-body systems with quantum simulators. *Nat. Commun.* **16,** 1403 (2025).
5. H. H. S. Chan, R. Meister, M. L. Goh & B. Koczor. Algorithmic shadow spectroscopy. *PRX Quantum* **6,** 010352 (2025).
6. R. Somma, G. Ortiz, J. E. Gubernatis, E. Knill & R. Laflamme. Simulating physical phenomena by quantum networks. *Phys. Rev. A* **65,** 042323 (2002).
7. F. Minganti, A. Miranowicz, R. W. Chhajlany & F. Nori. Quantum exceptional points of non-Hermitian Hamiltonians and Liouvillians: The effects of quantum jumps. *Phys. Rev. A* **100,** 062131 (2019).
8. Y. Ashida, Z. Gong & M. Ueda. Non-Hermitian Physics. *Adv. Phys.* **69,** 249–435 (2020).
9. N. Maskara *et al.* Programmable simulations of molecules and materials with reconfigurable quantum processors. *Nat. Phys.,* 1–9 (2025).
10. M. S. Rudner & N. H. Lindner. Band structure engineering and non-equilibrium dynamics in Floquet topological insulators. *Nat. Rev. Phys.* **2,** 229–244 (2020).
11. B. M. Victor. Quantal phase factors accompanying adiabatic changes. *Proc. R. Soc. Lond. A* **392,** 45–57 (1984).
12. F. Wilczek & A. Zee. Appearance of Gauge Structure in Simple Dynamical Systems. *Phys. Rev. Lett.* **52,** 2111–2114 (1984).
13. J. Wang *et al.* Multidimensional quantum entanglement with large-scale integrated optics. *Science* **360,** 285–291 (2018).
14. J. Bao *et al.* Very-large-scale integrated quantum graph photonics. *Nat. Photon.* **17,** 573–581 (2023).
15. T. J. Seok, K. Kwon, J. Henriksson, J. Luo & M. C. Wu. Wafer-scale silicon photonic switches beyond die size limit. *Optica* **6,** 490–494 (2019).
16. Z. Du *et al.* Ultracompact and multifunctional integrated photonic platform. *Sci. Adv.* **10,** eadm7569 (2024).
17. S. Hong *et al.* Ultralow-loss compact silicon photonic waveguide spirals and delay lines. *Photon. Res.* **10,** 1–7 (2022).
18. C. Taballione *et al.* 20-Mode Universal Quantum Photonic Processor. *Quantum* **7,** 1071 (2023).
19. Y. Chi *et al.* A programmable qudit-based quantum processor. *Nat. Commun.* **13,** 1166 (2022).
20. M. Malik *et al.* Multi-photon entanglement in high dimensions. *Nat. Photon.* **10,** 248–252 (2016).
21. M. Erhard, M. Malik, M. Krenn & A. Zeilinger. Experimental Greenberger–Horne–Zeilinger entanglement beyond qubits. *Nat. Photon.* **12,** 759–764 (2018).
22. X.-M. Hu *et al.* Observation of Genuine High-dimensional Multi-partite Non-locality in Entangled Photon States. *Nat. Commun.* **16,** 5017 (2025).
23. H.-S. Zhong *et al.* 12-Photon Entanglement and Scalable Scattershot Boson Sampling with Optimal Entangled-Photon Pairs from Parametric Down-Conversion. *Phys. Rev. Lett.* **121,** 250505 (25 2018).
24. D. Llewellyn *et al.* Chip-to-chip quantum teleportation and multi-photon entanglement in silicon. *Nat. Phys.* **16,** 148–153 (2020).
25. C. Vigliar *et al.* Error-protected qubits in a silicon photonic chip. *Nat. Phys.* **17,** 1137–1143 (2021).
26. S. Paesani *et al.* Generation and sampling of quantum states of light in a silicon chip. *Nat. Phys.* **15,** 925–929 (2019).
27. J. M. Arrazola *et al.* Quantum circuits with many photons on a programmable nanophotonic chip. *Nature* **591,** 54–60 (2021).
28. S. Paesani *et al.* Near-ideal spontaneous photon sources in silicon quantum photonics. *Nat. Commun.* **11,** 2505 (2020).
29. F. Kaneda & P. G. Kwiat. High-efficiency single-photon generation via large-scale active time multiplexing. *Sci. Adv.* **5,** eaaw8586 (2019).
30. D. Cogan, Z.-E. Su, O. Kenneth & D. Gershoni. Deterministic generation of indistinguishable photons in a cluster state. *Nat. Photon.* **17,** 324–329 (2023).
31. Y. Meng *et al.* Deterministic photon source of genuine three-qubit entanglement. *Nat. Commun.* **15,** 7774 (2024).
32. S. Liu *et al.* Quantum correlations of spontaneous two-photon emission from a quantum dot. *Nature* **643,** 1234–1239 (2025).
33. K. Alexander *et al.* A manufacturable platform for photonic quantum computing. *Nature* **641,** 876–883 (2025).
34. S. Paesani, J. F. F. Bulmer, A. E. Jones, R. Santagati & A. Laing. Scheme for Universal High-Dimensional Quantum Computation with Linear Optics. *Phys. Rev. Lett.* **126,** 230504 (2021).
35. H. Aghaee Rad *et al.* Scaling and networking a modular photonic quantum computer. *Nature* **638,** 912–919 (2025).
36. M. V. Larsen *et al.* Integrated photonic source of Gottesman–Kitaev–Preskill qubits. *Nature* **642,** 587–591 (2025).
37. X. Jia *et al.* Continuous-variable multipartite entanglement in an integrated microcomb. *Nature* **639,** 329–336 (2025).
38. X. Jia *et al.* Monolithic integration of continuous-variable cluster-state generation, manipulation and measurement. *Nat. Photon.* **20,** 428–436 (2026).
39. M. Dobšíček, G. Johansson, V. Shumeiko & G. Wendin. Arbitrary accuracy iterative quantum phase estimation algorithm using a single ancillary qubit: A two-qubit benchmark. *Phys. Rev. A* **76,** 030306 (2007).
40. Y. Dong, L. Lin & Y. Tong. Ground-state preparation and energy estimation on early fault-tolerant quantum computers via quantum eigenvalue transformation of unitary matrices. *PRX Quantum* **3,** 040305 (2022).
41. A. Kitaev. Quantum measurements and the Abelian stabilizer problem. *Electron. Colloq. Comput. Complex.* **3** (1996).
42. A. Aspuru-Guzik, A. D. Dutoi, P. J. Love & M. Head-Gordon. Simulated Quantum Computation of Molecular Energies. *Science* **309,** 1704–1707 (2005).

43. A. Peruzzo *et al.* A variational eigenvalue solver on a photonic quantum processor. *Nat. Commun.* **5,** 4213 (2014).
44. D. Wu *et al.* Variational benchmarks for quantum many-body problems. *Science* **386,** 296–301 (2024).
45. M. Cerezo *et al.* Variational quantum algorithms. *Nat. Rev. Phys.* **3,** 625–644 (2021).
